# Aryl Bromides and Aryl Chlorides for the Direct Arylation of Benzylic Amines Mediated by Ruthenium(II)

**DOI:** 10.1002/ejoc.201300004

**Published:** 2013-03-22

**Authors:** Navid Dastbaravardeh, Michael Schnürch, Marko D Mihovilovic

**Affiliations:** [a] Institute of Applied Synthetic Chemistry, Vienna University of TechnologyGetreidemarkt 9/163-OC, 1060 Vienna, Austria, Fax: +43-1-58801-9163616

**Keywords:** Homogeneous catalysis, C–H activation, Cleavage reactions, Reaction mechanisms, Isotope effects

## Abstract

The ruthenium(II)-catalyzed sp^3^ C–H bond arylation of benzylic amines with aryl halides is reported. In the present method, aryl iodides and, more importantly, also the cheaper aryl bromides and aryl chlorides can be applied as aryl sources. Additionally, the method does not require elaborate manipulations in a glove box and can be carried out in simple screw cap vials. Potassium pivalate proved to be beneficial for the transformation with aryl bromides or iodides as aryl source, but was not required for aryl chlorides. In the latter case, the addition of PPh_3_ led to high conversion. 3-Methyl and 3-phenyl pyridine were established as directing groups, and the substituent in the 3-position represents a key structural feature for high conversion. The directing group can be cleaved after the transformation, which allows access to diarylmethylamines. Mechanistic studies were carried out and critically compared to mechanistic reports of related transformations.

## Introduction

Carbon–carbon bond formation is a central part of many chemical syntheses, and nowadays there is a vast number of ways for the formation of this kind of bond. Transition-metal catalyzed cross-coupling reactions are one of the most frequently applied methods for the creation of new C–C bonds.^1^ However, the required organometallic nucleophilic reagents, particularly those that are functionalized, are often not commercially available or are relatively expensive. One way to overcome this problem is to introduce new functional groups directly through transformation of C–H bonds, which unlocks opportunities for markedly different synthetic strategies. Thus, transition-metal-catalyzed functionalization of hydrocarbons is one of the most frequently investigated but also one of the most challenging topics in modern organic synthesis.^2^ The development of new synthetic methods and innovations in these types of reactions will profoundly improve overall synthetic efficiency. The possibility of direct formation of a new carbon–carbon bond by C–H bond transformation is a highly attractive strategy in covalent synthesis, owing to the ubiquitous nature of C–H bonds in organic substances and the high atom economy of the process.

Regioselective direct arylations are difficult to achieve because the arene reagents often contain several nonequivalent C–H bonds that can react with the metal center at a similar rate. This selectivity problem usually furnishes undesired side products. The electronic properties of the substrate can control the position of C–H bond cleavage.^3^ These electronic properties can be difficult to override and limit the scope of reagents. There are several approaches to overcome this problem and the most common strategy for conducting regioselective direct arylations involves the use of substrates containing directing groups. Ligating substituents can direct the metal center to cleave a specific C–H bond to form a five- or six-membered metallacycle.^4^ Despite the success in this area, there are relatively few studies on the direct functionalization of sp^3^ carbon centers.^5^

We recently reported a Ru^0^-catalyzed chelation-assisted method for the direct arylation of benzylic amines.^6^ Our preliminary studies in this area focused on the identification of an appropriate directing group. Notably, we found that 3-substituted pyridine displayed the best activity owing to the steric properties of this group. However, this protocol was limited to boronic acid esters, and other aryl sources, most importantly aryl halides, were not tolerated. Hence, we were interested in the investigation of alternative methods suitable for aryl halides, and we developed a Ru^II^-catalyzed method that enabled the use of aryl bromides and aryl iodides as arylation reagents.^7^ Aryl chlorides were not suitable for this kind of transformation. Within the present contribution, we describe the expansion of substrate scope of our previously reported method and disclose a new synthetic procedure, which also enables the use of aryl chlorides. Mechanistic investigations indicated that the two protocols proceed by different mechanistic pathways.

## Results and Discussion

The initial inspiration for the development of an arylation protocol that uses aryl halides came from a publication of Ackermann and co-workers who reported a ruthenium-catalyzed cyclometalation method for the direct arylation of sp^2^ carbon centers with aryl halides.^8^ [RuCl_2_(*p*-cymene)]_2_ is a frequently used catalyst for the direct functionalization of unactivated sp^2^ C–H bonds and a variety of catalytic reactions have been developed during recent years.^9^ We envisaged that this method would also be applicable to our benzylic system, although direct sp^3^ arylation was unprecedented with this catalyst at that time. We initiated our optimization studies with 1 equiv. of *N*-benzyl-3-methylpyridin-2-amine (**1a**), 1.5 equiv. of bromobenzene, 2.5 mol-% of [RuCl_2_(*p*-cymene)]_2_, and 3 equiv. of K_2_CO_3_ in 2 mL of toluene. The reaction mixture was stirred for 24 h at 140 °C. Under these conditions, the desired product **3a** was formed (Table 1, Entry 1), but only in 34 % yield. Interestingly, we could also detect the corresponding dehydrogenated imine derivative **4** as a major side product, although the reaction was performed under an inert atmosphere in the absence of an oxidant. We also tested other catalysts known to undergo C–H activation in combination with different additives (Table 1, Entries 2–7).^10^ Products **3a** and **4a** were detected simultaneously in reactions that gave noteworthy conversions (Table 1, Entries 1–3), however in different amounts. The ratio of amine to imine product was obviously dependant on the reaction conditions, in particular on the catalyst species. Amongst the investigated complexes, the initially used [RuCl_2_(*p*-cymene)]_2_ showed the best activity and also the highest amine-to-imine ratio. In a first series of experiments, we tested whether additives such as potassium pivalate (KOPiv) and PPh_3_ showed beneficial effects on the yields and also if they suppressed imine formation. The addition of carboxylates can facilitate C–H bond activation by promoting a concerted metalation deprotonation (CMD) mechanism.^11^ Indeed, the addition of KOPiv led to a significant higher yield of 75 % (Table 1, Entry 8). PPh_3_ also increased the activity of the catalyst, but we decided to continue with KOPiv owing to its slightly better performance. Bromo- and iodobenzene showed good conversion but chlorobenzene was not suitable for this method.

**Table 1:** Optimization studies for the direct arylation of benzylic amine 1a[a]

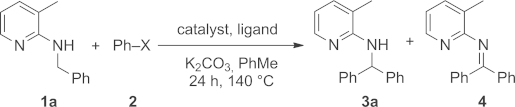						

Entry	Catalyst	Ligand	X	Conv. [b]	3a/4[c]	Yield of3a[d]
1	[RuCl_2_(*p*-cymene)]_2_	–	Br	59	4.0	34
2	RuCl_3_**·**(H_2_O)*_n_*	–	Br	28	3.5	17
3	RuCl_2_(PPh_3_)_3_	–	Br	47	2.4	27
4	[RhCl(cod)]_2_	–	Br	8	–	5
5	[RhCl(C_2_H_4_)]_2_	–	Br	8	–	4
6	[RhCp*Cl_2_]_2_	–	Br	6	–	4
7	Rh_4_(CO)_12_	–	Br	0	–	0
8	[RuCl_2_(*p*-cymene)]_2_	KOPiv	Br	98	6.0	75
9	RuCl_3_**·**(H_2_O)*_n_*	KOPiv	Br	0	–	0
10	RuCl_2_(PPh_3_)_3_	KOPiv	Br	0	–	0
11	[RuCl_2_(*p*-cymene)]_2_	PPh_3_	Br	85	4.6	51
12	RuCl_3_**·**(H_2_O)*_n_*	PPh_3_	Br	0	–	0
13	RuCl_2_(PPh_3_)_3_	PPh_3_	Br	37	2.2	20
14	[RuCl_2_(*p*-cymene)]_2_	KOPiv	Cl	8	–	4
15	[RuCl_2_(*p*-cymene)]_2_	KOPiv	I	88	30	57

[a] Reaction conditions: **1a** (0.5 mmol), PhX (0.75 mmol), catalyst (2.5 mol-%), KOPiv (30 mol-%) or PPh_3_ (5 mol-%), K_2_CO_3_ (1.5 mmol), and PhMe (2 mL).

[b] Conversion determined by GC analysis with respect to **1a**.

[c] Ratio based on GC analysis.

[d] Yield determined by GC analysis with respect to **1a** (dodecane as internal standard).

Subsequently, the scope of pyridine-substituted benzylamines **1** to react with aryl bromide and iodide derivatives was examined. This catalytic method showed a similar behavior to our previously reported ruthenium(0)-catalyzed method with respect to the steric and electronic properties of the aryl donor species. Sterically demanding *ortho*-substituted aryls (2-Me 18 % and 1-naphthyl 14 %; Table 2, Entries 3 and 4) gave significantly lower conversions, but *meta*-substituted aryls showed good conversions and yields (3-Me 55 %, 3-OMe 60 %, 3-Cl 37 %; Table 2, Entries 5–7). Electron-neutral or -donating aryl groups (Table 2, Entries 8–13) could be applied with the best results, whereas strong electron-withdrawing or coordinating substituents (Table 2, Entries 17–22) were much less tolerated. The phenyl substituent at the 3-position of pyridine **1b** showed slightly better yields at a higher temperature (150 °C, see Table 2, Entries 23–33). By employing this bulky group, even the electron-withdrawing 4-MeCO substituent in the aryl donor was converted with 41 % yield (Table 2, Entry 31).

**Table 2:** Scope of arylation of benzylic amine 1.[a]

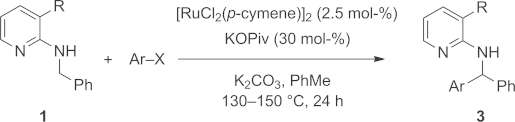							

Entry	1	R	X	Ar	3	Conv.[b]	Yield
1	**1a**	Me	Br	C_6_H_5_	**3a**	96	69
2	**1a**	Me	I	C_6_H_5_	**3a**	100	48
3	**1a**	Me	Br	2-Me-C_6_H_4_	**3b**	18	n.i.[c]
4	**1a**	Me	Br	1-naphthyl	**3c**	14	n.i.[c]
5	**1a**	Me	Br	3-Me-C_6_H_4_	**3d**	98	55
6	**1a**	Me	Br	3-MeO-C_6_H_4_	**3e**	97	60
7	**1a**	Me	Br	3-Cl-C_6_H_4_	**3f**	60	37
8	**1a**	Me	Br	4-Me-C_6_H_4_	**3g**	98	65
9	**1a**	Me	Br	4-*t*Bu-C_6_H_4_	**3h**	96	64
10	**1a**	Me	Br	4-*n*Bu-C_6_H_4_	**3i**	98	67
11	**1a**	Me	Br	4-MeO-C_6_H_4_	**3j**	95	63
12	**1a**	Me	I	4-MeO-C_6_H_4_	**3j**	98	61
13	**1a**	Me	Br	4-Me_2_N-C_6_H_4_[d]	**3k**	94	50
14	**1a**	Me	Br	4-F-C_6_H_4_	**3l**	92	61
15	**1a**	Me	I	4-F-C_6_H_4_	**3l**	97	55
16	**1a**	Me	Br	4-Cl-C_6_H_4_	**3m**	98	51
17	**1a**	Me	Br	4-EtO_2_C-C_6_H_4_	**3n**	72	33
18	**1a**	Me	Br	4-MeOC-C_6_H_4_	**3o**	15	n.i.[c]
19	**1a**	Me	Br	4-O_2_N-C_6_H_4_	**3p**	0	–
20	**1a**	Me	Br	4-NC-C_6_H_4_	**3q**	0	–
21	**1a**	Me	Br	3-pyridyl	**3r**	0	–
22	**1a**	Me	Br	2-thienyl	**3s**	0	–
23	**1b**	C_6_H_5_	Br	C_6_H_5_[e]	**3t**	98	70
24	**1b**	C_6_H_5_	Br	3-Me-C_6_H_4_[e]	**3u**	97	68
25	**1b**	C_6_H_5_	Br	3-MeO-C_6_H_4_[e]	**3v**	95	64
26	**1b**	C_6_H_5_	Br	4-Me-C_6_H_4_[e]	**3w**	97	67
27	**1b**	C_6_H_5_	Br	4-*t*Bu-C_6_H_4_[e]	**3x**	98	72
28	**1b**	C_6_H_5_	Br	4-*n*Bu-C_6_H_4_[e]	**3y**	97	69
29	**1b**	C_6_H_5_	Br	4-Cl-C_6_H_4_[e]	**3z**	81	59
30	**1b**	C_6_H_5_	Br	4-EtO_2_C-C_6_H_4_[e]	**3aa**	64	42
31	**1b**	C_6_H_5_	Br	4-MeOC-C_6_H_4_^e]^	**3ab**	65	41
32	**1b**	C_6_H_5_	Br	4-O_2_N-C_6_H_4_[e]	**3ac**	0	–
33	**1b**	C_6_H_5_	Br	4-NC-C_6_H_4_[e]	**3ad**	0	–

[a] Reaction conditions: **1** (0.5 mmol), ArX (0.75 mmol), [RuCl_2_(*p*-cymene)]_2_ (2.5 mol-%), KOPiv (30 mol-%), K_2_CO_3_ (1.5 mmol), and PhMe (2 mL).

[b] Conversion determined by GC analysis with respect to **1**.

[c] n.i. = not isolated.

[d] 130 °C.

[e] 150 °C.

Next, we were interested in the influence of the electronic effects of functional groups incorporated into the benzylic group. Thus, we varied the benzylic group of our starting material and performed the reaction under the above outlined standard conditions. To exclude steric effects, functional groups were only installed at the *para* position. The results are in accordance with those with the ruthenium(0) series and indicate that electron-neutral groups perform best (Table 3, Entry 4).^6^ However, this method gives better results with electron-withdrawing substituents than with electron-donating substituents, which is contrary to the ruthenium(0) method.^6^ We could not detect any decarboxylation with starting material **1h** (Table 3, Entry 7), as was the case within the ruthenium(0) method.^6^

**Table 3:** Influence of the substituent on the benzylic group for the Ru-catalyzed direct arylation[a]

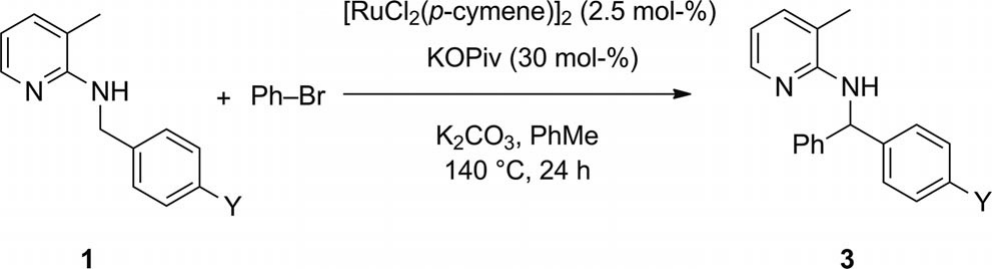					

Entry	1	Y	3	Conv.[b]	Yield
1	**1c**	OMe	**3j**	49	28
2	**1d**	O*i*Pr	**3ae**	75	43
3	**1e**	Me	**3g**	77	48
4	**1a**	H	**3a**	96	69
5	**1f**	F	**3l**	85	59
6	**1g**	CF_3_	**3af**	97	57
7	**1h**	CO_2_Me	**3ag**	88	57

[a] Reaction conditions: **1** (0.5 mmol), PhBr (0.75 mmol), [RuCl_2_(*p*-cymene)]_2_ (2.5 mol-%), KOPiv (30 mol-%), K_2_CO_3_ (1.5 mmol), and PhMe (2 mL).

[b] Conversion determined by GC analysis with respect to **1**.

Competition experiments between differently substituted starting materials were carried out to validate the results presented in Table 3. We used an equimolar mixture of unsubstituted and *para*-substituted starting material with our optimized reaction conditions; this mixture was treated with 1 equiv. of bromobenzene, a decreased amount of aryl source in comparison to previous experiments to ensure incomplete conversion of both substrates. Only then does the obtained product distribution give meaningful results, which are shown in Table 4. Weak electron-withdrawing substituents such as F or CF_3_ (Table 4, Entries 4 and 5) react faster than strong electron-donating and -withdrawing groups (Table 4, Entries 1, 2, and 6). These findings corroborate the results shown in Table 3, and the overall performance of the systems is complementary to the results with Ru^0^ catalysis.^6^

**Table 4:** Competitive experiments for the Ru-catalyzed direct arylation reaction[a]

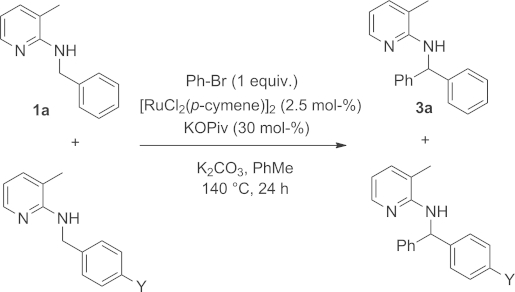		

Entry	Y	H/Y[b]
1	OMe	2
2	O*i*Pr	1.3
3	Me	1.1
4	F	1.1
5	CF_3_	0.9
6	CO_2_Me	1.8

[a] Reaction conditions: **1a** (0.5 mmol), substituted amine (0.5 mmol), PhBr (0.5 mmol), [RuCl_2_(*p*-cymene)]_2_ (2.5 mol-%), KOPiv (30 mol-%), K_2_CO_3_ (1.5 mmol), and PhMe (2 mL).

[b] Ratio determined by GC analysis.

**Scheme 1:**
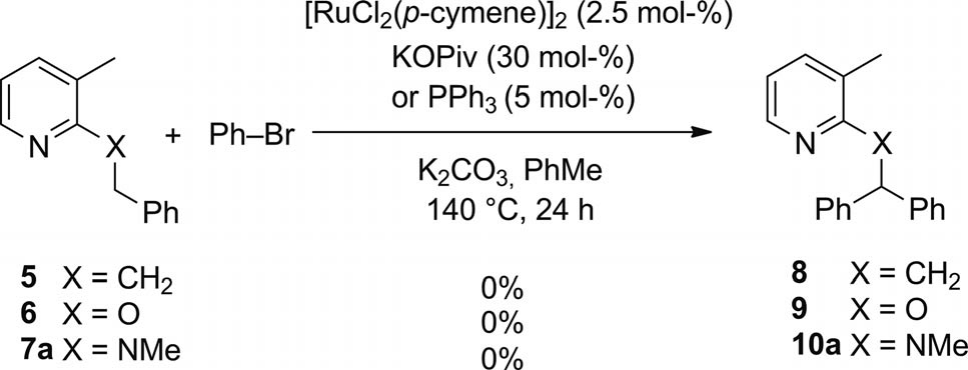
Direct arylation of **5**, **6**, and **7a**.

In the next step, we wanted to investigate the role of the nitrogen atom adjacent to the C–H bond. Therefore, we substituted the nitrogen atom with a CH_2_ group (**5**) or oxygen atom (**6**). In the ruthenium(0) protocol, the presence of an oxygen center was detrimental, but CH_2_ gave a good yield. In the ruthenium(II) protocol, both substituents were not suitable for this transformation, which indicates that the ruthenium(II) mechanism is completely different from the ruthenium(0) mechanism and requires a nitrogen atom in this position (Scheme 1).

**Scheme 2:**
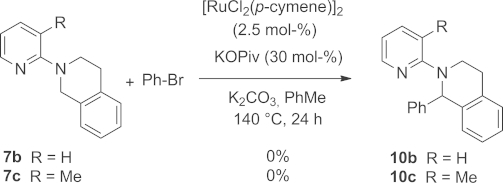
Direct arylation of *N*-substituted THIQ.

The last experiments inspired us to test whether a free NH group is essential for this transformation. We performed the reaction with the NMe-benzylic amine **7a** (Scheme 1). In contrast to the ruthenium(0) system, only the free amines showed any conversion, and all other substrates were not tolerated. Hence, we conclude that the free amine function is essential for the ruthenium(II)-catalyzed transformation. This conclusion is also supported by the findings presented in Scheme 2. Tetrahydroisoquinoline (THIQ) substrates **7b** and **7c** did not show any conversion. Hence, the predominant geometry of substrate **7a**, which disfavors arylation, can be excluded as the reason for substrate **7a** to fail in this reaction. If this were the case, compounds **7b** and **7c** would react at least to some extent.

**Scheme 3:**
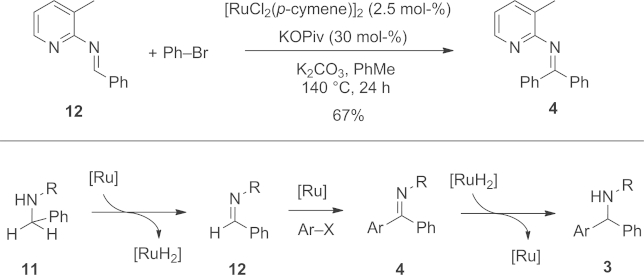
Hypothesis for imine formation and ruthenium(II)-catalyzed direct arylation of **12**.

One explanation for the mandatory presence of a free NH group could be that the mechanism does not proceed by direct sp^3^ C–H insertion of the metal center but rather by dehydrogenation of the amine to the corresponding imine. The imine formed can further react in a subsequent arylation step to form imine product **4**, which is most likely in equilibrium with the desired product. This equilibrium explains the detection of imine **4** in the reaction. We conducted an experiment with the already dehydrogenated benzylic imine **12** to investigate this hypothesis. As expected, we isolated the imine compound **4** (67 % yield, Scheme 3). The fact that **4** was not reduced to **3** in this experiment suggests that the hydrogen required for reduction originates from a [RuH_2_] species. This species is produced by the dehydrogenation of **11** to form **12**. It seems that [RuH_2_] stays closely associated with **4** and immediately induces the reduction to **3**. Furthermore, Jun and co-workers have shown that **12** can be arylated with Ru_3_(CO)_12_ and phenyl boronic acid ester.^12^

**Scheme 4:**
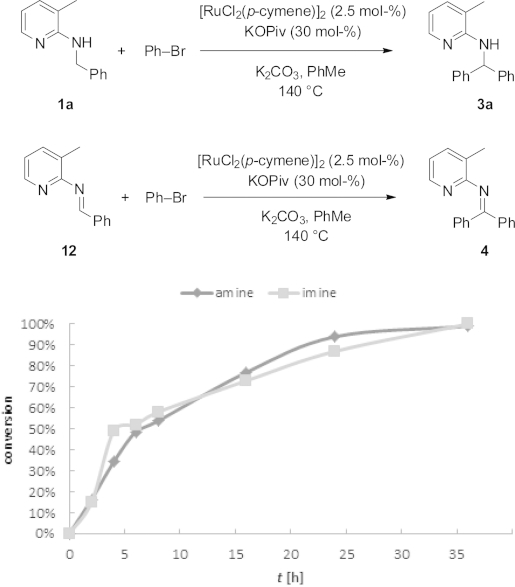
Kinetic measurements for the ruthenium(II)-catalyzed direct arylation of **1a** and **12**.

We were also interested in a comparison of the rate of both the arylation of amine **1a** and of imine **12**. To this end, we performed kinetic studies for both derivatives. We found that the rate of arylation of imine **12** was in the same range as that of the arylation of amine **1a**. This could either be coincidental or be due to a fast (not rate determining) formation of imine **12** from amine **1a**. In the latter case, the reduction of **4** to **3a** also has to be fast (i.e., not rate determining). Alternatively, **4** could be formed after arylation from **3a** by metal-catalyzed dhydrogenation; this could be tested by submitting **1a** and **3a** to the reaction conditions in the absence of bromobenzene. Interestingly, in both cases only trace amounts of the corresponding imines were formed. Evidently, the aryl halide is also involved in the dehydrogenation process. Based on these results, we cannot determine whether the arylation takes place on the amine or imine compound; at this stage of our studies we favor mechanisms that do not include imine formation (Scheme 4).

Finally, we performed the reaction under different atmospheres as this could provide mechanistic information. We have found the catalyst to be stable under air in the ruthenium(0) protocol and that it performs even better under a H_2_ atmosphere.^6^ In the ruthenium(II) case, the catalyst performs slightly worse under air and significantly worse under hydrogen (Table 5, Entries 2 and 4). Eventually, H_2_ partially transforms the catalyst to an inactive species. Alternatively, the oxidation of the amine to the imine might be hindered under a H_2_ atmosphere if the reaction proceeds by initial imine formation and arylation thereof. Furthermore, the reaction does not proceed under CO, which can be attributed to the strong binding character of the CO ligand (Table 5, Entry 3), which leads to catalyst inactivation. The reaction can be carried out under microwave irradiation, which significantly decreases the reaction time from 24 h to 2.5 h with similar yield.

**Table 5:** Ru-catalyzed arylation of 1a under different atmospheres[a]

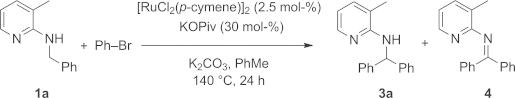				

Entry	Atmosphere	Conv.[b]	3a/4[c]	Yield[d]
1	argon	96	6.0	75 (69)[e]
2	air	79	4.0	56
3	CO	0	–	0
4	H_2_	53	8.3	37
5	argon (microwave)[f]	92	5.1	66

[a] Reaction conditions: **1a** (0.5 mmol), PhBr (0.75 mmol), [RuCl_2_(*p*-cymene)]_2_ (2.5 mol-%), KOPiv (30 mol-%), K_2_CO_3_ (1.5 mmol), and PhMe (2 mL).

[b] Conversion determined by GC analysis with respect to **1a**.

[c] Ratio determined by GC.

[d] Yield determined by GC analysis with respect to **1a** (dodecane as internal standard).

[e] Number in parentheses is yield of **3a**.

[f] Microwave conditions: 180 °C for 2.5 h.

**Scheme 5:**
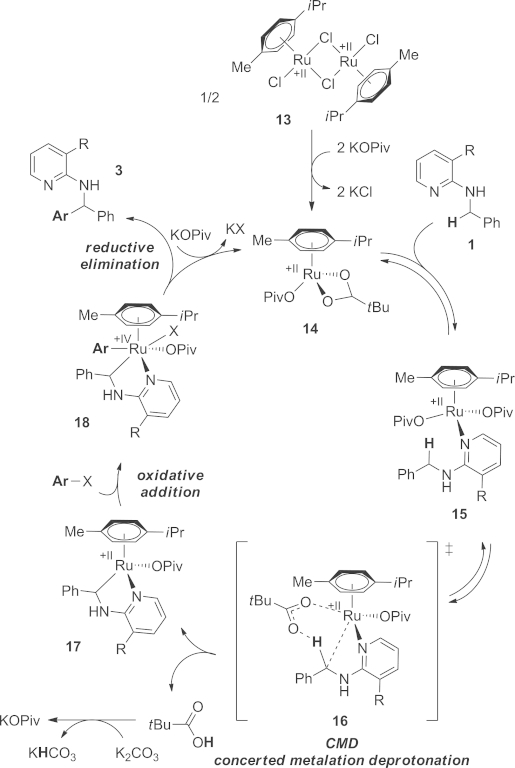
Proposed mechanism for the ruthenium(II)-catalyzed reaction.

We also performed kinetic isotope effect (KIE) experiments to determine whether the C–H activation step is rate-limiting. A KIE of 1.3 was found, which indicates that C–H insertion of the metal center is not the rate-determining step in this reaction, as otherwise a much higher KIE would be expected.^13^ This result is in contrast to the previously observed Ru_3_(CO)_12_/phenylboronic acid ester protocol, which displays a KIE of 3.3.^6^ Consequently, we also carried out an intramolecular competition experiment. Here, the KIE was found to be 1, which is again in contrast to the Ru_3_(CO)_12_ protocol (KIE = 0.43) and indicates again that C–H insertion is not rate-determining and is most likely also irreversible. As experiments to form imine **12** from substrate **1a** failed, we hypothesize that the mechanism does not include imine formation prior to arylation. However, at present only a speculative discussion of the reaction mechanism is possible, which is in line with the work of Ackermann11b and Jutand and Dixneuf.11c The most probable mechanism involves the carboxylatoruthenium(II) complex **14**, which is formed from [RuCl_2_(*p*-cymene)]_2_ in the presence of KOPiv. This complex undergoes cyclometalation with **1** to form intermediate **15**. Subsequent CMD via transition state **16** delivers the ruthenium(II) complex **17**, followed by oxidative addition of the aryl halide to the ruthenium(IV) species **18**. Final reductive elimination yields product **3**, and the ruthenium(II) complex **14** is regenerated; complex **14** can now reenter the next catalytic cycle (Scheme 5).

**Scheme 6:**
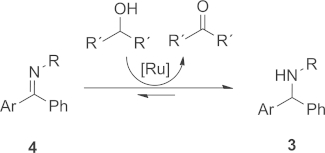
Role of secondary alcohol.

Additionally, we were also interested in the expansion of the arylation reaction to aryl chlorides. These precursors showed very little conversion under the standard conditions developed for aryl bromides and iodides. Further fine tuning of the protocol was attempted to make this compound class also accessible for direct sp^3^ arylation. The reactions of aryl chlorides have already been the target of catalyst development in cross-coupling chemistry because they are less expensive than aryl bromides and more derivatives are commercially available.^14^ In the field of direct arylations of sp^3^ C–H bonds, only a few examples have been reported that take advantage of aryl chlorides, usually in combination with Pd catalysts.^15^ We started our screening with our initial conditions and changed the ligand in a first series of experiments. A low yield of 12 % was achieved in the absence of carboxylate (Table 6, Entry 1), whereas only 4 % yield was detected in the presence of carboxylate (Table 6, Entries 2 and 3). Next, we tested different kinds of phosphane ligands as Oi and co-workers had already demonstrated their favorable effect on the [RuCl_2_(*p*-cymene)]_2_ catalyst system.^16^ In these cases, an increased yield for all investigated phosphanes (Table 6, Entries 4–12) was observed. The best result was obtained when using simple PPh_3_, however in this case a 38 % GC yield (Table 6, Entry 4) could not be surpassed. Other electron-rich, electron-poor, or sterically-demanding phosphanes also showed an enhancement of GC yield but were less effective (Table 6, Entries 5–12). The addition of bidentate BINAP ligand decreased the conversion (Table 6, Entry 13). The N-heterocyclic carbene (NHC) ligand IMes**·**HCl did not show a significant positive effect (Table 6, Entry 14). Hence, we decided to continue optimization with PPh_3_ as ligand. One main problem for the transformation of aryl chlorides has been the high concomitant imine formation. This imine formation can be explained by ruthenium-catalyzed dehydrogenation of the product.^17^ Unfortunately, when using aryl chlorides we could not get a better amine-to-imine ratio than 2.9 (Table 6, Entry 10) which is significantly lower than that obtained with aryl bromides (6.0, Table 1, Entry 8). In the ruthenium(0) reaction, we found the dissociated hydrogen to be successfully scavenged by the ketone with concomitant reduction to the alcohol.^6^ Hence, we hypothesized the possibility of a reverse pathway: the addition of an alcohol should deliver the required hydrogen, which might reduce the imine in situ (Scheme 6).

**Table 6:** Optimization studies for the direct arylation of benzylic amine 1a with aryl chlorides[a]

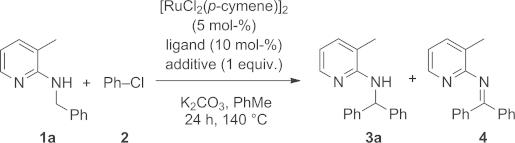					

Entry	Ligand	Additive	Conv.[b]	3a/4[c]	Yield of3a[d]
1	–	–	34	1.8	12
2	KOPiv	–	8	–	4
3	AdCO_2_K	–	7	–	4
4	PPh_3_	–	81	1.9	38
5	P(o-Tol)_3_	–	64	2.3	32
6	P(4-OMe-Ph)_3_	–	61	2.5	33
7	P(4-Cl-Ph)_3_	–	50	1.6	19
8	P(Cy)_3_	–	60	1.5	28
9	XPhos	–	62	2.1	30
10	JohnPhos	–	51	2.9	26
11	RuPhos	–	56	1.4	23
12	DavePhos	–	66	1.5	29
13	BINAP	–	9	2.6	4
14	IMes**·**HCl	–	45	0.8	11
15	PPh_3_	*i*PrOH	17	> 100[e]	12
16	PPh_3_	3-pentanol	26	> 100[e]	18
17	PPh_3_	cyclopentanol	9	> 100[e]	6
18	PPh_3_	cyclohexanol	49	> 100[e]	38
19	PPh_3_	cyclohexanol	93	12	79 (70)[f][g]

[a] Reaction conditions: **1a** (0.5 mmol), PhCl (1.5 mmol), [RuCl_2_(*p*-cymene)]_2_ (5 mol-%), ligand (10 mol-%), additive (0.5 mmol), K_2_CO_3_ (1.5 mmol), and PhMe (2 mL).

[b] Conversion determined by GC analysis with respect to **1a**.

[c] Ratio based on GC analysis.

[d] Yield determined by GC analysis with respect to **1a** (dodecane as internal standard).

[e] No **4** detected.

[f] 160 °C for 30 h in *o*-xylene.

[g] Number in parentheses is yield of **3a**.

We were pleased to discover that the addition of secondary alcohols led to a high amine-to-imine ratio (Table 6, Entries 15–18). Cyclohexanol was more effective (38 %, Table 6, Entry 18) than other alcohols such as *i*PrOH and 3-pentanol (Table 6, Entries 15 and 16). Although this additive did not improve the overall transformation (cf. Table 6, Entry 4), its presence led to the exclusive formation of amine product **3a**. Notably, we could detect the corresponding cyclohexanone by GC–MS analysis. Finally, conducting the reaction at 160 °C for 30 h (with *o*-xylene as solvent) furnished 70 % yield of product **3a** (Table 6, Entry 19).

**Scheme 7:**
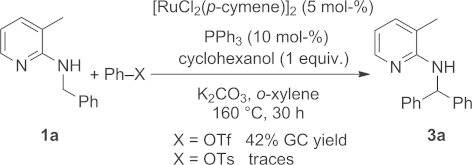
Direct arylation of **1a** with aryl triflate and tosylate.

Furthermore, this catalytic system is not restricted to halides, and triflates were also accepted, which was not the case in the presence of KOPiv (tosylates were in both cases not tolerated, Scheme 7). Unfortunately, the GC yield was only modest, and the procedure requires additional optimization for synthetic utilization.

The corresponding aryl chlorides showed analogous substrate scope to the bromide/iodide protocol, albeit the reaction conditions are harsher (Table 7, Entries 1–5). In this case, the reaction is obviously again sensitive to electron-withdrawing substituents (Table 7, Entries 6 and 7). Interestingly, phenyl-substituted pyridine precursor **1b** showed lower conversion for this specific method (Table 7, Entries 8–16). We assume that the more bulky phenyl substituent is less tolerated by the in-situ-formed complex in this case.

**Table 7:** Scope of arylation of benzylic amine 1 with aryl chlorides[a]

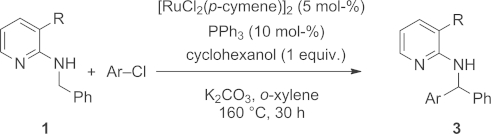						

Entry	1	R	Ar	3	Conv.[b]	Yield
1	**1a**	Me	C_6_H_5_	**3a**	93	70
2	**1a**	Me	3-Me-C_6_H_4_	**3d**	95	72
3	**1a**	Me	4-Me-C_6_H_4_	**3g**	93	79
4	**1a**	Me	4-MeO-C_6_H_4_	**3j**	88	64
5	**1a**	Me	4-F-C_6_H_4_	**3l**	76	56
6	**1a**	Me	4-F_3_C-C_6_H_4_	**3af**	79	30
7	**1a**	Me	4-MeO_2_C-C_6_H_4_	**3ag**	23	n.i.[c]
8	**1b**	C_6_H_5_	C_6_H_5_	**3t**	60	48
9	**1b**	C_6_H_5_	3-Me-C_6_H_4_	**3u**	68	58
10	**1b**	C_6_H_5_	3-MeO-C_6_H_4_	**3v**	72	61
11	**1b**	C_6_H_5_	4-Me-C_6_H_4_	**3w**	58	39
12	**1b**	C_6_H_5_	4-*t*Bu-C_6_H_4_	**3x**	69	55
13	**1b**	C_6_H_5_	4-*n*Bu-C_6_H_4_	**3y**	55	47
14	**1b**	C_6_H_5_	4-MeCO-C_6_H_4_	**3ab**	38	n.i.[c]
15	**1b**	C_6_H_5_	4-MeO_2_C-C_6_H_4_	**3ah**	16	n.i.[c]
16	**1b**	C_6_H_5_	4-O_2_N-C_6_H_4_	**3ac**	0	0

[a] Reaction conditions: **1** (0.5 mmol), ArCl (1.5 mmol), [RuCl_2_(*p*-cymene)]_2_ (5 mol-%), PPh_3_ (10 mol-%), cyclohexanol (0.5 mmol), K_2_CO_3_ (1.5 mmol), and PhMe (2 mL).

[b] Conversion determined by GC analysis with respect to **1**.

[c] n.i. = not isolated.

**Scheme 8:**
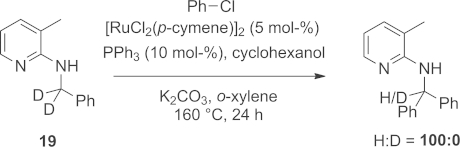
KIE control experiment with **19**.

Notably, the corresponding imine starting material **12** was not converted under these conditions, which indicates that the arylation process occurs directly on the C–H bond of the starting material **1a**, and the imine compound **4** is subsequently formed from amine product **3a** by dehydrogenation. The mechanism of the [RuCl_2_(*p*-cymene)PPh_3_] catalyst is obviously not exactly the same as for the [Ru(*p*-cymene)(OPiv)_2_] complex (the lack of carboxylate allows no CMD mechanism). Finally, we also wanted to conduct intermolecular and intramolecular KIE experiments with the [RuCl_2_(*p*-cymene)]_2_/PPh_3_/chlorobenzene/cyclohexanol system to obtain more information about the mechanism of the reaction. However, we observed a high ruthenium-catalyzed H/D exchange of the substrates under these conditions. In an intermolecular competition experiment, only an arylated product that contained hydrogen atoms but not deuterium atoms was detected. The same result was found in the intramolecular competition experiment. This would mean that only the C–D bond is broken, which is highly unlikely. We also isolated the substrates from these experiments, which also contained only hydrogen atoms and no deuterium atoms. Most likely, the exchange is caused by the cyclohexanol present in the reaction mixture. Performing the reaction with deuterated cyclohexanol instead would also not give a meaningful result as in this case H/D exchange has to be expected and, hence, the measured values would also be misleading. As a control experiment, we subjected only the deuterated starting material **19** to the reaction conditions (Scheme 8). This experiment delivered exclusively the H-containing product. This proves that KIE studies are not possible for the aryl chloride protocol.

## Conclusions

Acyclic sp^3^ C–H bonds adjacent to a free N–H group were readily arylated by cyclometalation by employing [RuCl_2_(*p*-cymene)]_2_ and carboxylates with aryl bromides and iodides. Improvements in the conversion in the presence of carboxylates can be explained by a CMD mechanism. Furthermore, the protocol was expanded to cheaper aryl chlorides by using phosphanes as ligands and secondary alcohols as the hydrogen source. The synthetic utility of this approach was demonstrated by the synthesis of various arylated benzylic amines. A wide range of substituents were used in the reaction, and moderate-to-good yields were achieved. The electronic nature of the substituents affects the electron density of the benzylic C–H bond, which has a significant impact on the C–H functionalization rate. Electron-withdrawing and coordinating substituents inhibited the reaction. A free N–H group was mandatory for the arylation, which indicates that imine formation is a crucial step in this reaction. KIE experiments of the Ru^II^ protocol revealed that the oxidative addition step is not the rate-determining step for aryl bromides and aryl iodides. For the aryl chloride protocol, no KIE measurements could be undertaken owing to a competing H–D exchange. The establishment of these conditions should provide a valuable starting point for subsequent examinations of direct arylation in C–C bond synthesis and may facilitate the discovery of other new cross-coupling partners in this type of chemistry.

## Experimental Section

**General Methods:** All reactions were carried out under argon, unless otherwise mentioned. Argon was purified by passage through Drierite. Unless otherwise noted, chemicals were purchased from commercial suppliers and were used without further purification. HRMS for literature unknown compounds were analyzed by hybrid ion trap/time-of-flight MS coupled with liquid chromatography (LC-IT-TOF-MS) in positive ion detection mode with the recording of MS and MS/MS spectra. NMR spectra were recorded in CDCl_3_ with TMS as internal standard and chemical shifts are reported in ppm. GC–MS runs were performed with a standard capillary column (BGB 5, 30 m × 0.32 mm i.d.). Microwave reactions were performed with a BIOTAGE Initiator sixty microwave unit (max pressure 20 bar, IR temperature sensor). Analytical data for all new compounds are given below. Compounds **12**^18^ and **19**^6^ were prepared according to the literature procedures.

**General Procedure I for the Preparation of Benzylic Amines:** The 2-choloro-3-substituted pyridine (1 equiv.), amine (1.2 equiv.), K_2_CO_3_ (3.5 equiv.), Pd(OAc)_2_ (2 mol-%), and BINAP (2 mol-%) were placed in an oven-dried 6 mL vial with septum screw cap and a magnetic stirring bar. The vial was evacuated and flushed with argon (three times). Dry toluene was added to the reaction mixture, and the vial was closed with a fully covered solid Teflon®-lined cap. The reaction vial was then heated in a reaction block at 130 °C for 16 h. The suspension was cooled to room temp., and the solid material was removed by filtration and washed with CH_2_Cl_2_ (10 mL). The combined organic layers were evaporated, and the resulting crude product was purified by flash column chromatography (PE/EtOAc = 10:1).

**General Procedure II for the Preparation of Tertiary Amines:** The 2-bromo-3-substituted pyridine (1 equiv.), amine (1.4 equiv.), NaO*t*Bu (2 equiv.), bis(dibenzylideneacetone)palladium [Pd_2_(dba)_2_, 2 mol-%], and DPPP [1,3-bis(diphenylphosphanyl)propane, 2 mol-%] were placed in an oven-dried 6 mL vial with a septum screw cap and a magnetic stirring bar. The vial was evacuated and flushed with argon (three times). Dry toluene was added to the reaction mixture, and the vial was closed with a fully covered solid Teflon®-lined cap. The reaction vial was then heated in a reaction block at 75 °C for 16 h. The suspension was cooled to room temp., and the solid material was removed by filtration and washed with CH_2_Cl_2_ (10 mL). The combined organic layers were evaporated, and the resulting crude product was purified by flash column chromatography (PE/EtOAc = 15:1/10:1).

**General Procedure III for the C–H Activation Reaction with Aryl Bromides:** [RuCl_2_(*p*-cymene)]_2_ (2.5 mol-%) and KOPiv (30 mol-%) were placed in an oven-dried 6 mL vial with a septum screw cap and a magnetic stirring bar. The vial was evacuated and flushed with argon (three times). Dry toluene (2 mL) was added, and the reaction mixture was stirred at room temp. for 30 min. Subsequently, the pyridine derivative (0.5 mmol, 1 equiv.), aryl bromide (0.75 mmol, 1.5 equiv.), and K_2_CO_3_ (1.5 mmol, 3 equiv.) were added to the mixture. The vial was again evacuated, flushed with argon, closed with a fully covered solid Teflon®-lined cap, and heated in a reaction block at 140–150 °C for 24 h. The suspension was cooled to room temp. and filtered through a short pad of Celite, which was further washed with CH_2_Cl_2_ (2 × 5 mL). The combined organic layers were concentrated in vacuo, and the remaining residue was purified by flash column chromatography (PE/EtOAc = 49:1) and dried under high vacuum. Compounds **3a**–**3ah** and **4** were prepared according to this procedure.

**General Procedure IV for the C–H Activation Reaction with Aryl Chlorides:** [RuCl_2_(*p*-cymene)]_2_ (0.025 mmol, 5 mol-%) and PPh_3_ (0.05 mmol, 10 mol-%) were placed in an oven-dried 6 mL vial with a septum screw cap and a magnetic stirring bar. The vial was evacuated and flushed with argon (3 ×). Dry *o*-xylene (2 mL) was added, and the reaction mixture was stirred at room temp. for 30 min. Subsequently, the pyridine derivative (0.5 mmol, 1 equiv.), aryl chloride (1.5 mmol, 3 equiv.), cyclohexanol (0.5 mmol, 1 equiv.), and K_2_CO_3_ (1.5 mmol, 3 equiv.) were added to the mixture. The vial was again evacuated and flushed with argon, closed with a fully covered solid Teflon®-lined cap, and heated in a reaction block at 160 °C for 30 h. The suspension was cooled to room temp. and then filtered through a short pad of Celite, which was further washed with DCM (2 × 5 mL). The combined organic layers were concentrated in vacuo, and the remaining residue was purified by flash column chromatography (PE/EtOAc = 49:1) and dried under high vacuum.

***N*-Benzyl-3-methylpyridin-2-amine (1a):**^19^ The reaction was carried out according to general procedure I with 2-chloro-3-methylpyridine (128 mg, 1 mmol, 1 equiv.), benzylamine (128 mg, 1.2 mmol, 1.2 equiv.), K_2_CO_3_ (483 mg, 3.5 mmol, 3.5 equiv.), Pd(OAc)_2_ (4 mg, 0.02 mmol, 2 mol-%), and BINAP (12 mg, 0.02 mmol, 2 mol-%) in dry toluene (2.5 mL). Colorless solid (182 mg, 92 % yield); m.p. 48–49 °C. ^1^H NMR (CDCl_3_, 200 MHz): *δ* = 2.09 (s, 3 H), 4.36 (s, 1 H), 4.70 (d, *J* = 5.3 Hz, 2 H), 6.57 (dd, *J* = 7.1, 5.1 Hz, 1 H), 7.23–7.43 (m, 6 H), 8.06 (dd, *J* = 5.0, 1.3 Hz, 1 H) ppm. ^13^C NMR (CDCl_3_, 50 MHz): *δ* = 17.1, 45.9, 113.0, 116.6, 127.3, 128.0, 128.7, 136.9, 140.1, 145.6, 156.8 ppm.

***N*-Benzyl-3-chloropyridin-2-amine:**^20^ The reaction was carried out according to general procedure I with 2,3-dichloropyridine (148 mg, 1 mmol, 1 equiv.), benzylamine (128 mg, 1.2 mmol, 1.2 equiv.), K_2_CO_3_ (483 mg, 3.5 mmol, 3.5 equiv.), Pd(OAc)_2_ (4 mg, 0.02 mmol, 2 mol-%), and BINAP (12 mg, 0.02 mmol, 2 mol-%) in dry toluene (2.5 mL). Yellow oil (200 mg, 91 % yield). ^1^H NMR (CDCl_3_, 200 MHz): *δ* = 4.68 (d, *J* = 5.6 Hz, 2 H), 5.26 (s, 1 H), 6.54 (dd, *J* = 7.6, 4.9 Hz, 1 H), 7.23–7.39 (m, 5 H), 7.45 (dd, *J* = 7.6, 1.6 Hz, 1 H) 8.04 (dd, *J* = 4.9, 1.6 Hz, 1 H) ppm. ^13^C NMR (CDCl_3_, 50 MHz): *δ* = 45.6, 113.2, 115.4, 127.4, 127.8, 128.7, 136.2, 139.4, 146.2, 154.0 ppm.

***N*-Benzyl-3-phenylpyridin-2-amine (1b):**^21^ The reaction was carried out according to general procedure I with *N*-benzyl-3-chloropyridin-2-amine obtained from the above protocol (219 mg, 1 mmol, 1 equiv.), phenylboronic acid (366 mg, 3 mmol, 3 equiv.), K_2_CO_3_ (276 mg, 2 mmol, 2 equiv.), Pd(OAc)_2_ (4 mg, 0.02 mmol, 2 mol-%), and 2-dicyclohexylphosphanyl-2′,4′,6′-triisopropylbiphenyl (DCPTPB, 10 mg, 0.02 mmol, 2 mol-%) in dry toluene (2.5 mL). Colorless solid (255 mg, 98 % yield); m.p. 58–60 °C. ^1^H NMR (CDCl_3_, 200 MHz): *δ* = 4.64 (d, *J* = 5.6 Hz, 2 H), 4.88 (s, 1 H), 6.66 (dd, *J* = 7.2, 5.1 Hz, 1 H), 7.18–7.42 (m, 11 H), 8.14 (dd, *J* = 4.9, 1.5 Hz, 1 H) ppm. ^13^C NMR (CDCl_3_, 50 MHz): *δ* = 45.6, 113.1, 122.4, 127.1, 127.5, 127.9, 128.6, 129.0, 129.3, 137.2, 138.0, 140.0, 147.2, 155.5 ppm. HRMS: calcd. for C_18_H_16_N_2_ [M + H]^+^ 261.1386; found 261.1390.

***N*-(4-Methoxybenzyl)-3-methylpyridin-2-amine (1c):**^21^ The reaction was carried out according to general procedure I with 2-chloro-3-methylpyridine (128 mg, 1 mmol, 1 equiv.), 4-methoxybenzylamine (164 mg, 1.2 mmol, 1.2 equiv.), K_2_CO_3_ (414 mg, 3 mmol, 3 equiv.), Pd(OAc)_2_ (4 mg, 0.02 mmol, 2 mol-%), and BINAP (12 mg, 0.02 mmol, 2 mol-%) in dry toluene (2.5 mL). Yellow oil (183 mg, 80 % yield). ^1^H NMR (CDCl_3_, 200 MHz): *δ* = 2.07 (s, 3 H), 3.80 (s, 3 H), 4.30 (s, 1 H), 4.61 (d, *J* = 5.2 Hz, 2 H), 6.55 (dd, *J* = 7.1, 5.1 Hz, 1 H), 6.88 (d, *J* = 8.6 Hz, 2 H), 7.21–7.34 (m, 3 H), 8.06 (dd, *J* = 4.9, 1.0 Hz, 1 H) ppm. ^13^C NMR (CDCl_3_, 50 MHz): *δ* = 17.1, 45.4, 55.4, 112.9, 114.1, 116.6, 129.3, 132.1, 136.9, 145.5, 156.8, 158.9 ppm. HRMS: calcd. for C_14_H_16_N_2_O [M + H]^+^ 229.1335; found 229.1338.

***N*-(4-Isopropoxybenzyl)-3-methylpyridin-2-amine (1d):**^21^ The reaction was carried out according to general procedure I with 2-chloro-3-methylpyridine (128 mg, 1 mmol, 1 equiv.), (4-isopropoxyphenyl)methanamine (198 mg, 1.2 mmol, 1.2 equiv.), K_2_CO_3_ (483 mg, 3.5 mmol, 3.5 equiv.), Pd(OAc)_2_ (4 mg, 0.02 mmol, 2 mol-%), and BINAP (12 mg, 0.02 mmol, 2 mol-%) in dry toluene (2.5 mL). Colorless oil (185 mg, 72 % yield). ^1^H NMR (CDCl_3_, 200 MHz): *δ* = 1.33 (d, *J* = 6.0 Hz, 6 H), 2.06 (s, 3 H), 4.27 (s, 1 H), 4.51 (sep, *J* = 6.0 Hz, 1 H), 4.59 (d, *J* = 5.2 Hz, 2 H), 6.54 (dd, *J* = 7.1, 5.1 Hz, 1 H), 6.86 (d, *J* = 8.6 Hz, 2 H), 7.20–7.32 (m, 3 H), 8.05 (dd, *J* = 5.1, 1.3 Hz, 1 H) ppm. ^13^C NMR (CDCl_3_, 50 MHz): *δ* = 17.1, 21.2, 45.5, 70.0, 112.9, 116.0, 116.6, 129.3, 131.9, 136.9, 145.6, 156.8, 157.3 ppm. HRMS: calcd. for C_16_H_20_N_2_O [M + H]^+^ 257.1648; found 257.1642.

**3-Methyl-*N*-(4-methylbenzyl)pyridin-2-amine (1e):**^21^ The reaction was carried out according to general procedure I with 2-chloro-3-methylpyridine (128 mg, 1 mmol, 1 equiv.), 4-methylbenzylamine (145 mg, 1.2 mmol, 1.2 equiv.), K_2_CO_3_ (414 mg, 3 mmol, 3 equiv.), Pd(OAc)_2_ (4 mg, 0.02 mmol, 2 mol-%), and BINAP (12 mg, 0.02 mmol, 2 mol-%) in dry toluene (2.5 mL). Colorless solid (188 mg, 88 % yield); m.p. 46–47 °C. ^1^H NMR (CDCl_3_, 200 MHz): *δ* = 2.01 (s, 3 H), 2.30 (s, 3 H), 4.26 (s, 1 H), 4.59 (d, *J* = 5.2 Hz, 2 H), 6.50 (dd, *J* = 7.1, 5.1 Hz, 1 H), 7.08–7.26 (m, 5 H), 8.00 (dd, *J* = 5.0, 1.3 Hz, 1 H) ppm. ^13^C NMR (CDCl_3_, 50 MHz): *δ* = 17.1, 21.2, 45.8, 112.9, 116.6, 128.0, 129.4, 136.8, 136.9, 137.0, 145.5, 156.8 ppm. HRMS: calcd. for C_14_H_16_N_2_ [M + H]^+^ 213.1386; found 213.1380.

***N*-(4-Fluorobenzyl)-3-methylpyridin-2-amine (1f):**^21^ The reaction was carried out according to general procedure I with 2-chloro-3-methylpyridine (128 mg, 1 mmol, 1 equiv.), (4-fluorophenyl)methanamine (150 mg, 1.2 mmol, 1.2 equiv.), K_2_CO_3_ (483 mg, 3.5 mmol, 3.5 equiv.), Pd(OAc)_2_ (4 mg, 0.02 mmol, 2 mol-%), and BINAP (12 mg, 0.02 mmol, 2 mol-%) in dry toluene (2.5 mL). Colorless oil (158 mg, 73 % yield). ^1^H NMR (CDCl_3_, 200 MHz): *δ* = 2.08 (s, 3 H), 4.36 (s, 1 H), 4.65 (d, *J* = 5.4 Hz, 2 H), 6.56 (dd, *J* = 7.1, 5.1 Hz, 1 H), 6.96–7.05 (m, 2 H), 7.21–7.37 (m, 3 H), 8.03 (dd, *J* = 5.0, 1.2 Hz, 1 H) ppm. ^13^C NMR (CDCl_3_, 50 MHz): *δ* = 17.1, 45.1, 113.2, 115.5 (d, *J*_C,F_ = 21.3 Hz), 116.6, 129.5 (d, *J*_C,F_ = 8.0 Hz), 135.9 (d, *J*_C,F_ = 3.1 Hz), 137.1, 145.6, 156.6, 162.2 (d, *J*_C,F_ = 244.9 Hz) ppm. HRMS: calcd. for C_13_H_13_FN_2_ [M + H]^+^ 217.1136; found 217.1128.

**3-Methyl-*N*-[4-(trifluoromethyl)benzyl]pyridin-2-amine (1g):**^21^ The reaction was carried out according to general procedure I with 2-chloro-3-methylpyridine (128 mg, 1 mmol, 1 equiv.), [4-(trifluoromethyl)phenyl]methanamine (210 mg, 1.2 mmol, 1.2 equiv.), K_2_CO_3_ (483 mg, 3.5 mmol, 3.5 equiv.), Pd(OAc)_2_ (4 mg, 0.02 mmol, 2 mol-%), and BINAP (12 mg, 0.02 mmol, 2 mol-%) in dry toluene (2.5 mL). Colorless solid (195 mg, 73 % yield); m.p. 54–55 °C. ^1^H NMR (CDCl_3_, 200 MHz): *δ* = 2.14 (s, 3 H), 4.50 (s, 1 H), 4.78 (d, *J* = 5.7 Hz, 2 H), 6.58 (dd, *J* = 7.1, 5.1 Hz, 1 H), 7.25–7.29 (m, 1 H), 7.53 (d, *J* = 9.7 Hz, 4 H), 8.03 (dd, *J* = 5.0, 1.2 Hz, 1 H) ppm. ^13^C NMR (CDCl_3_, 50 MHz): *δ* = 17.1, 45.2, 113.5, 116.7, 124.4 (q, *J*_C,F_ = 271.9 Hz), 125.6 (q, *J*_C,F_ = 3.9 Hz), 127.9, 129.4 (q, *J*_C,F_ = 32.3 Hz), 137.2, 144.6, 145.6, 156.4 ppm. HRMS: calcd. for C_14_H_13_F_3_N_2_ [M + H]^+^ 267.1104; found 267.1099.

**Methyl 4-{[(3-Methylpyridin-2-yl)amino]methyl}benzoate (1h):**^21^ The reaction was carried out according to general procedure I with 2-chloro-3-methylpyridine (128 mg, 1 mmol, 1 equiv.), methyl 4-(aminomethyl)benzoate (198 mg, 1.2 mmol, 1.2 equiv.), K_2_CO_3_ (414 mg, 3 mmol, 3 equiv.), Pd(OAc)_2_ (4 mg, 0.02 mmol, 2 mol-%), and BINAP (12 mg, 0.02 mmol, 2 mol-%) in dry toluene (2.5 mL). Colorless solid (223 mg, 87 % yield); m.p. 122–123 °C. ^1^H NMR (CDCl_3_, 200 MHz): *δ* = 2.12 (s, 3 H), 3.90 (s, 3 H), 4.48 (s, 1 H), 4.77 (d, *J* = 5.7 Hz, 2 H), 6.56 (dd, *J* = 7.1, 5.1 Hz, 1 H), 7.23–7.27 (m, 1 H), 7.43 (d, *J* = 8.2 Hz, 2 H), 7.97–8.02 (m, 3 H) ppm. ^13^C NMR (CDCl_3_, 50 MHz): *δ* = 17.1, 45.3, 52.2, 113.3, 116.6, 127.5, 129.0, 130.0, 137.1, 145.6, 145.8, 156.5, 167.1 ppm. HRMS: calcd. for C_15_H_16_N_2_O_2_ [M + H]^+^ 257.1285; found 257.1296.

***N*-Benzhydryl-3-methylpyridin-2-amine (3a):**^12^ The reaction was carried out according to general procedure III with **1a** (99 mg, 0.5 mmol, 1 equiv.), bromobenzene (118 mg, 0.75 mmol, 1.5 equiv.), [RuCl_2_(*p*-cymene)]_2_ (7.6 mg, 0.0125 mmol, 2.5 mol-%), KOPiv (21 mg, 0.15 mmol, 30 mol-%), and K_2_CO_3_ (207 mg, 1.5 mmol, 3 equiv.) in dry toluene (2 mL). Colorless solid (95 mg, 69 % yield); m.p. 91–93 °C. ^1^H NMR (CDCl_3_, 200 MHz): *δ* = 2.07 (s, 3 H), 4.60 (d, *J* = 6.8 Hz, 1 H), 6.42–6.48 (m, 2 H), 7.12–7.29 (m, 11 H), 7.89 (dd, *J* = 5.0, 1.3 Hz, 1 H) ppm. ^13^C NMR (CDCl_3_, 50 MHz): *δ* = 17.2, 58.6, 113.2, 116.4, 127.1, 127.7, 128.6, 137.0, 143.6, 145.7, 155.8 ppm.

**3-Methyl-*N*-[phenyl(m-tolyl)methyl]pyridin-2-amine (3d):**^21^ The reaction was carried out according to general procedure III with **1a** (99 mg, 0.5 mmol, 1 equiv.), 1-bromo-3-methylbenzene (128 mg, 0.75 mmol, 1.5 equiv.), [RuCl_2_(*p*-cymene)]_2_ (7.6 mg, 0.0125 mmol, 2.5 mol-%), KOPiv (21 mg, 0.15 mmol, 30 mol-%), and K_2_CO_3_ (207 mg, 1.5 mmol, 3 equiv.) in dry toluene (2 mL). Colorless oil (79 mg, 55 % yield). ^1^H NMR (CDCl_3_, 200 MHz): *δ* = 2.11 (s, 3 H), 2.29 (s, 3 H), 4.63 (d, *J* = 7.2 Hz, 1 H), 6.46–6.52 (m, 2 H), 7.01–7.34 (m, 10 H), 7.95 (dd, *J* = 5.0, 1.2 Hz, 1 H) ppm. ^13^C NMR (CDCl_3_, 50 MHz): *δ* = 17.2, 21.6, 58.5, 113.1, 116.4, 124.7, 127.0, 127.6, 127.9, 128.5, 128.6, 129.3, 137.0, 138.2, 143.6, 143.7, 145.8, 155.8 ppm. HRMS: calcd. for C_20_H_20_N_2_ [M + H]^+^ 289.1699; found 289.1679.

***N*-[(3-Methoxyphenyl)(phenyl)methyl]-3-methylpyridin-2-amine (3e):** The reaction was carried out according to general procedure III with **1a** (99 mg, 0.5 mmol, 1 equiv.), 1-bromo-3-methoxybenzene (140 mg, 0.75 mmol, 1.5 equiv.), [RuCl_2_(*p*-cymene)]_2_ (7.6 mg, 0.0125 mmol, 2.5 mol-%) and KOPiv (21 mg, 0.15 mmol, 30 mol-%) in dry toluene (2 mL). Colorless oil (91 mg, 60 % yield). ^1^H NMR (CDCl_3_, 200 MHz): *δ* = 2.12 (s, 3 H), 3.73 (s, 3 H), 4.65 (d, *J* = 7.5 Hz, 1 H), 6.48–6.54 (m, 2 H), 6.74–6.80 (m, 1 H), 6.89–6.93 (m, 2 H), 7.18–7.37 (m, 7 H), 7.96 (dd, *J* = 5.0, 1.2 Hz, 1 H) ppm. ^13^C NMR (CDCl_3_, 50 MHz): *δ* = 17.1, 55.2, 58.5, 112.1, 113.2, 113.7, 116.4, 120.0, 127.1, 127.6, 128.6, 129.6, 137.0, 143.5, 145.3, 145.7, 155.7, 159.8 ppm. HRMS: calcd. for C_20_H_20_N_2_O [M + H]^+^ 305.1648; found 305.1637.

***N*-[(3-Chlorophenyl)(phenyl)methyl]-3-methylpyridin-2-amine (3f):**^21^ The reaction was carried out according to general procedure III with **1a** (99 mg, 0.5 mmol, 1 equiv.), 1-bromo-3-chlorobenzene (143 mg, 0.75 mmol, 1.5 equiv.), [RuCl_2_(*p*-cymene)]_2_ (7.6 mg, 0.0125 mmol, 2.5 mol-%), KOPiv (21 mg, 0.15 mmol, 30 mol-%), and K_2_CO_3_ (207 mg, 1.5 mmol, 3 equiv.) in dry toluene (2 mL). Colorless oil (58 mg, 37 % yield). ^1^H NMR (CDCl_3_, 200 MHz): *δ* = 2.13 (s, 3 H), 4.59 (d, *J* = 6.6 Hz, 1 H), 6.46–6.57 (m, 2 H), 7.19–7.32 (m, 10 H), 7.95 (dd, *J* = 5.0, 1.3 Hz, 1 H) ppm. ^13^C NMR (CDCl_3_, 50 MHz): *δ* = 17.2, 58.3, 113.6, 116.6, 125.8, 127.3, 127.5, 127.6, 127.8, 128.9, 129.8, 134.5, 137.2, 143.0, 145.7, 155.6 ppm. HRMS: calcd. for C_19_H_17_ClN_2_ [M + H]^+^ 309.1153; found 309.1138.

**3-Methyl-*N*-[phenyl(p-tolyl)methyl]pyridin-2-amine (3g):**^21^ The reaction was carried out according to general procedure III with **1a** (99 mg, 0.5 mmol, 1 equiv.), 1-bromo-4-methylbenzene (128 mg, 0.75 mmol, 1.5 equiv.), [RuCl_2_(*p*-cymene)]_2_ (7.6 mg, 0.0125 mmol, 2.5 mol-%), KOPiv (21 mg, 0.15 mmol, 30 mol-%), and K_2_CO_3_ (207 mg, 1.5 mmol, 3 equiv.) in dry toluene (2 mL). Colorless solid (94 mg, 65 % yield); m.p. 103–105 °C. ^1^H NMR (CDCl_3_, 200 MHz): *δ* = 2.13 (s, 3 H), 2.32 (s, 3 H), 4.64 (d, *J* = 6.8 Hz, 1 H), 6.46–6.54 (m, 2 H), 7.09–7.32 (m, 10 H), 7.96 (dd, *J* = 5.0, 1.3 Hz, 1 H) ppm. ^13^C NMR (CDCl_3_, 50 MHz): *δ* = 17.2, 21.2, 58.3, 113.1, 116.4, 127.0, 127.6, 127.7, 128.6, 129.3, 136.8, 137.0, 140.7, 143.7, 145.8, 155.8 ppm. HRMS: calcd. for C_20_H_20_N_2_ [M + H]^+^ 289.1699; found 289.1699.

***N*-{[4-(*tert*-Butyl)phenyl](phenyl)methyl}-3-methylpyridin-2-amine (3h):**^21^ The reaction was carried out according to general procedure III with **1a** (99 mg, 0.5 mmol, 1 equiv.), 1-bromo-4-(*tert*-butyl)benzene (160 mg, 0.75 mmol, 1.5 equiv.), [RuCl_2_(*p*-cymene)]_2_ (7.6 mg, 0.0125 mmol, 2.5 mol-%), KOPiv (21 mg, 0.15 mmol, 30 mol-%), and K_2_CO_3_ (207 mg, 1.5 mmol, 3 equiv.) in dry toluene (2 mL). Colorless solid (106 mg, 64 % yield); m.p. 120–122 °C. ^1^H NMR (CDCl_3_, 200 MHz): *δ* = 1.29 (s, 9 H), 2.13 (s, 3 H), 4.66 (d, *J* = 6.8 Hz, 1 H), 6.48–6.53 (m, 2 H), 7.20–7.34 (m, 10 H), 7.96 (dd, *J* = 5.0, 1.3 Hz, 1 H) ppm. ^13^C NMR (CDCl_3_, 50 MHz): *δ* = 17.3, 31.5, 34.6, 58.2, 113.1, 116.4, 125.6, 127.0, 127.4, 127.6, 128.5, 137.0, 140.6, 143.7, 145.8, 150.0, 155.9 ppm. HRMS: calcd. for C_23_H_26_N_2_ [M + H]^+^ 331.2169; found 331.2178.

***N*-[(4-Butylphenyl)(phenyl)methyl]-3-methylpyridin-2-amine (3i):** The reaction was carried out according to general procedure III with **1a** (99 mg, 0.5 mmol, 1 equiv.), 1-bromo-4-butylbenzene (160 mg, 0.75 mmol, 1.5 equiv.), [RuCl_2_(*p*-cymene)]_2_ (7.6 mg, 0.0125 mmol, 2.5 mol-%), and KOPiv (21 mg, 0.15 mmol, 30 mol-%) in dry toluene (2 mL). Colorless oil (111 mg, 67 % yield). ^1^H NMR (CDCl_3_, 200 MHz): *δ* = 0.89 (t, *J* = 7.2 Hz, 3 H), 1.23–1.63 (m, 2 H), 1.48–1.63 (m, 2 H), 2.08 (s, 3 H), 2.55 (t, *J* = 7.7 Hz, 2 H), 4.62 (d, *J* = 7.0 Hz, 1 H), 6.43–6.50 (m, 2 H), 7.06–7.33 (m, 10 H), 7.93 (dd, *J* = 5.0, 1.3 Hz, 1 H) ppm. ^13^C NMR (CDCl_3_, 50 MHz): *δ* = 14.1, 17.2, 22.5, 33.7, 35.4, 58.3, 113.1, 116.4, 127.0, 127.5, 127.6, 128.5, 128.6, 137.0, 140.9, 141.7, 143.8, 145.8, 155.9 ppm. HRMS: calcd. for C_23_H_26_N_2_ [M + H]^+^ 331.2169; found 331.2156.

***N*-[(4-Methoxyphenyl)(phenyl)methyl]-3-methylpyridin-2-amine (3j):**^21^ The reaction was carried out according to general procedure III with **1a** (99 mg, 0.5 mmol, 1 equiv.), 1-bromo-4-methoxybenzene (140 mg, 0.75 mmol, 1.5 equiv.), [RuCl_2_(*p*-cymene)]_2_ (7.6 mg, 0.0125 mmol, 2.5 mol-%), and KOPiv (21 mg, 0.15 mmol, 30 mol-%) in dry toluene (2 mL). Colorless solid (96 mg, 63 % yield); m.p. 59–61 °C. ^1^H NMR (CDCl_3_, 200 MHz): *δ* = 2.14 (s, 3 H), 3.79 (s, 3 H), 4.63 (d, *J* = 6.5 Hz, 1 H), 6.46–6.55 (m, 2 H), 6.82–6.89 (m, 2 H), 7.22–7.36 (m, 8 H), 7.97 (dd, *J* = 5.0, 1.3 Hz, 1 H) ppm. ^13^C NMR (CDCl_3_, 50 MHz): *δ* = 17.2, 55.4, 58.0, 113.2, 114.0, 116.4, 127.0, 127.6, 128.6, 128.9, 135.8, 137.0, 143.8, 145.8, 155.8, 158.7 ppm. HRMS: calcd. for C_20_H_20_N_2_O [M + H]^+^ 305.1648; found 305.1655.

***N*-{[4-(Dimethylamino)phenyl](phenyl)methyl}-3-methylpyridin-2-amine (3k):** The reaction was carried out according to general procedure III with **1a** (99 mg, 0.5 mmol, 1 equiv.), 4-bromo-*N*,*N*-dimethylaniline (150 mg, 0.75 mmol, 1.5 equiv.), [RuCl_2_(*p*-cymene)]_2_ (7.6 mg, 0.0125 mmol, 2.5 mol-%), and KOPiv (21 mg, 0.15 mmol, 30 mol-%) in dry toluene (2 mL). Yellow oil (79 mg, 50 % yield). ^1^H NMR (CDCl_3_, 200 MHz): *δ* = 2.10 (s, 3 H), 2.90 (s, 6 H), 4.62 (d, *J* = 6.8 Hz, 1 H), 6.41–6.51 (m, 2 H), 6.64–6.69 (m, 2 H), 7.13–7.35 (m, 8 H), 7.95 (dd, *J* = 5.0, 1.3 Hz, 1 H) ppm. ^13^C NMR (CDCl_3_, 50 MHz): *δ* = 17.2, 40.7, 58.0, 112.7, 112.9, 116.4, 126.7, 127.4, 128.4, 128.7, 131.6, 136.9, 144.0, 145.7, 149.8, 155.9 ppm. HRMS: calcd. for C_21_H_23_N_3_ [M + H]^+^ 318.1965; found 318.1955.

***N*-[(4-Fluorophenyl)(phenyl)methyl]-3-methylpyridin-2-amine (3l):**^21^ The reaction was carried out according to general procedure III with **1a** (99 mg, 0.5 mmol, 1 equiv.), 1-bromo-4-fluorobenzene (131 mg, 0.75 mmol, 1.5 equiv.), [RuCl_2_(*p*-cymene)]_2_ (7.6 mg, 0.0125 mmol, 2.5 mol-%), and KOPiv (21 mg, 0.15 mmol, 30 mol-%) in dry toluene (2 mL). Colorless solid (89 mg, 61 % yield); m.p. 101–103 °C. ^1^H NMR (CDCl_3_, 200 MHz): *δ* = 2.07 (s, 3 H), 4.55 (d, *J* = 6.7 Hz, 1 H), 6.42–6.50 (m, 2 H), 6.86–6.97 (m, 2 H), 7.16–7.26 (m, 8 H), 7.90 (dd, *J* = 5.0, 1.3 Hz, 1 H) ppm. ^13^C NMR (CDCl_3_, 50 MHz): *δ* = 17.2, 58.0, 113.4, 115.4 (d, *J* = 21.3 Hz), 116.5, 127.3, 127.7, 128.7, 129.2 (d, *J* = 8.1 Hz), 137.2, 139.3 (d, *J* = 3.1 Hz), 143.4, 145.7, 155.6, 161.9 (d, *J* = 245.0 Hz) ppm. HRMS: calcd. for C_19_H_17_N_2_F [M + H]^+^ 293.1449; found 293.1448.

***N*-[(4-Chlorophenyl)(phenyl)methyl]-3-methylpyridin-2-amine(3m):**^21^ The reaction was carried out according to general procedure III with **1a** (99 mg, 0.5 mmol, 1 equiv.), 1-bromo-4-chlorobenzene (143 mg, 0.75 mmol, 1.5 equiv.), [RuCl_2_(*p*-cymene)]_2_ (7.6 mg, 0.0125 mmol, 2.5 mol-%), and KOPiv (21 mg, 0.15 mmol, 30 mol-%) in dry toluene (2 mL). Colorless oil (79 mg, 51 % yield). ^1^H NMR (CDCl_3_, 200 MHz): *δ* = 2.12 (s, 3 H), 4.59 (d, *J* = 6.6 Hz, 1 H), 6.45–6.55 (m, 2 H), 7.21–7.32 (m, 10 H), 7.94 (dd, *J* = 5.0, 1.3 Hz, 1 H) ppm. ^13^C NMR (CDCl_3_, 50 MHz): *δ* = 17.1, 58.2, 113.5, 116.5, 127.5, 127.8, 128.7, 128.8, 129.0, 132.7, 137.1, 142.1, 143.2, 145.7, 155.6 ppm. HRMS: calcd. for C_19_H_17_ClN_2_ [M + H]^+^ 309.1153; found 309.1138.

**Ethyl 4-{[(3-Methylpyridin-2-yl)amino](phenyl)methyl}benzoate (3n):** The reaction was carried out according to general procedure III with **1a** (99 mg, 0.5 mmol, 1 equiv.), ethyl 4-bromobenzoate (172 mg, 0.75 mmol, 1.5 equiv.), [RuCl_2_(*p*-cymene)]_2_ (7.6 mg, 0.0125 mmol, 2.5 mol-%), and KOPiv (21 mg, 0.15 mmol, 30 mol-%) in dry toluene (2 mL). Colorless oil (57 mg, 33 % yield). ^1^H NMR (CDCl_3_, 200 MHz): *δ* = 1.37 (t, *J* = 7.1 Hz, 3 H), 2.15 (s, 3 H), 4.36 (q, *J* = 7.1 Hz, 2 H), 4.66 (d, *J* = 6.6 Hz, 1 H), 6.51–6.57 (m, 2 H), 7.23–7.35 (m, 6 H), 7.42 (d, *J* = 8.1 Hz, 2 H), 7.94–8.02 (m, 3 H) ppm. ^13^C NMR (CDCl_3_, 50 MHz): *δ* = 14.4, 17.1, 58.7, 60.9, 113.5, 116.6, 127.4, 127.6, 127.9, 128.8, 129.3, 129.9, 137.1, 143.0, 145.7, 148.7, 155.6, 166.6 ppm. HRMS: calcd. for C_22_H_22_N_2_O_2_ [M + H]^+^ 347.1754; found 347.1737.

***N*-Benzhydryl-3-phenylpyridin-2-amine (3t):**^21^ The reaction was carried out according to general procedure III with **1b** (130 mg, 0.5 mmol, 1 equiv.), bromobenzene (118 mg, 0.75 mmol, 1.5 equiv.), [RuCl_2_(*p*-cymene)]_2_ (7.6 mg, 0.0125 mmol, 2.5 mol-%), KOPiv (21 mg, 0.15 mmol, 30 mol-%), and K_2_CO_3_ (207 mg, 1.5 mmol, 3 equiv.) in dry toluene (2 mL). Colorless solid (118 mg, 70 % yield); m.p. 90–92 °C. ^1^H NMR (CDCl_3_, 200 MHz): *δ* = 5.18 (d, *J* = 7.4 Hz, 1 H), 6.51 (d, *J* = 7.5 Hz, 1 H), 6.64 (dd, *J* = 7.2, 5.0 Hz, 1 H), 7.14–7.44 (m, 16 H), 8.08 (dd, *J* = 5.0, 1.8 Hz, 1 H) ppm. ^13^C NMR (CDCl_3_, 50 MHz): *δ* = 58.5, 113.5, 122.4, 127.1, 127.5, 128.0, 128.6, 128.9, 129.4, 137.4, 138.1, 143.5, 147.4, 154.6 ppm. HRMS: calcd. for C_24_H_20_N_2_ [M + H]^+^ 337.1699; found 337.1713.

**3-Phenyl-*N*-[phenyl(m-tolyl)methyl]pyridin-2-amine (3u):** The reaction was carried out according to general procedure III with **1b** (130 mg, 0.5 mmol, 1 equiv.), 1-bromo-3-methylbenzene (128 mg, 0.75 mmol, 1.5 equiv.), [RuCl_2_(*p*-cymene)]_2_ (7.6 mg, 0.0125 mmol, 2.5 mol-%), KOPiv (21 mg, 0.15 mmol, 30 mol-%), and K_2_CO_3_ (207 mg, 1.5 mmol, 3 equiv.) in dry toluene (2 mL). Colorless oil (119 mg, 68 % yield). ^1^H NMR (CDCl_3_, 200 MHz): *δ* = 2.25 (s, 3 H), 5.18 (d, *J* = 7.5 Hz, 1 H), 6.49 (d, *J* = 7.5 Hz, 1 H), 6.61 (dd, *J* = 7.2, 5.0 Hz, 1 H), 6.97–7.42 (m, 15 H), 8.08 (dd, *J* = 5.0, 1.8 Hz, 1 H) ppm. ^13^C NMR (CDCl_3_, 50 MHz): *δ* = 21.6, 58.6, 113.3, 122.3, 124.5, 127.0, 127.5, 127.8, 127.9, 128.3, 128.4, 128.5, 128.9, 129.3, 137.3, 138.0, 138.1, 143.4, 143.6, 147.4, 154.6 ppm. HRMS: calcd. for C_25_H_22_N_2_ [M + H]^+^ 351.1856; found 351.1847.

***N*-[(3-Methoxyphenyl)(phenyl)methyl]-3-phenylpyridin-2-amine (3v):** The reaction was carried out according to general procedure III with **1b** (130 mg, 0.5 mmol, 1 equiv.), 1-bromo-3-methoxybenzene (140 mg, 0.75 mmol, 1.5 equiv.), [RuCl_2_(*p*-cymene)]_2_ (7.6 mg, 0.0125 mmol, 2.5 mol-%), KOPiv (21 mg, 0.15 mmol, 30 mol-%), and K_2_CO_3_ (207 mg, 1.5 mmol, 3 equiv.) in dry toluene (2 mL). Colorless oil (117 mg, 64 % yield). ^1^H NMR (CDCl_3_, 200 MHz): *δ* = 3.70 (s, 3 H), 5.18 (d, *J* = 7.5 Hz, 1 H), 6.48 (d, *J* = 7.5 Hz, 1 H), 6.63 (dd, *J* = 7.3, 5.0 Hz, 1 H), 6.70–6.85 (m, 3 H), 7.13–7.43 (m, 12 H), 8.08 (dd, *J* = 5.0, 1.8 Hz, 1 H) ppm. ^13^C NMR (CDCl_3_, 50 MHz): *δ* = 55.2, 58.6, 112.3, 113.3, 113.4, 119.9, 122.4, 127.1, 127.5, 127.9, 128.6, 128.9, 129.3, 129.6, 137.3, 138.0, 143.3, 145.1, 147.4, 154.5, 159.8 ppm. HRMS: calcd. for C_25_H_22_N_2_O [M + H]^+^ 367.1805; found 367.1794.

**3-Phenyl-*N*-[phenyl(*p*-tolyl)methyl]pyridin-2-amine (3w):**^21^ The reaction was carried out according to general procedure III with **1b** (130 mg, 0.5 mmol, 1 equiv.), 1-bromo-4-methylbenzene (128 mg, 0.75 mmol, 1.5 equiv.), [RuCl_2_(*p*-cymene)]_2_ (7.6 mg, 0.0125 mmol, 2.5 mol-%), KOPiv (21 mg, 0.15 mmol, 30 mol-%), and K_2_CO_3_ (207 mg, 1.5 mmol, 3 equiv.) in dry toluene (2 mL). Colorless oil (117 mg, 67 % yield). ^1^H NMR (CDCl_3_, 200 MHz): *δ* = 2.29 (s, 3 H), 5.17 (d, *J* = 7.4 Hz, 1 H), 6.47 (d, *J* = 7.5 Hz, 1 H), 6.63 (dd, *J* = 7.2, 5.0 Hz, 1 H), 7.04–7.44 (m, 15 H), 8.08 (dd, *J* = 5.0, 1.8 Hz, 1 H) ppm. ^13^C NMR (CDCl_3_, 50 MHz): *δ* = 21.2, 58.4, 113.3, 122.3, 126.9, 127.4, 127.5, 127.9, 128.5, 129.0, 129.3, 129.4, 136.6, 137.3, 138.1, 140.5, 143.6, 147.4, 154.6 ppm. HRMS: calcd. for C_25_H_22_N_2_ [M + H]^+^ 351.1856; found 351.1873.

***N*-{[4-(*tert*-Butyl)phenyl](phenyl)methyl}-3-phenylpyridin-2-amine(3x):**^21^ The reaction was carried out according to general procedure III with **1b** (130 mg, 0.5 mmol, 1 equiv.), 1-bromo-4-(*tert*-butyl)benzene (160 mg, 0.75 mmol, 1.5 equiv.), [RuCl_2_(*p*-cymene)]_2_ (7.6 mg, 0.0125 mmol, 2.5 mol-%), KOPiv (21 mg, 0.15 mmol, 30 mol-%), and K_2_CO_3_ (207 mg, 1.5 mmol, 3 equiv.) in dry toluene (2 mL). Colorless solid (141 mg, 72 % yield); m.p. 74–76 °C. ^1^H NMR (CDCl_3_, 200 MHz): *δ* = 1.27 (s, 9 H), 5.20 (d, *J* = 7.5 Hz, 1 H), 6.50 (d, *J* = 7.6 Hz, 1 H), 6.62 (dd, *J* = 7.2, 5.0 Hz, 1 H), 7.12–7.44 (m, 15 H), 8.08 (dd, *J* = 5.0, 1.8 Hz, 1 H) ppm. ^13^C NMR (CDCl_3_, 50 MHz): *δ* = 31.5, 34.5, 58.2, 113.2, 122.3, 125.5, 126.9, 127.2, 127.5, 127.9, 128.5, 129.0, 129.4, 137.3, 138.1, 140.4, 143.7, 147.4, 149.8, 154.6 ppm (one phenyl-carbon resonance is overlapping). HRMS: calcd. for C_28_H_28_N_2_ [M + H]^+^ 393.2325; found 393.2349.

***N*-[(4-Butylphenyl)(phenyl)methyl]-3-phenylpyridin-2-amine (3y):** The reaction was carried out according to general procedure III with **1b** (130 mg, 0.5 mmol, 1 equiv.), 1-bromo-4-butylbenzene (160 mg, 0.75 mmol, 1.5 equiv.), [RuCl_2_(*p*-cymene)]_2_ (7.6 mg, 0.0125 mmol, 2.5 mol-%), KOPiv (21 mg, 0.15 mmol, 30 mol-%), and K_2_CO_3_ (207 mg, 1.5 mmol, 3 equiv.) in dry toluene (2 mL). Colorless oil (135 mg, 69 % yield). ^1^H NMR (CDCl_3_, 200 MHz): *δ* = 0.89 (t, *J* = 7.2 Hz, 3 H), 1.23–1.40 (m, 2 H), 1.47–1.62 (m, 2 H), 2.54 (t, *J* = 7.7 Hz, 2 H), 5.18 (d, *J* = 7.5 Hz, 1 H), 6.50 (d, *J* = 7.5 Hz, 1 H), 6.61 (dd, *J* = 7.2, 5.0 Hz, 1 H), 7.04–7.42 (m, 15 H), 8.07 (dd, *J* = 5.0, 1.8 Hz, 1 H) ppm. ^13^C NMR (CDCl_3_, 50 MHz): *δ* = 14.1, 22.5, 33.6, 35.4, 58.4, 113.2, 122.3, 126.9, 127.4, 127.5, 127.9, 128.5, 128.6, 128.9, 129.3, 137.3, 138.1, 140.7, 141.6, 143.7, 147.4, 154.6 ppm. HRMS: calcd. for C_28_H_28_N_2_ [M + H]^+^ 393.2325; found393.2323.

***N*-[(4-Chlorophenyl)(phenyl)methyl]-3-phenylpyridin-2-amine (3z):**^21^ The reaction was carried out according to general procedure III with **1b** (130 mg, 0.5 mmol, 1 equiv.), 1-bromo-4-chlorobenzene (143 mg, 0.75 mmol, 1.5 equiv.), [RuCl_2_(*p*-cymene)]_2_ (7.6 mg, 0.0125 mmol, 2.5 mol-%), KOPiv (21 mg, 0.15 mmol, 30 mol-%), and K_2_CO_3_ (207 mg, 1.5 mmol, 3 equiv.) in dry toluene (2 mL). Colorless oil (109 mg, 59 % yield). ^1^H NMR (CDCl_3_, 200 MHz): *δ* = 5.12 (d, *J* = 7.2 Hz, 1 H), 6.47 (d, *J* = 7.3 Hz, 1 H), 6.64 (dd, *J* = 7.2, 5.0 Hz, 1 H), 7.15–7.42 (m, 15 H), 8.07 (dd, *J* = 5.0, 1.8 Hz, 1 H) ppm. ^13^C NMR (CDCl_3_, 50 MHz): *δ* = 58.2, 113.6, 122.4, 127.3, 127.5, 128.0, 128.6, 128.7, 128.9, 129.4, 132.7, 137.4, 137.9, 142.0, 142.9, 147.3, 154.3 (one phenyl-carbon resonance is overlapping) ppm. HRMS: calcd. for C_24_H_19_N_2_Cl [M + H]^+^ 371.1310; found 371.1294.

**Ethyl 4-{Phenyl[(3-phenylpyridin-2-yl)amino]methyl}benzoate (3aa):** The reaction was carried out according to general procedure III with **1b** (130 mg, 0.5 mmol, 1 equiv.), ethyl 4-bromobenzoate (172 mg, 0.75 mmol, 1.5 equiv.), [RuCl_2_(*p*-cymene)]_2_ (7.6 mg, 0.0125 mmol, 2.5 mol-%), KOPiv (21 mg, 0.15 mmol, 30 mol-%), and K_2_CO_3_ (207 mg, 1.5 mmol, 3 equiv.) in dry toluene (2 mL). Colorless oil (86 mg, 42 % yield). ^1^H NMR (CDCl_3_, 200 MHz): *δ* = 1.35 (t, *J* = 7.1 Hz, 3 H), 4.33 (q, *J* = 7.1 Hz, 2 H), 5.17 (d, *J* = 7.2 Hz, 1 H), 6.53 (d, *J* = 7.2 Hz, 1 H), 6.66 (dd, *J* = 7.2, 5.0 Hz, 1 H), 7.16–7.45 (m, 13 H), 7.96 (d, *J* = 8.3 Hz, 2 H), 8.06 (dd, *J* = 5.0, 1.8 Hz, 1 H) ppm. ^13^C NMR (CDCl_3_, 50 MHz): *δ* = 14.4, 58.7, 60.9, 113.7, 122.5, 127.3, 127.4, 127.6, 128.0, 128.8, 128.9, 129.2, 129.4, 129.9, 137.4, 137.9, 142.7, 147.3, 148.6, 154.3, 166.6 ppm. HRMS: calcd. for C_27_H_24_N_2_O_2_ [M + H]^+^ 409.1911; found 409.1907.

**1-(4-{Phenyl[(3-phenylpyridin-2-yl)amino]methyl}phenyl)ethanone (3ab):**^21^ The reaction was carried out according to general procedure III with **1b** (130 mg, 0.5 mmol, 1 equiv.), 1-(4-bromophenyl)ethanone (149 mg, 0.75 mmol, 1.5 equiv.), [RuCl_2_(*p*-cymene)]_2_ (7.6 mg, 0.0125 mmol, 2.5 mol-%), KOPiv (21 mg, 0.15 mmol, 30 mol-%), and K_2_CO_3_ (207 mg, 1.5 mmol, 3 equiv.) in dry toluene (2 mL). Colorless oil (77 mg, 41 % yield). ^1^H NMR (CDCl_3_, 200 MHz): *δ* = 2.53 (s, 3 H), 5.18 (d, *J* = 7.1 Hz, 1 H), 6.52 (d, *J* = 7.1 Hz, 1 H), 6.66 (dd, *J* = 7.2, 5.0 Hz, 1 H), 7.19–7.44 (m, 13 H), 7.87 (d, *J* = 8.2 Hz, 2 H), 8.06 (dd, *J* = 5.0, 1.7 Hz, 1 H) ppm. ^13^C NMR (CDCl_3_, 50 MHz): *δ* = 26.7, 58.7, 113.7, 122.4, 127.4, 127.5, 127.6, 128.0, 128.7, 128.8, 128.9, 129.4, 135.9, 137.4, 137.8, 142.6, 147.3, 149.0, 154.3, 197.8 ppm. HRMS: calcd. for C_26_H_22_N_2_O [M + H]^+^ 379.1805; found 379.1799.

***N*-[(4-Isopropoxyphenyl)(phenyl)methyl]-3-methylpyridin-2-amine (3ae):** The reaction was carried out according to general procedure III with **1d** (128 mg, 0.5 mmol, 1 equiv.), bromobenzene (118 mg, 0.75 mmol, 1.5 equiv.), [RuCl_2_(*p*-cymene)]_2_ (7.6 mg, 0.0125 mmol, 2.5 mol-%), KOPiv (21 mg, 0.15 mmol, 30 mol-%), and K_2_CO_3_ (207 mg, 1.5 mmol, 3 equiv.) in dry toluene (2 mL). Colorless oil (71 mg, 43 % yield). ^1^H NMR (CDCl_3_, 200 MHz): *δ* = 1.30 (d, *J* = 6.0 Hz, 6 H), 2.11 (s, 3 H), 4.49 (sep, *J* = 6.0 Hz, 1 H), 4.61 (d, *J* = 7.0 Hz, 1 H), 6.44–6.52 (m, 2 H), 6.77–6.85 (m, 2 H), 7.17–7.34 (m, 8 H), 7.95 (dd, *J* = 5.0, 1.3 Hz, 1 H) ppm. ^13^C NMR (CDCl_3_, 50 MHz): *δ* = 17.2, 22.2, 58.0, 69.9, 113.1, 115.8, 116.4, 126.9, 127.5, 128.5, 128.9, 135.6, 137.0, 143.8, 145.8, 155.8, 157.0 ppm. HRMS calcd. for C_22_H_24_N_2_O [M + H]^+^ 333.1961; found 333.1963.

**3-Methyl-*N*-{phenyl[4-(trifluoromethyl)phenyl]methyl}pyridin-2-amine (3af):**^21^ The reaction was carried out according to general procedure III with **1g** (133 mg, 0.5 mmol, 1 equiv.), bromobenzene (118 mg, 0.75 mmol, 1.5 equiv.), [RuCl_2_(*p*-cymene)]_2_ (7.6 mg, 0.0125 mmol, 2.5 mol-%), KOPiv (21 mg, 0.15 mmol, 30 mol-%), and K_2_CO_3_ (207 mg, 1.5 mmol, 3 equiv.) in dry toluene (2 mL). Colorless solid (97 mg, 57 % yield); m.p. 56–58 °C. ^1^H NMR (CDCl_3_, 200 MHz): *δ* = 2.17 (s, 3 H), 4.65 (d, *J* = 6.3 Hz, 1 H), 6.54–6.60 (m, 2 H), 7.26–7.60 (m, 10 H), 7.97 (dd, *J* = 5.0, 1.3 Hz, 1 H) ppm. ^13^C NMR (CDCl_3_, 50 MHz): *δ* = 17.1, 58.6, 113.7, 116.6, 124.4 (q, *J* = 272.7 Hz), 125.5 (q, *J* = 3.8 Hz), 127.7, 127.8, 127.9, 128.9, 129.2 (q, *J* = 32.3 Hz), 137.2, 142.9, 145.7, 147.6, 155.5 ppm. HRMS: calcd. for C_20_H_17_F_3_N_2_ [M + H]^+^ 343.1417; found 343.1433.

**Methyl 4-{[(3-Methylpyridin-2-yl)amino] (phenyl)methyl}benzoate (3ag):**^21^ The reaction was carried out according to general procedure III with **1h** (128 mg, 0.5 mmol, 1 equiv.), bromobenzene (118 mg, 0.75 mmol, 1.5 equiv.), [RuCl_2_(*p*-cymene)]_2_ (7.6 mg, 0.0125 mmol, 2.5 mol-%), and KOPiv (21 mg, 0.15 mmol, 30 mol-%) in dry toluene (2 mL). Colorless oil (95 mg, 57 % yield). ^1^H NMR (CDCl_3_, 200 MHz): *δ* = 2.13 (s, 3 H), 3.86 (s, 3 H), 4.65 (d, *J* = 6.6 Hz, 1 H), 6.49–6.55 (m, 2 H), 7.21–7.33 (m, 6 H), 7.41 (d, *J* = 8.2 Hz, 2 H), 7.92–7.99 (m, 3 H) ppm. ^13^C NMR (CDCl_3_, 50 MHz): *δ* = 17.1, 52.1, 58.7, 113.5, 116.5, 127.4, 127.5, 127.9, 128.8, 129.9, 137.1, 142.9, 145.7, 148.8, 155.5, 167.1 ppm. HRMS: calcd. for C_21_H_20_N_2_O_2_ [M + H]^+^ 333.1598; found 333.1587.

***N*-(Diphenylmethylene)-3-methylpyridin-2-amine (4):**^7^ The reaction was carried out according to general procedure III with *N*-benzylidene-3-methylpyridin-2-amine (**12**, 98 mg, 0.5 mmol, 1 equiv.), 1-bromobenzene (118 mg, 0.75 mmol, 1.5 equiv.), [RuCl_2_(*p*-cymene)]_2_ (7.6 mg, 0.0125 mmol, 2.5 mol-%), and KOPiv (21 mg, 0.15 mmol, 30 mo l%) in dry toluene (2 mL). Yellow oil (91 mg, 67 % yield); The analytical data are in accordance with the literature values.^12^ ^1^H NMR (CDCl_3_, 200 MHz): *δ* = 2.08 (s, 3 H), 6.76 (dd, *J* = 7.4, 4.9 Hz, 1 H), 7.20–7.42 (m, 9 H), 7.82 (d, *J* = 6.5 Hz, 2 H), 8.09 (d, *J* = 4.8 Hz, 1 H) ppm. ^13^C NMR (CDCl_3_, 50 MHz): *δ* = 17.5, 118.8, 122.8, 127.8, 128.1, 128.9, 129.7, 138.1, 145.7, 162.3, 169.4 ppm.

**3-Methyl-2-(phenylethynyl)pyridine:**^22^ 2-Bromo-3-methylpyridine (172 mg, 1 mmol, 1 equiv.), phenylacetylene (122 mg, 1.2 mmol, 1.2 equiv.), pyrrolidine (142 mg, 2 mmol, 2 equiv.), PdCl_2_ (4 mg, 0.02 mmol, 2 mol-%), PPh_3_ (10 mg, 0.04 mmol, 4 mol-%), and degassed water (2 mL) were placed in an oven-dried 6 mL vial with a Teflon cap and a magnetic stirring bar. The reaction vial was then heated in a reaction block at 120 °C for 3 h. The reaction mixture was cooled to room temp. and extracted with diethyl ether (4 × 5 mL). The combined organic layers were dried with Na_2_SO_4_, filtered, and concentrated. The residue was purified by flash chromatography (PE/EtOAc = 9:1) to give the pure product as a red oil (139 mg, 72 % yield). ^1^H NMR (CDCl_3_, 200 MHz): *δ* = 2.52 (s, 3 H), 7.15 (dd, *J* = 7.7, 4.8 Hz, 1 H), 7.33–7.40 (m, 3 H), 7.51–7.63 (m, 3 H), 8.45 (dd, *J* = 4.7, 1.0 Hz, 1 H) ppm. ^13^C NMR (CDCl_3_, 50 MHz): *δ* = 19.6, 87.6, 93.2, 122.6, 122.8, 128.5, 129.0, 132.1, 136.0, 137.1, 143.2, 147.5 ppm.

**3-Methyl-2-phenethylpyridine (5):**^23^ 3-Methyl-2-(phenylethynyl)pyridine (193 mg, 1 mmol, 1 equiv.), triethylamine (253 mg, 2.5 mmol, 2.5 equiv.), 10 % palladium on carbon (30 mg), and EtOH (30 mL) were charged in a round-bottomed flask. The reaction mixture was stirred at room temperature under H_2_ at atmospheric pressure for 16 h. The solvent was removed under reduced pressure, and the residue dissolved in Et_2_O (25 mL). The solid material was removed by filtration. The organic layer was washed with saturated NaHCO_3_ and then brine, dried with Na_2_SO_4_, filtered, and concentrated. Pale yellow oil (196 mg, 99 % yield). ^1^H NMR (CDCl_3_, 200 MHz): *δ* = 2.23 (s, 3 H), 3.02–3.10 (m, 4 H) 7.05 (dd, *J* = 7.6, 4.8 Hz, 1 H), 7.19–7.42 (m, 6 H), 8.42 (d, *J* = 3.7 Hz, 1 H) ppm. ^13^C NMR (CDCl_3_, 50 MHz): *δ* = 18.8, 35.1, 37.5, 121.4, 126.0, 128.5, 128.6, 131.3, 137.7, 142.1, 146.8, 159.6 ppm.

**2-(Benzyloxy)-3-methylpyridine (6):**^24^ 2-Chloro-3-methylpyridine (128 mg, 1 mmol, 1 equiv.), phenylmethanol (140 mg, 1.3 mmol, 1.3 equiv.), KO*t*Bu (224 mg, 2 mmol, 2 equiv.), and dioxane (5 mL) were charged in a round-bottomed flask. The reaction mixture was heated to reflux for 24 h. The solution was cooled to room temp., and H_2_O (2 mL) was added. The aqueous phase was extracted with EtOAc (3 × 5 mL). The combined organic layer was washed with saturated NaHCO_3_ and then brine, dried with Na_2_SO_4_, filtered, and concentrated. The residue was purified by flash chromatography (PE/EtOAc = 19:1) to give the pure product. Colorless oil (140 mg, 70 % yield). ^1^H NMR (CDCl_3_, 200 MHz): *δ* = 2.26 (s, 3 H), 5.44 (s, 2 H), 6.82 (dd, *J* = 7.1, 5.1 Hz, 1 H), 7.31–7.52 (m, 6 H), 8.03 (dd, *J* = 5.0, 1.3 Hz, 1 H) ppm. ^13^C NMR (CDCl_3_, 50 MHz): *δ* = 16.0, 67.3, 116.9, 121.1, 127.6, 127.7, 128.5, 138.0, 138.7, 144.1, 162.0 ppm.

***N*-Benzyl-*N*,3-dimethylpyridin-2-amine (7a):**^21^ The reaction was carried out according to general procedure II with 2-bromo-3-methylpyridine (172 mg, 1 mmol, 1 equiv.), *N*-methyl-1-phenylmethanamine (169 mg, 1.4 mmol, 1.4 equiv.), NaO*t*Bu (192 mg, 2 mmol, 2 equiv.), Pd_2_(dba)_3_ (18 mg, 0.02 mmol, 2 mol-%), and DPPP (16 mg, 0.04 mmol, 4 mol-%) in dry toluene (4 mL). Colorless oil (186 mg, 88 % yield). ^1^H NMR (CDCl_3_, 200 MHz): *δ* = 2.32 (s, 3 H), 2.75 (s, 3 H), 4.33 (s, 2 H), 6.82 (dd, *J* = 7.3, 4.9 Hz, 1 H), 7.24–7.40 (m, 6 H), 8.16 (dd, *J* = 4.8, 1.5 Hz, 1 H) ppm. ^13^C NMR (CDCl_3_, 50 MHz): *δ* = 19.0, 39.1, 57.8, 117.4, 124.5, 126.9, 128.0, 128.4, 139.3, 139.5, 145.2, 162.6 ppm. HRMS: calcd. for C_14_H_16_N_2_ [M + H]^+^ 213.1386; found 213.1385.

**2-(Pyridin-2-yl)-1,2,3,4-tetrahydroisoquinoline (7b):**^25^ The reaction was carried out according to general procedure II with 2-bromopyridine (158 mg, 1 mmol, 1 equiv.), 1,2,3,4-tetrahydroisoquinoline (186 mg, 1.4 mmol, 1.4 equiv.), NaO*t*Bu (192 mg, 2 mmol, 2 equiv.), Pd_2_(dba)_3_ (18 mg, 0.02 mmol, 2 mol-%), and DPPP (16 mg, 0.04 mmol, 4 mol-%) in dry toluene (4 mL). Colorless solid (199 mg, 95 % yield); m.p. 47–49 °C. ^1^H NMR (CDCl_3_, 200 MHz): *δ* = 2.98 (t, *J* = 5.9 Hz, 2 H), 3.86 (t, *J* = 5.9 Hz, 2 H), 4.72 (s, 2 H), 6.59–6.71 (m, 2 H), 7.21 (q, *J* = 3.1 Hz, 4 H), 7.46–7.55 (m, 1 H), 8.25 (dd, *J* = 4.9, 1.2 Hz, 1 H) ppm. ^13^C NMR (CDCl_3_, 50 MHz): *δ* = 29.1, 42.6, 47.2, 106.7, 112.6, 126.3, 126.5, 126.7, 128.5, 134.5, 135.5, 137.5, 148.1, 158.8 ppm.

**2-(3-Methylpyridin-2-yl)-1,2,3,4-tetrahydroisoquinoline (7c):**^21^ The reaction was carried out according to general procedure II with 2-bromo-3-methylpyridine (172 mg, 1 mmol, 1 equiv.), 1,2,3,4-tetrahydroisoquinoline (186 mg, 1.4 mmol, 1.4 equiv.), NaO*t*Bu (192 mg, 2 mmol, 2 equiv.), Pd_2_(dba)_3_ (18 mg, 0.02 mmol, 2 mol-%), and DPPP (16 mg, 0.04 mmol, 4 mol-%) in dry toluene (4 mL). Yellow oil (203 mg, 91 % yield). ^1^H NMR (CDCl_3_, 200 MHz): *δ* = 2.35 (s, 3 H), 3.07 (t, *J* = 5.8 Hz, 2 H), 3.41 (t, *J* = 5.8 Hz, 2 H), 4.45 (s, 2 H), 6.88 (dd, *J* = 7.3, 4.9 Hz, 1 H), 7.19 (s, 4 H), 7.42–7.46 (m, 1 H), 8.22 (dd, *J* = 4.8, 1.8 Hz, 1 H) ppm. ^13^C NMR (CDCl_3_, 50 MHz): *δ* = 18.5, 29.9, 48.5, 51.6, 117.8, 124.9, 125.9, 126.2, 126.9, 128.9, 134.6, 135.4, 139.4, 145.3, 161.9 ppm. HRMS: calcd. for C_15_H_16_N_2_ [M + H]^+^ 225.1386; found 225.1378.

**Supporting Information** (see footnote on the first page of this article): Full experimental details and spectra.

## Supplementary material

As a service to our authors and readers, this journal provides supporting information supplied by the authors. Such materials are peer reviewed and may be re-organized for online delivery, but are not copy-edited or typeset. Technical support issues arising from supporting information (other than missing files) should be addressed to the authors.
