# Supplementary material for: Aryl Bromides and Aryl Chlorides for the Direct Arylation of Benzylic Amines Mediated by Ruthenium(II)
Source: European J Org Chem. 2013 Mar 22;2013(14):2878–90. doi: 10.1002/ejoc.201300004 (PMC3698694; doi:10.1002/ejoc.201300004)
Supplement: Supplementary file 1 [file ejoc2013-2878-SD1.pdf]

Eur. J. Org. Chem. **2013** · © WILEY-VCH Verlag GmbH & Co. KGaA, 69451 Weinheim, 2013 · ISSN 1099–0690

SUPPORTING INFORMATION

DOI: 10.1002/ejoc.201300004
Title: Aryl Bromides and Aryl Chlorides for the Direct Arylation of Benzylic Amines Mediated by Ruthenium(II)
Author(s): Navid Dastbaravardeh, Michael Schnürch,* Marko D. Mihovilovic

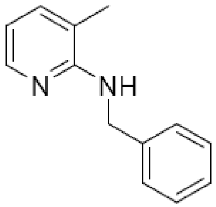

1a

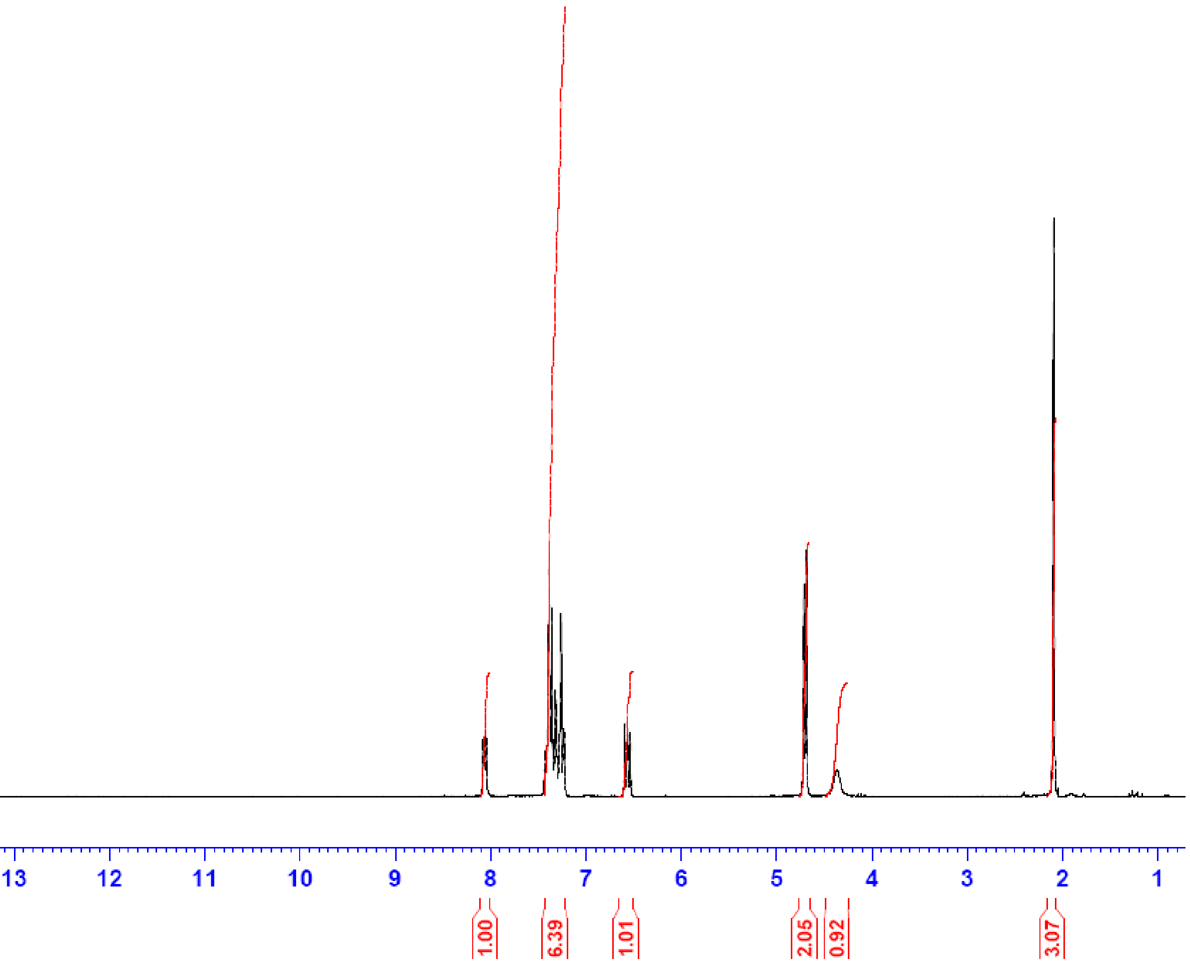

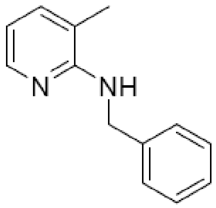
N
NH

1a

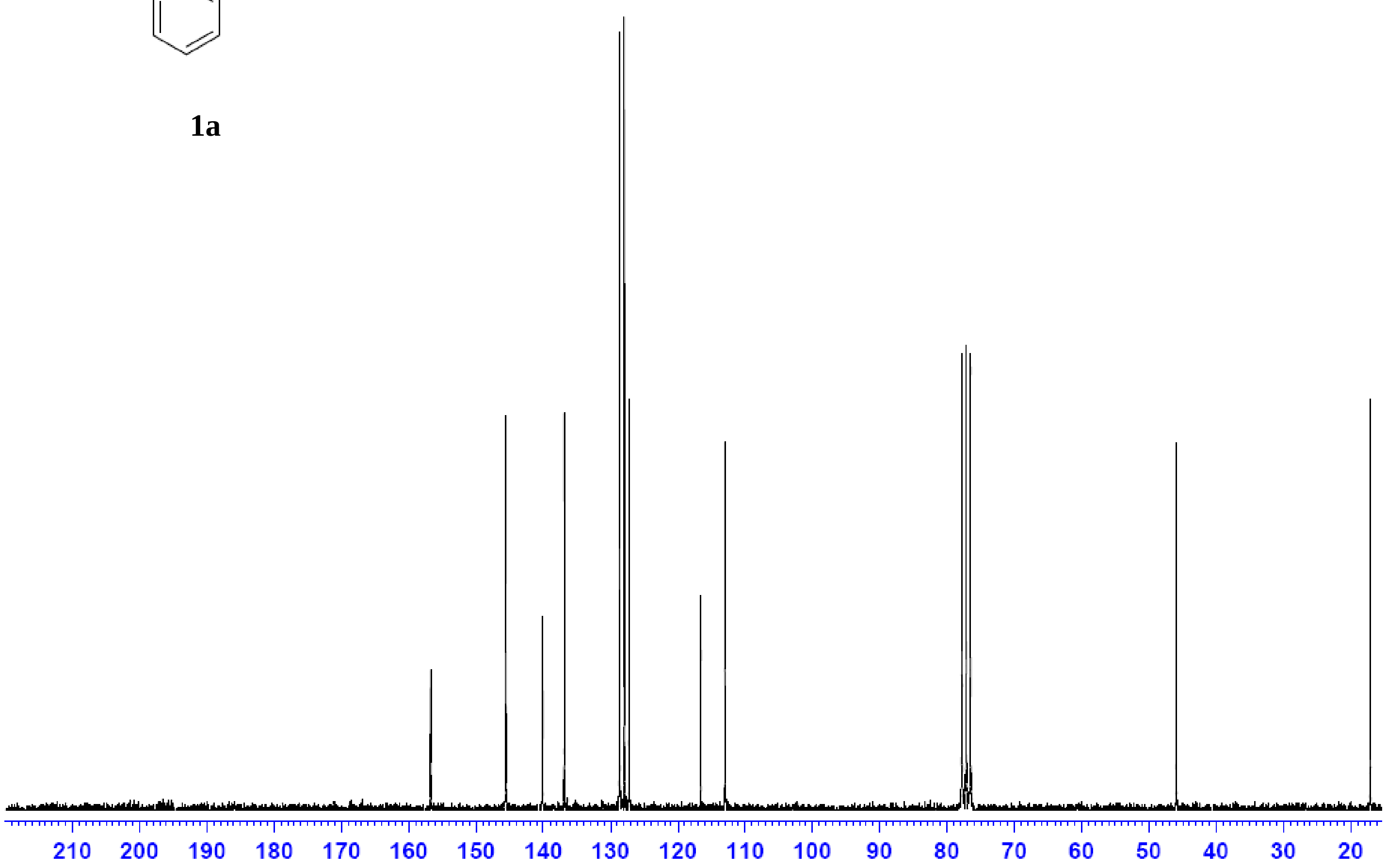
156.76
145.58
140.10
136.94
128.69
127.97
127.28
116.59
113.01
77.80
77.16
76.53
45.93
17.09
210 200 190 180 170 160 150 140 130 120 110 100 90 80 70 60 50 40 30 20

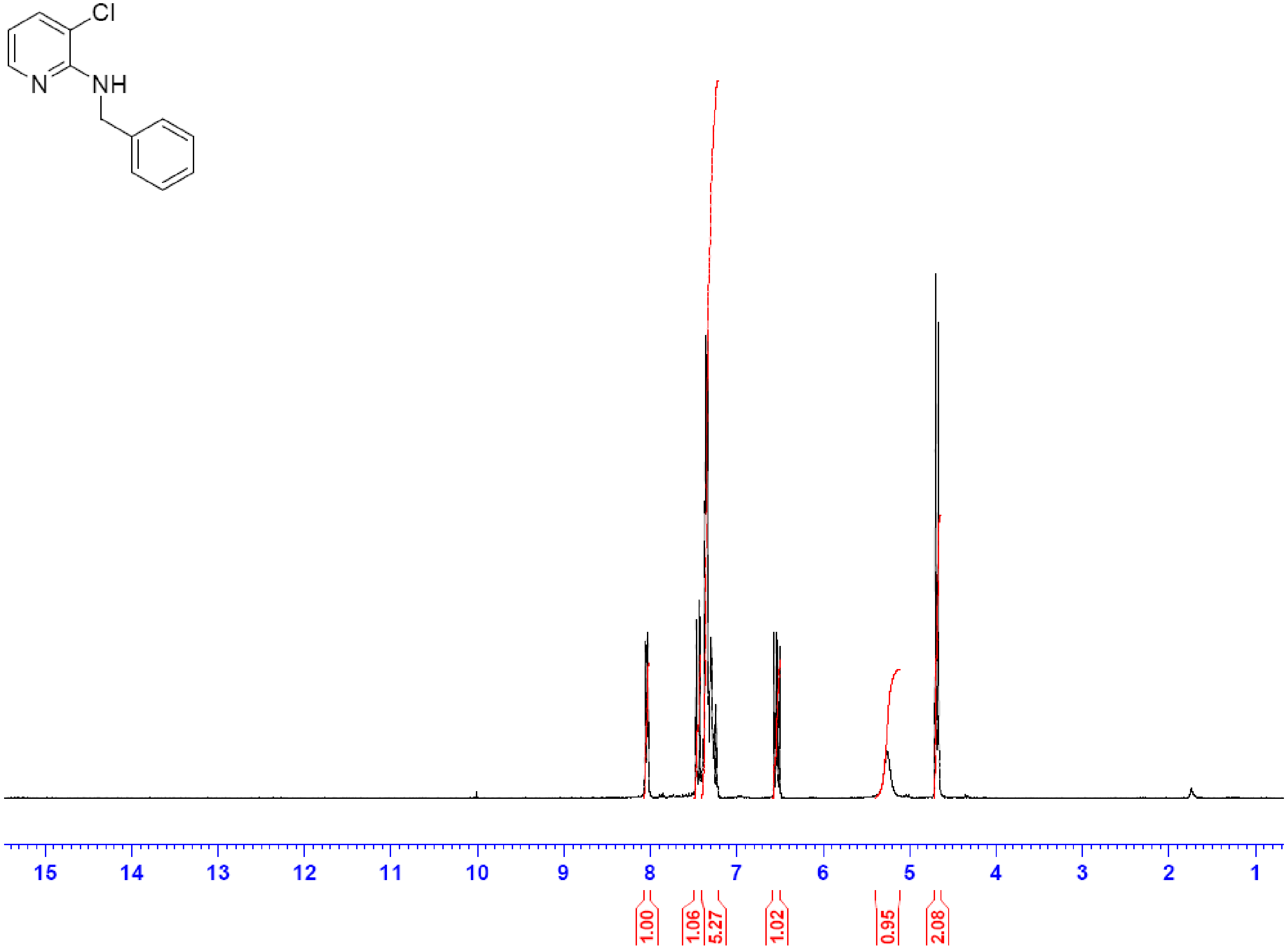
Cl
N
NH
15 14 13 12 11 10 9 8 7 6 5 4 3 2 1
1.00
1.06
5.27
1.02
0.95
2.08

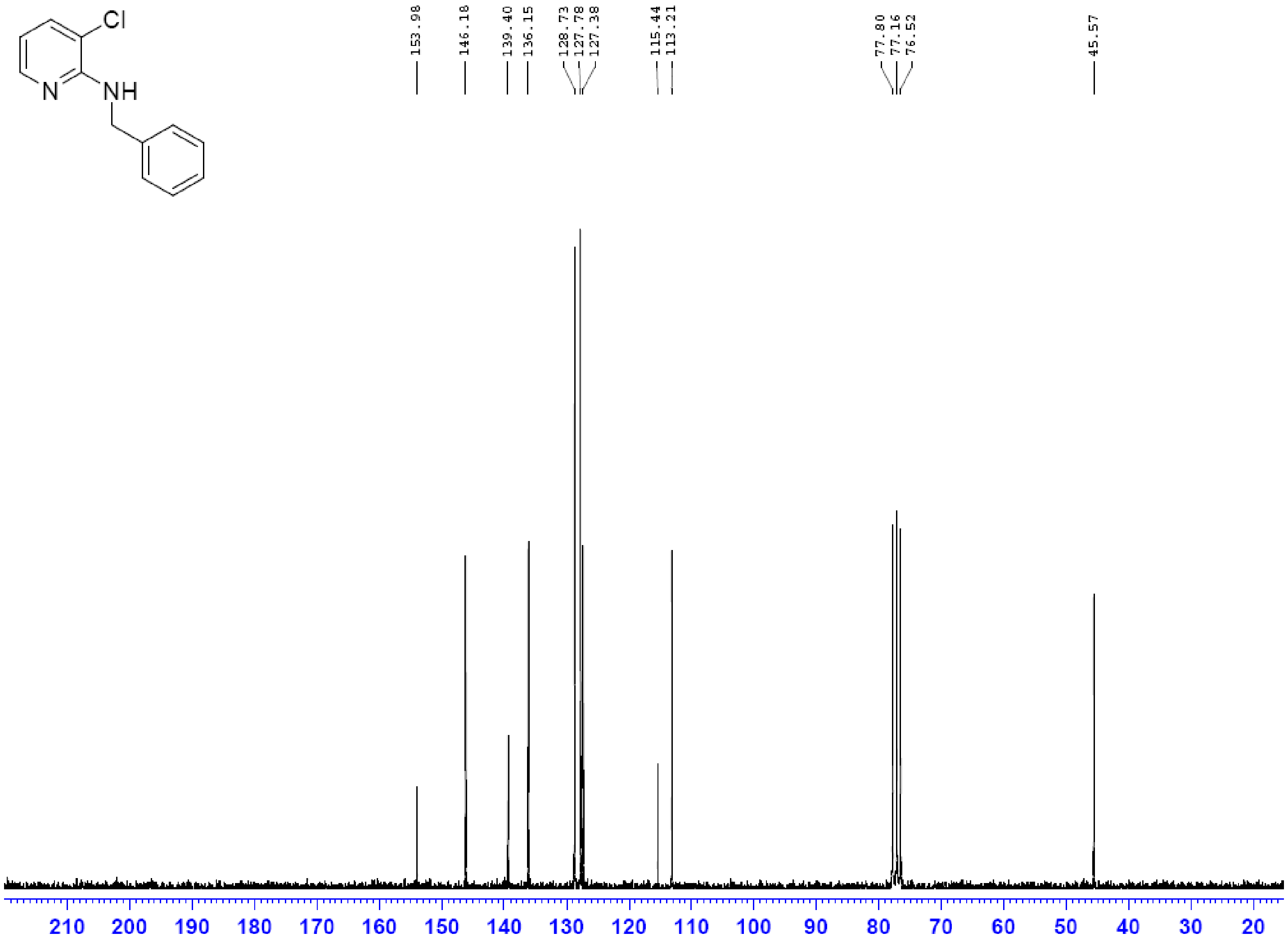

Cl
N
NH
153.98
146.18
139.40
136.15
128.73
127.78
127.38
115.44
113.21
77.80
77.16
76.52
45.57
210
200
190
180
170
160
150
140
130
120
110
100
90
80
70
60
50
40
30
20

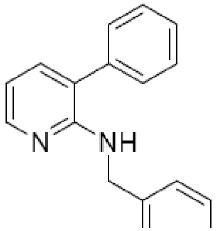

1b

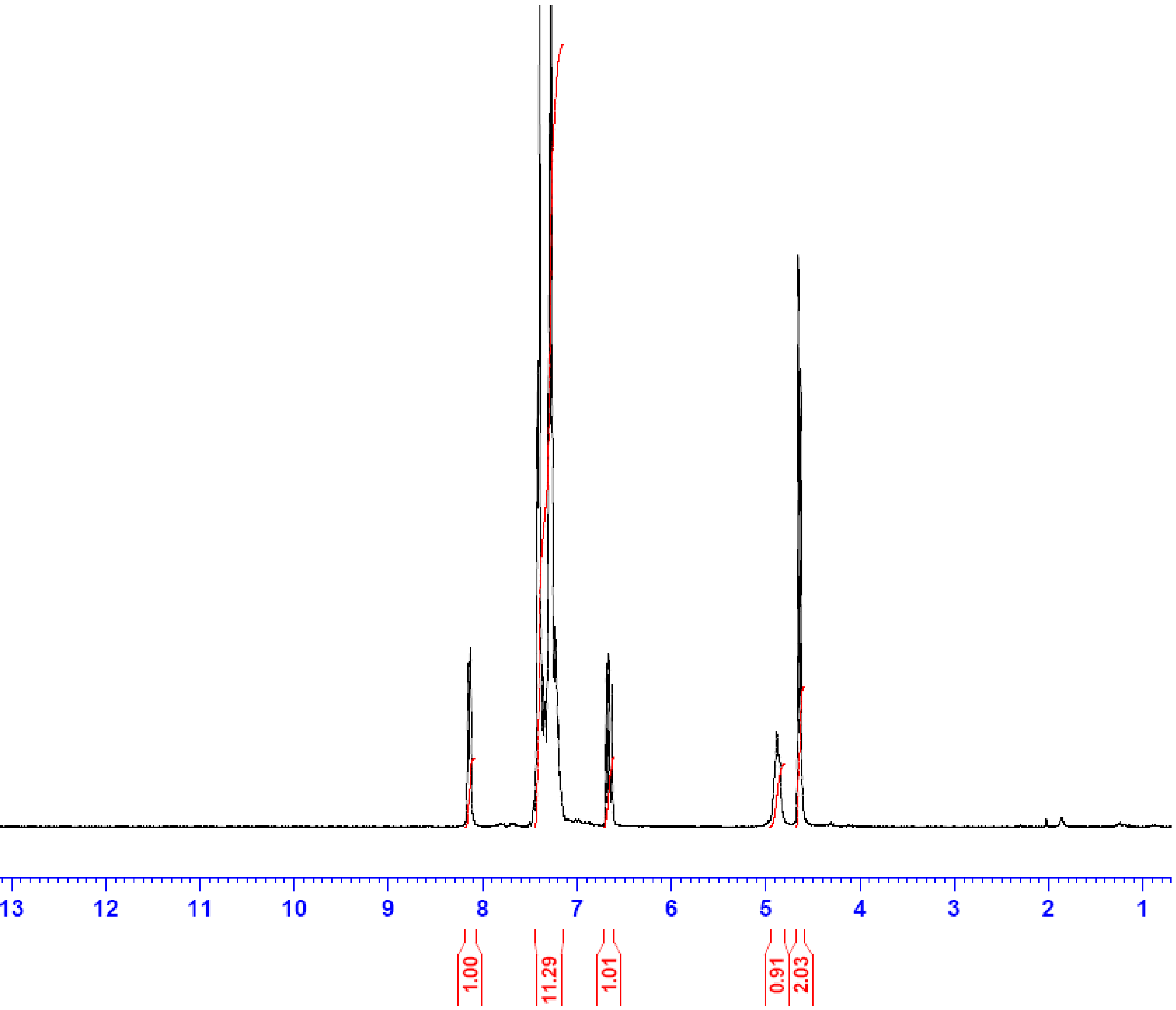

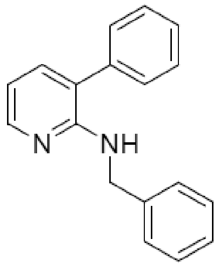
N
NH

1b

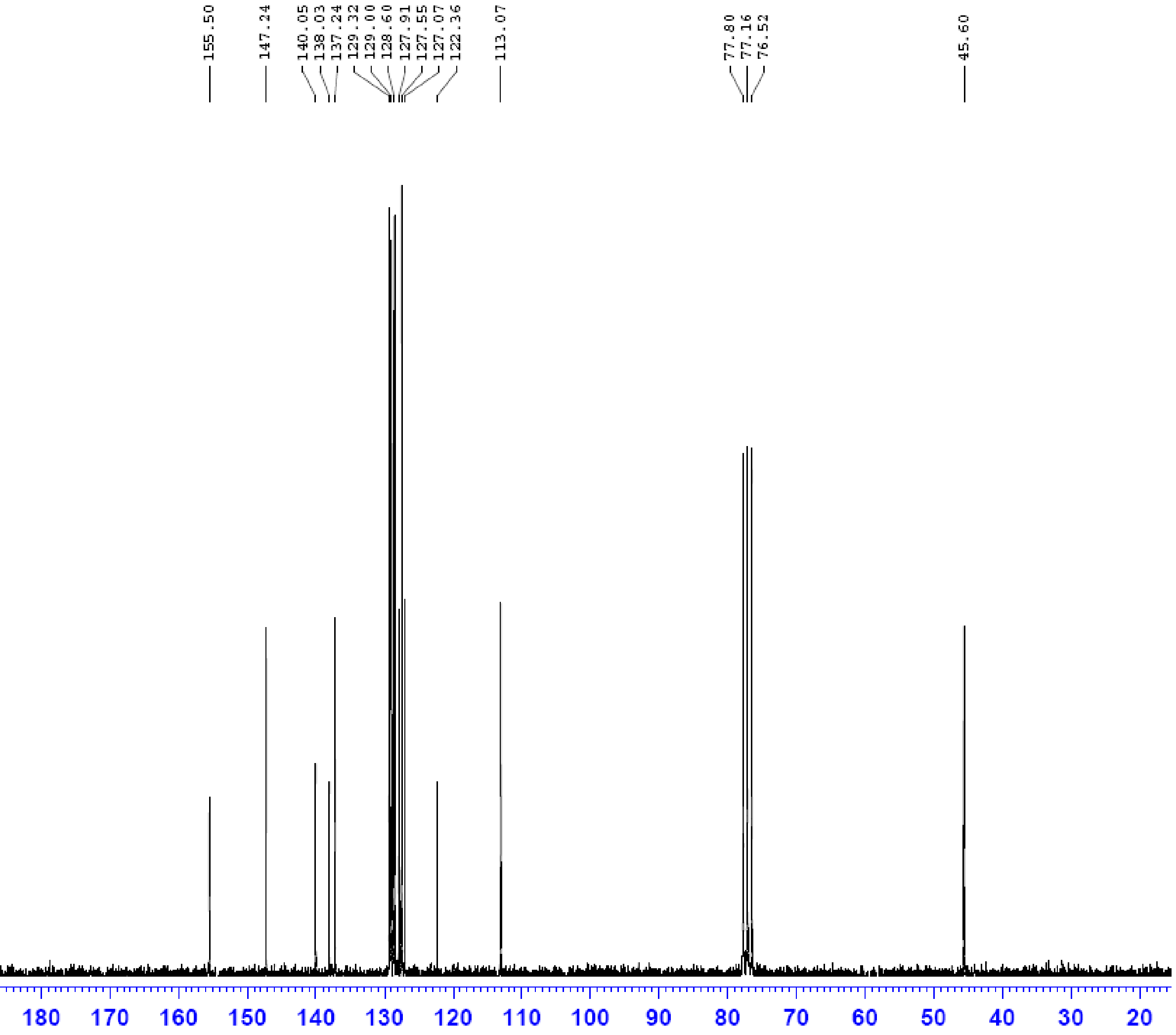
155.50
147.24
140.05
138.03
137.24
129.32
129.00
128.60
127.91
127.55
127.07
122.36
113.07
77.80
77.16
76.52
45.60
210 200 190 180 170 160 150 140 130 120 110 100 90 80 70 60 50 40 30 20

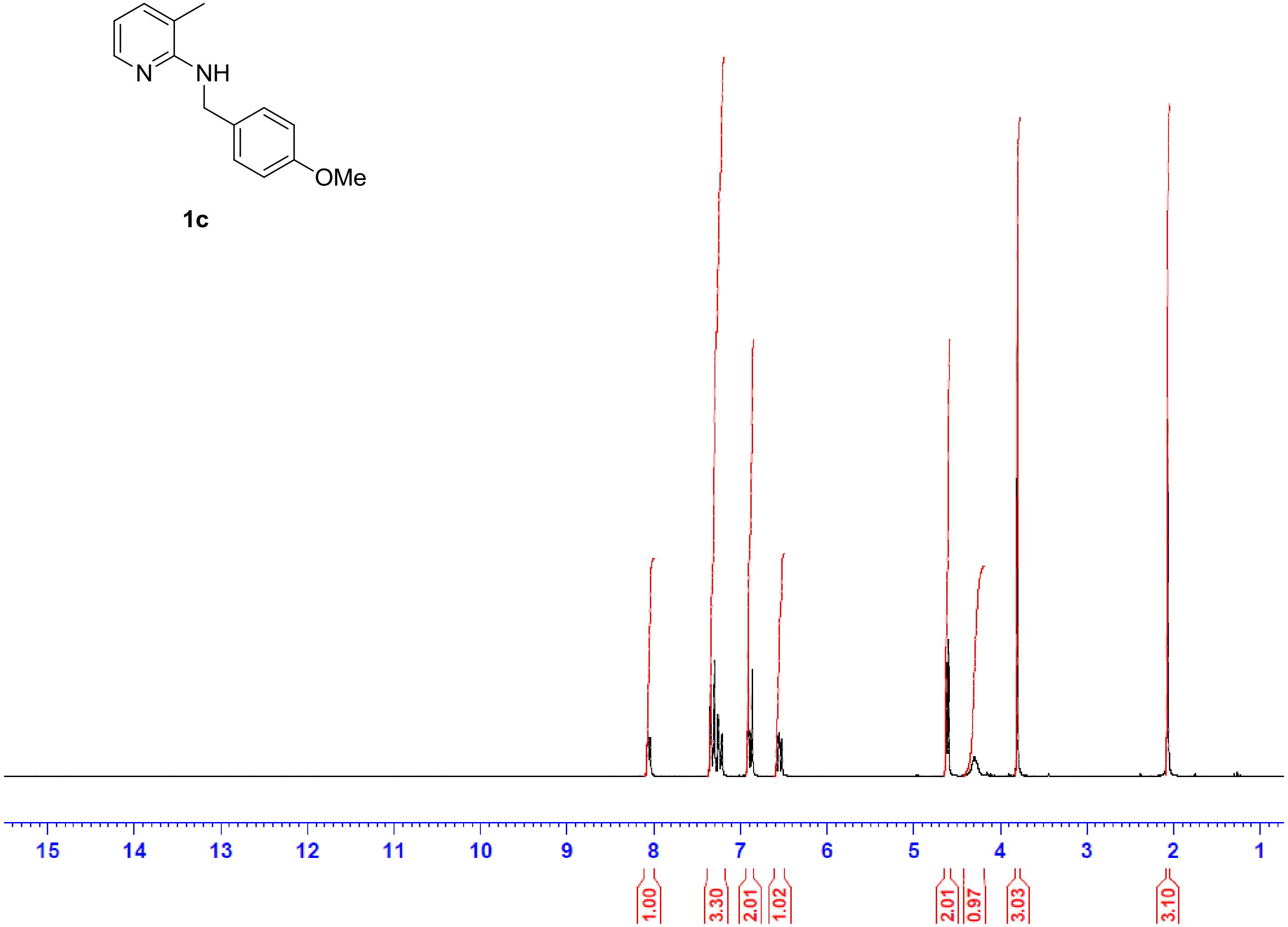

NH
N
OMe
15 14 13 12 11 10 9 8 7 6 5 4 3 2 1
1.00
3.30
2.01
1.02
2.01
0.97
3.03
3.10

1c

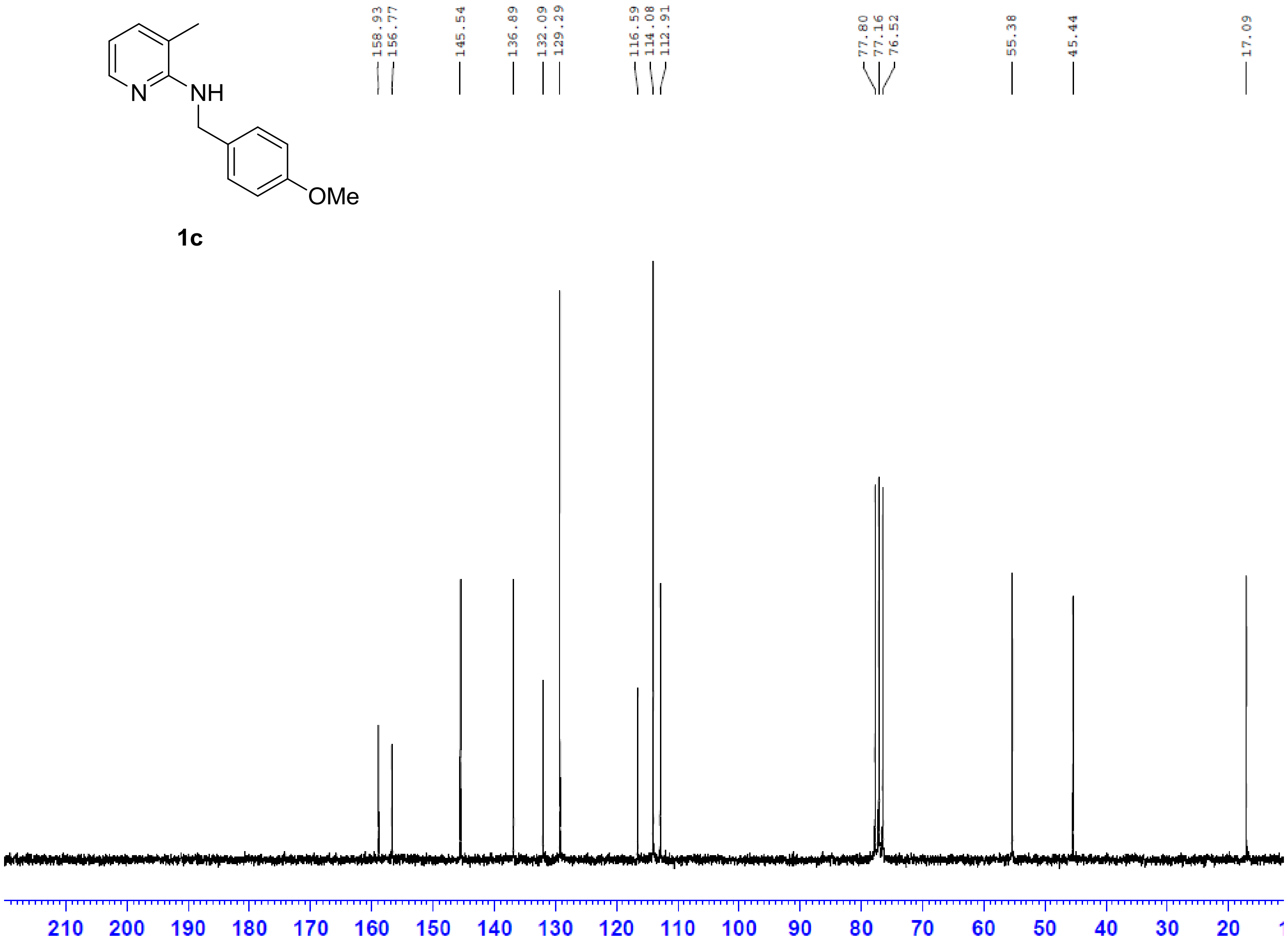

NH
N
OMe
158.93
156.77
145.54
136.89
132.09
129.29
116.59
114.08
112.91
77.80
77.16
76.52
55.38
45.44
17.09
210
200
190
180
170
160
150
140
130
120
110
100
90
80
70
60
50
40
30
20

1c

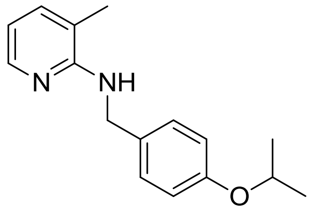
NH
N
O

1d

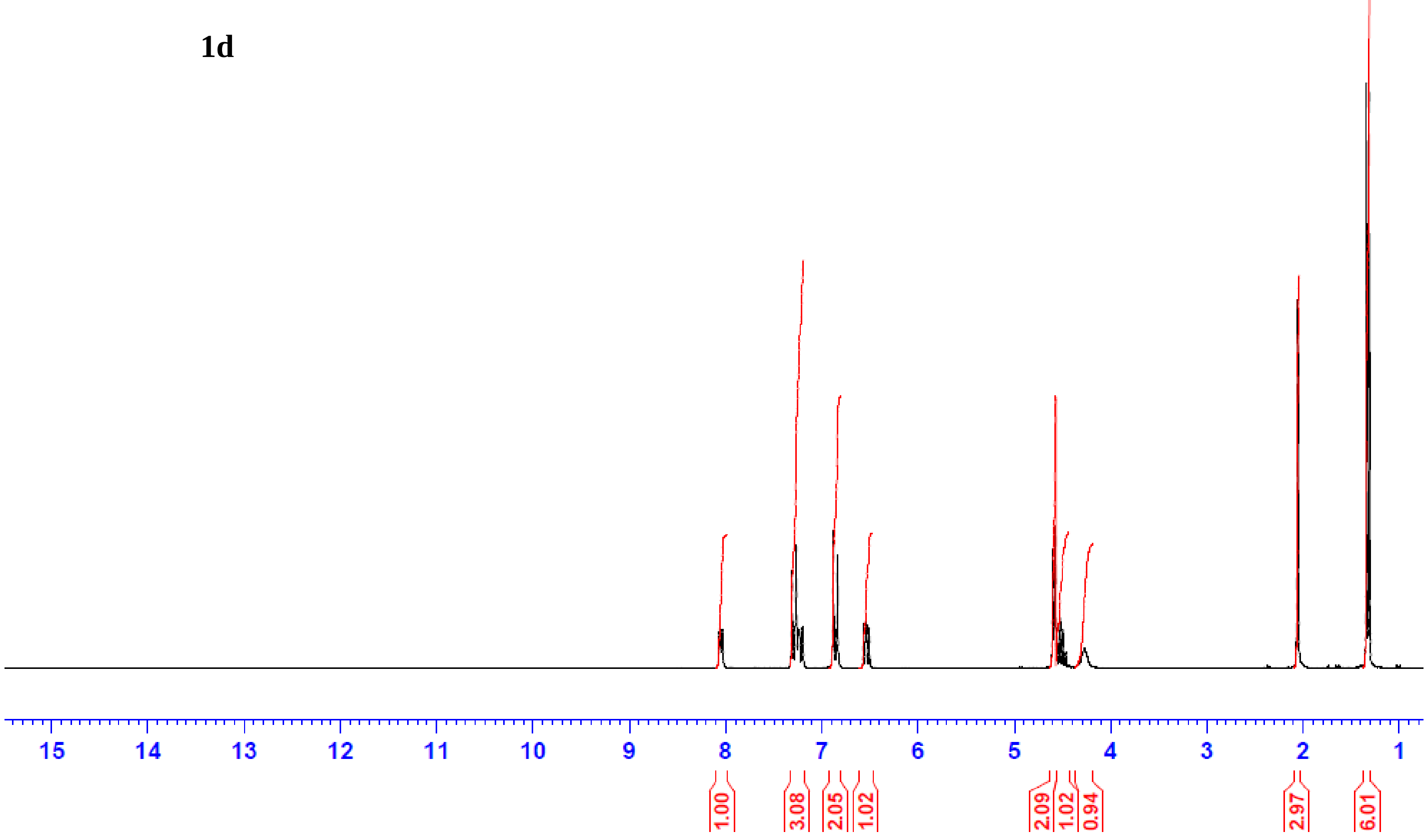
15 14 13 12 11 10 9 8 7 6 5 4 3 2 1
1.00
3.08
2.05
1.02
2.09
1.02
0.94
2.97
6.01

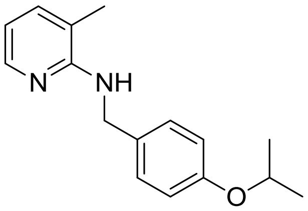
N
NH
O

1d

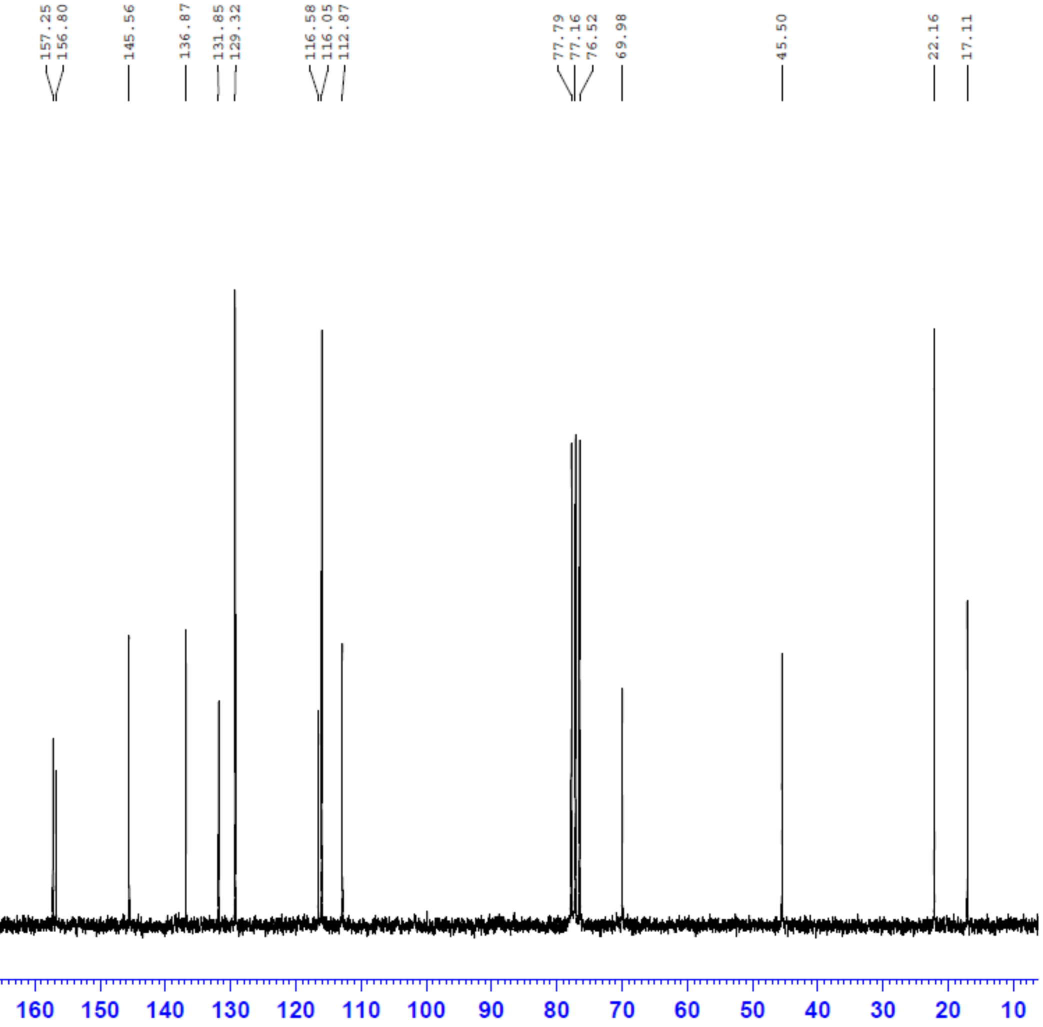
157.25
156.80
145.56
136.87
131.85
129.32
116.58
116.05
112.87
77.79
77.16
76.52
69.98
45.50
22.16
17.11
220 210 200 190 180 170 160 150 140 130 120 110 100 90 80 70 60 50 40 30 20 10

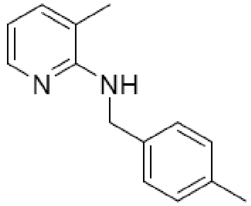

1e

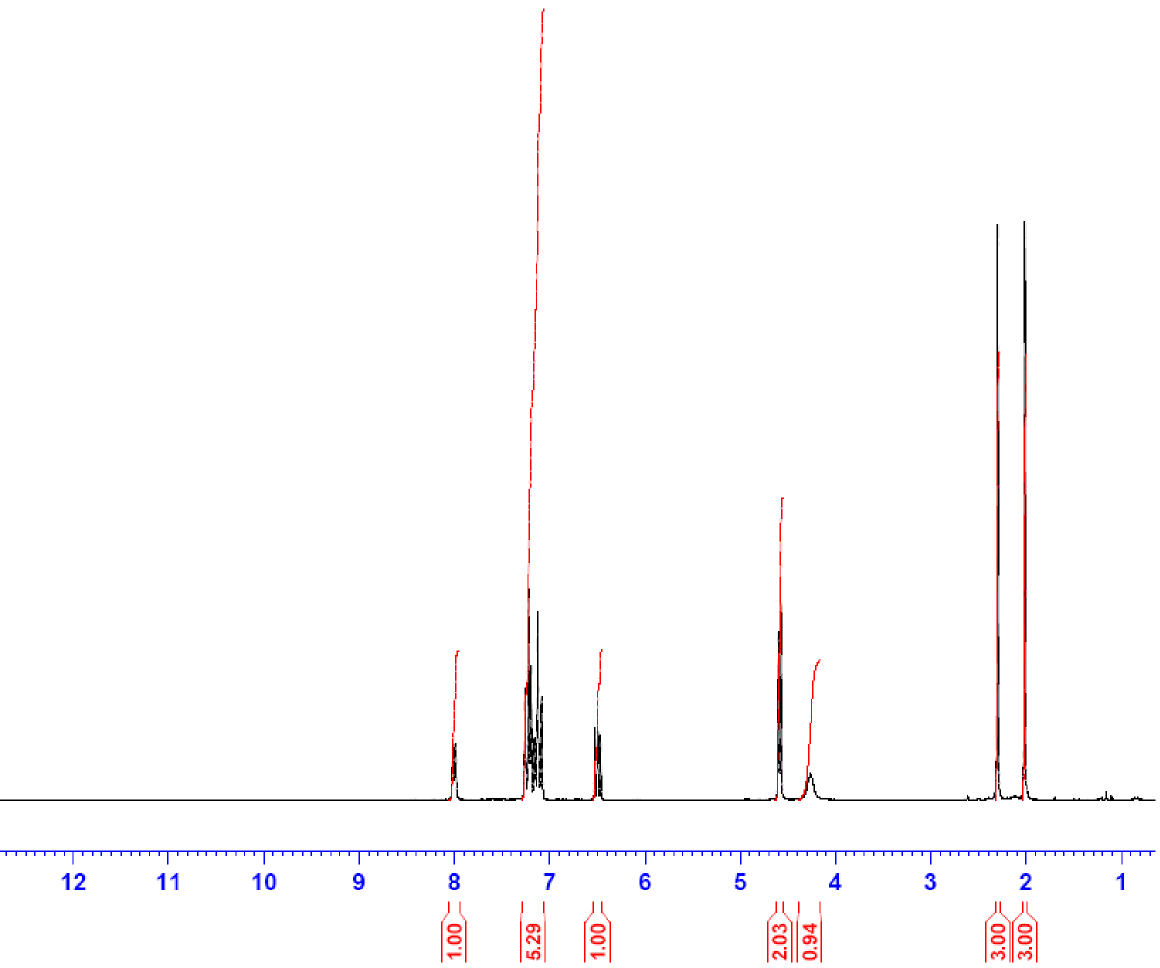

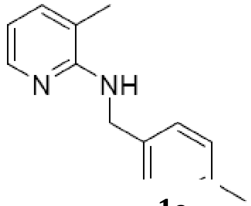

1e

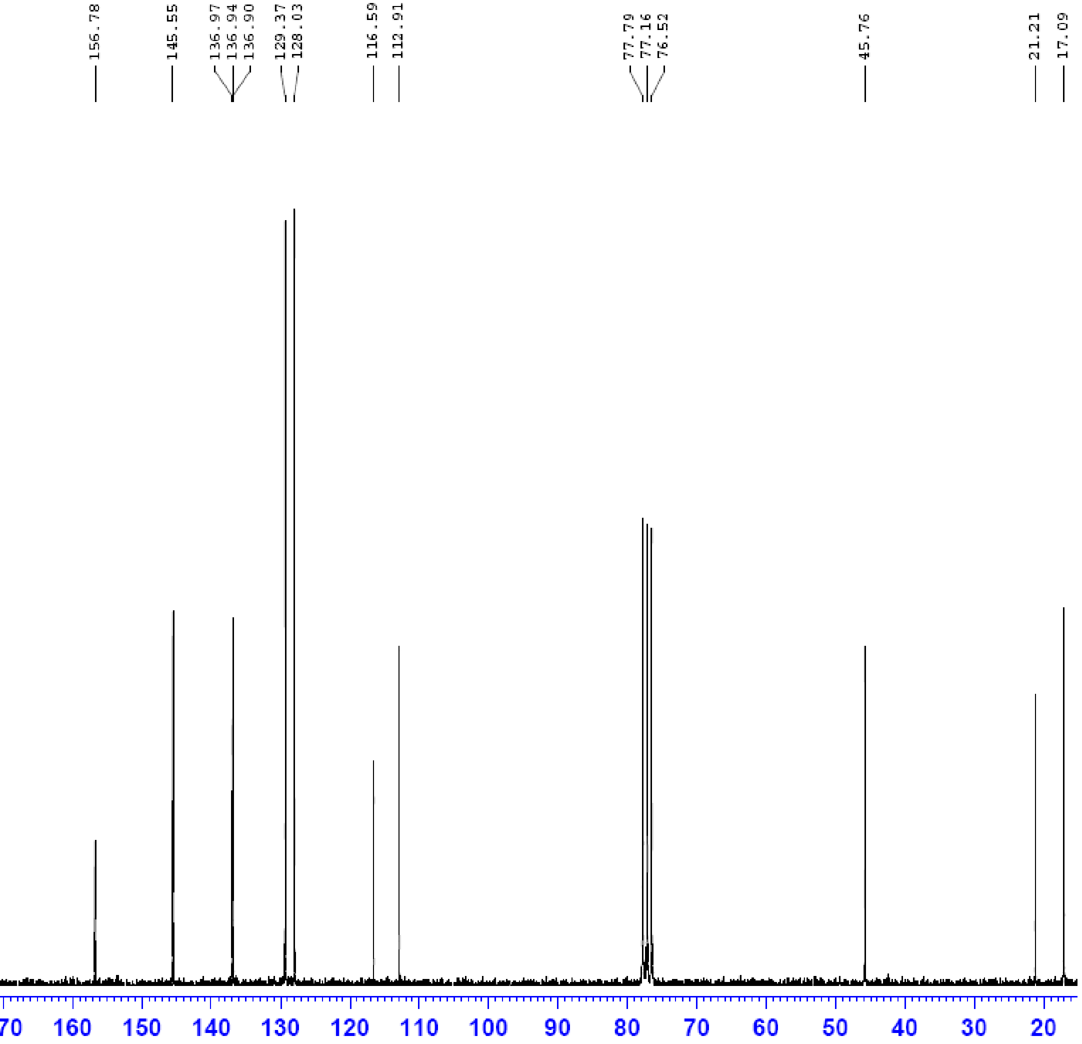
156.78
145.55
136.97
136.94
136.90
129.37
128.03
116.59
112.91
77.79
77.16
76.52
45.76
21.21
17.09
210 200 190 180 170 160 150 140 130 120 110 100 90 80 70 60 50 40 30 20

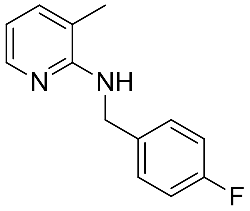

1f

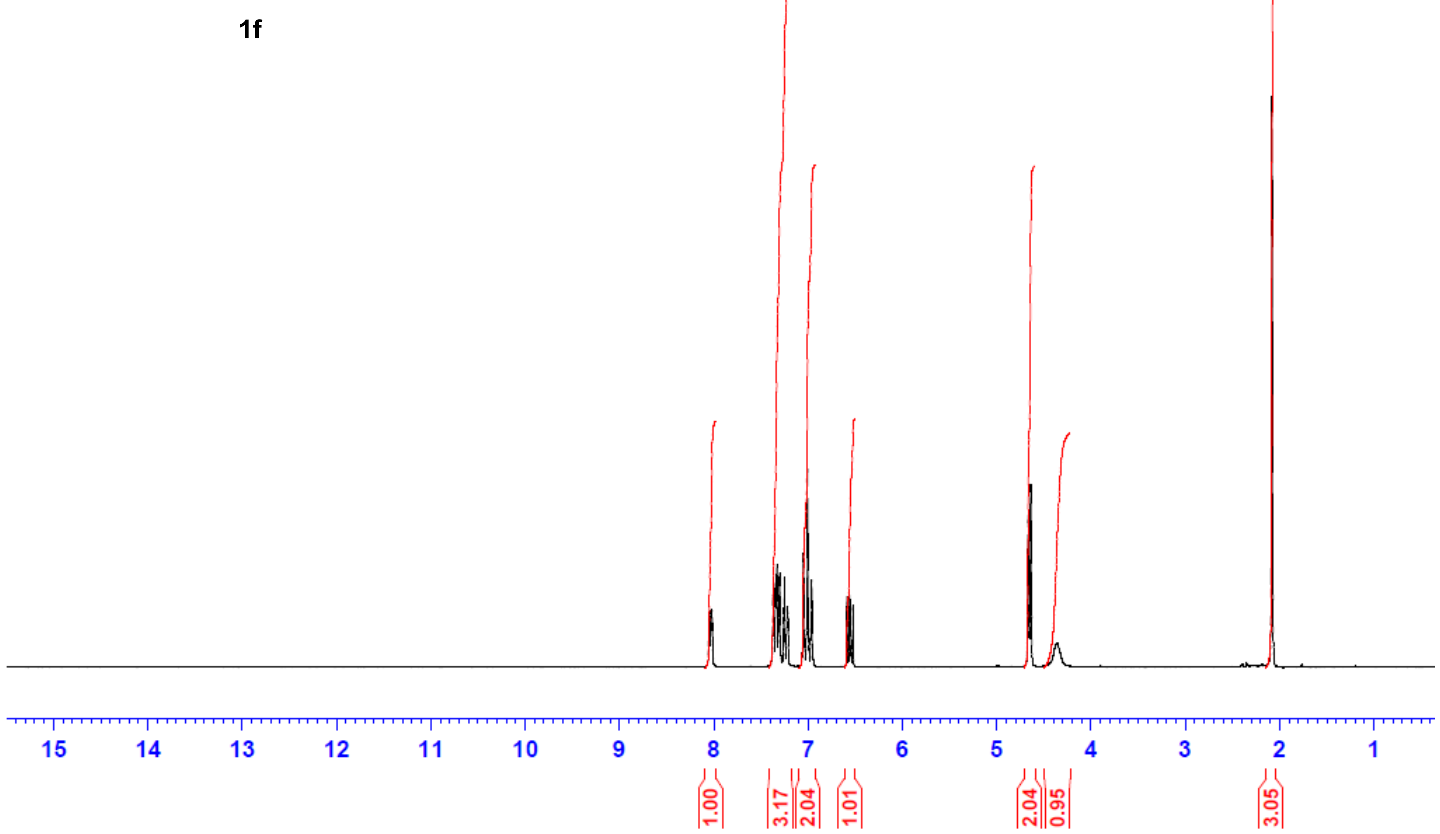

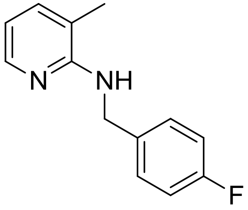

1f

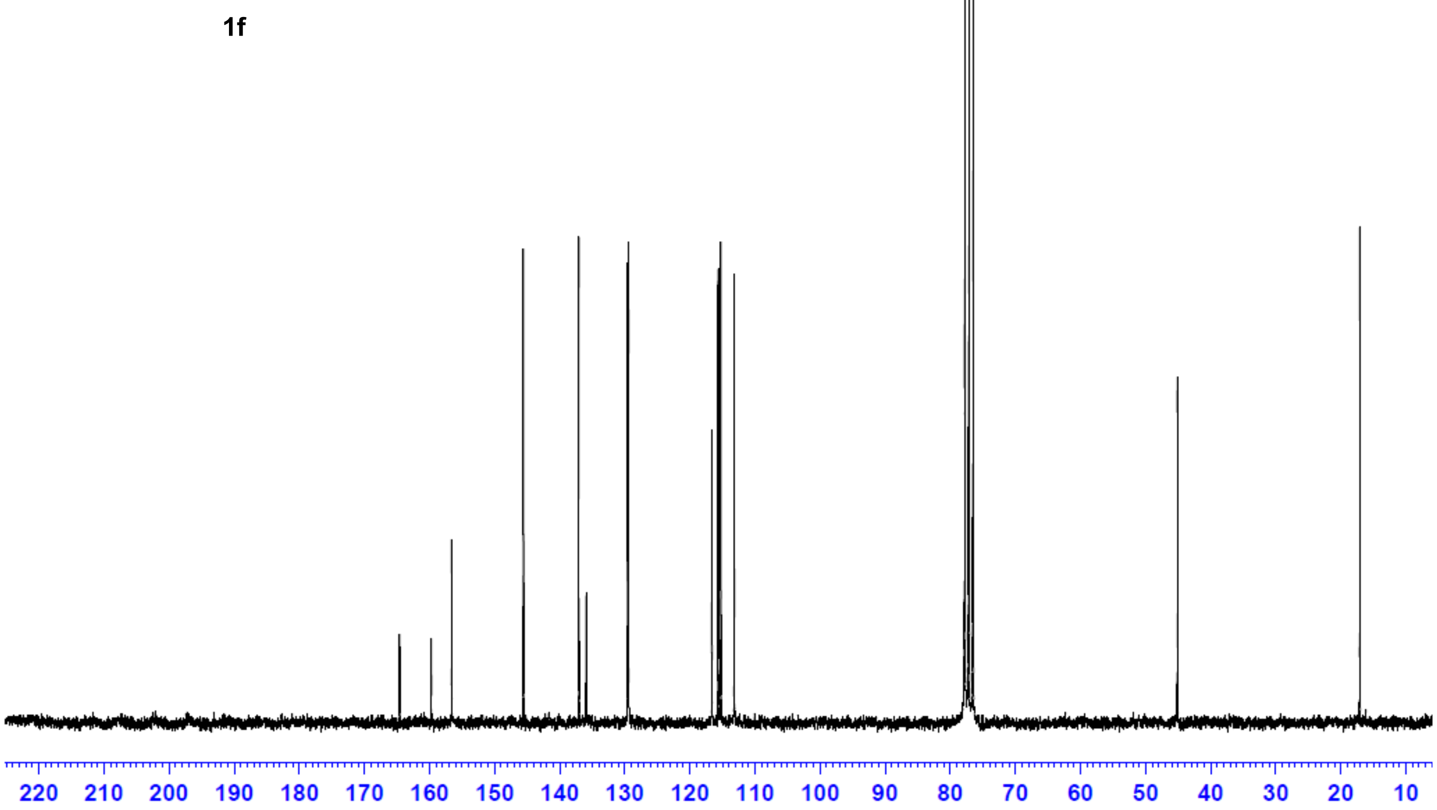
164.59
159.73
156.62
145.59
137.06
135.95
135.89
129.59
129.43
116.63
115.68
115.26
113.20
77.80
77.16
76.53
45.13
17.11
220 210 200 190 180 170 160 150 140 130 120 110 100 90 80 70 60 50 40 30 20 10

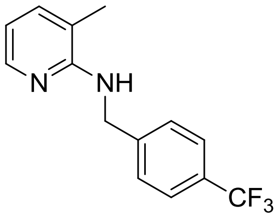

1g

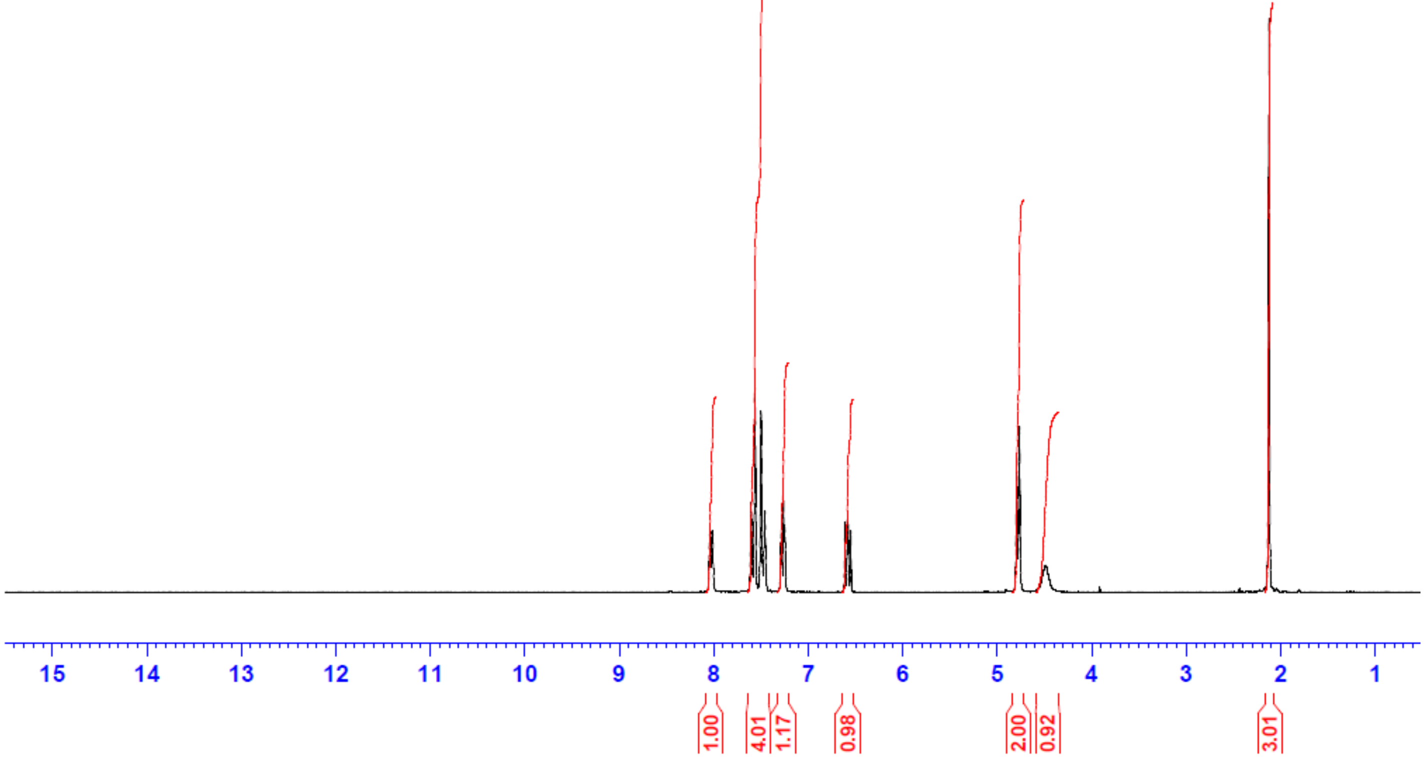

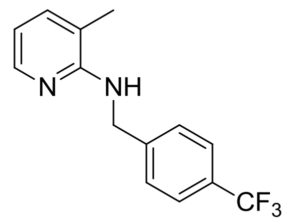
N
NH
CF3

1g

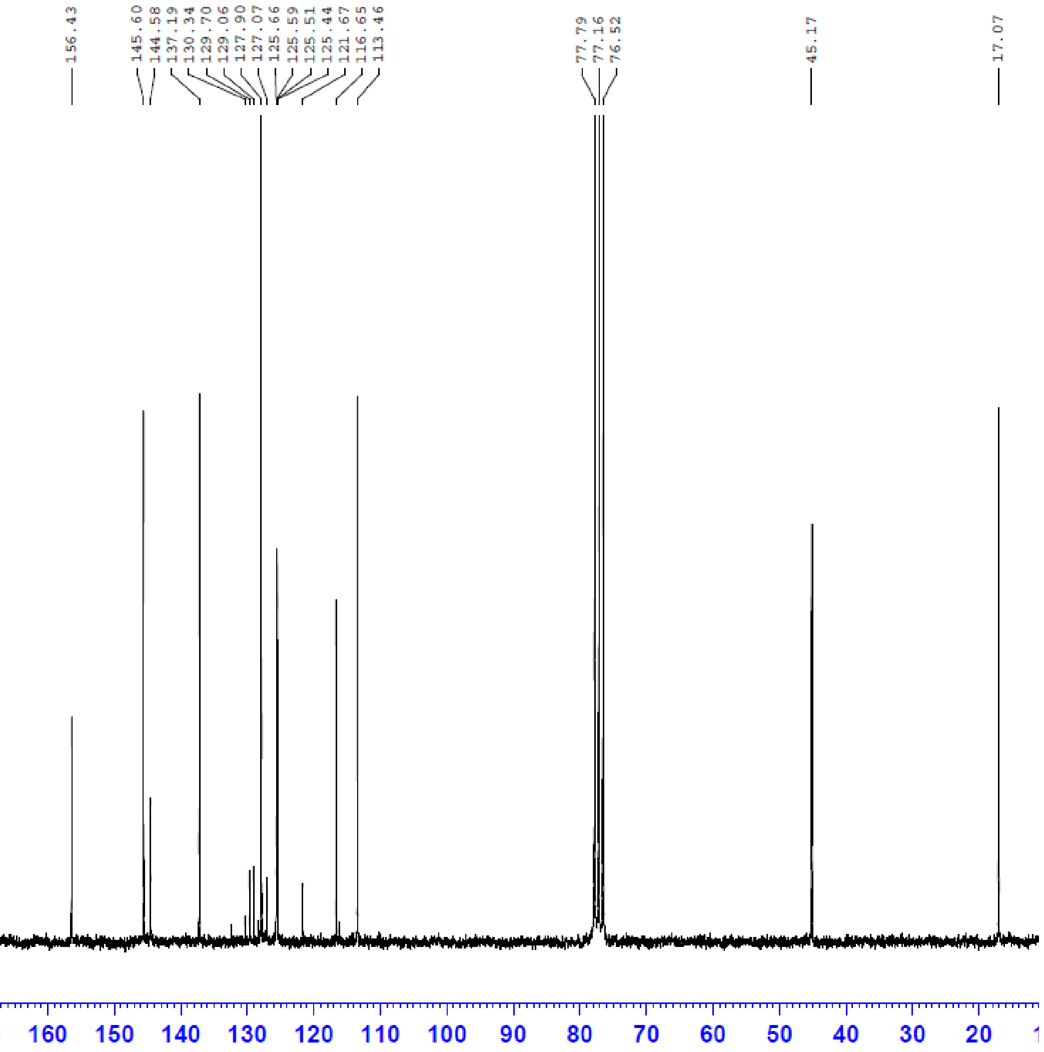
156.43
145.60
144.58
137.19
130.34
129.70
129.06
127.90
127.07
125.66
125.59
125.51
125.44
121.67
116.65
113.46
77.79
77.16
76.52
45.17
17.07
220 210 200 190 180 170 160 150 140 130 120 110 100 90 80 70 60 50 40 30 20

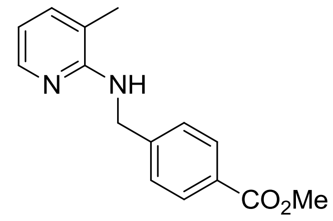

N
NH
CO2Me

1h

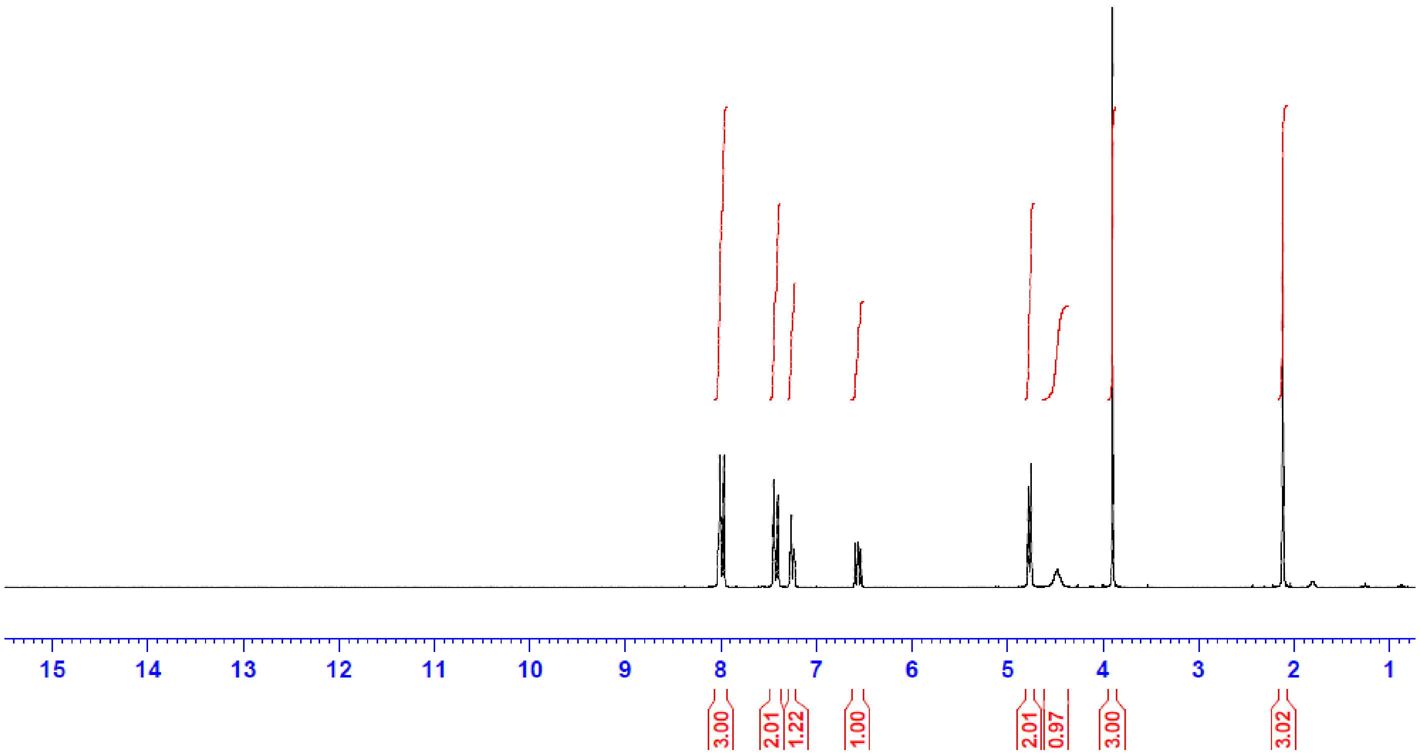

15
14
13
12
11
10
9
8
7
6
5
4
3
2
1
3.00
2.01
1.22
1.00
2.01
0.97
3.00
3.02

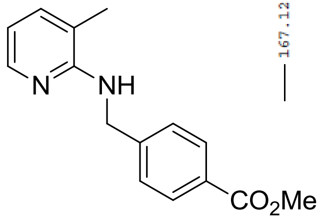

N
NH
CO2Me

1h

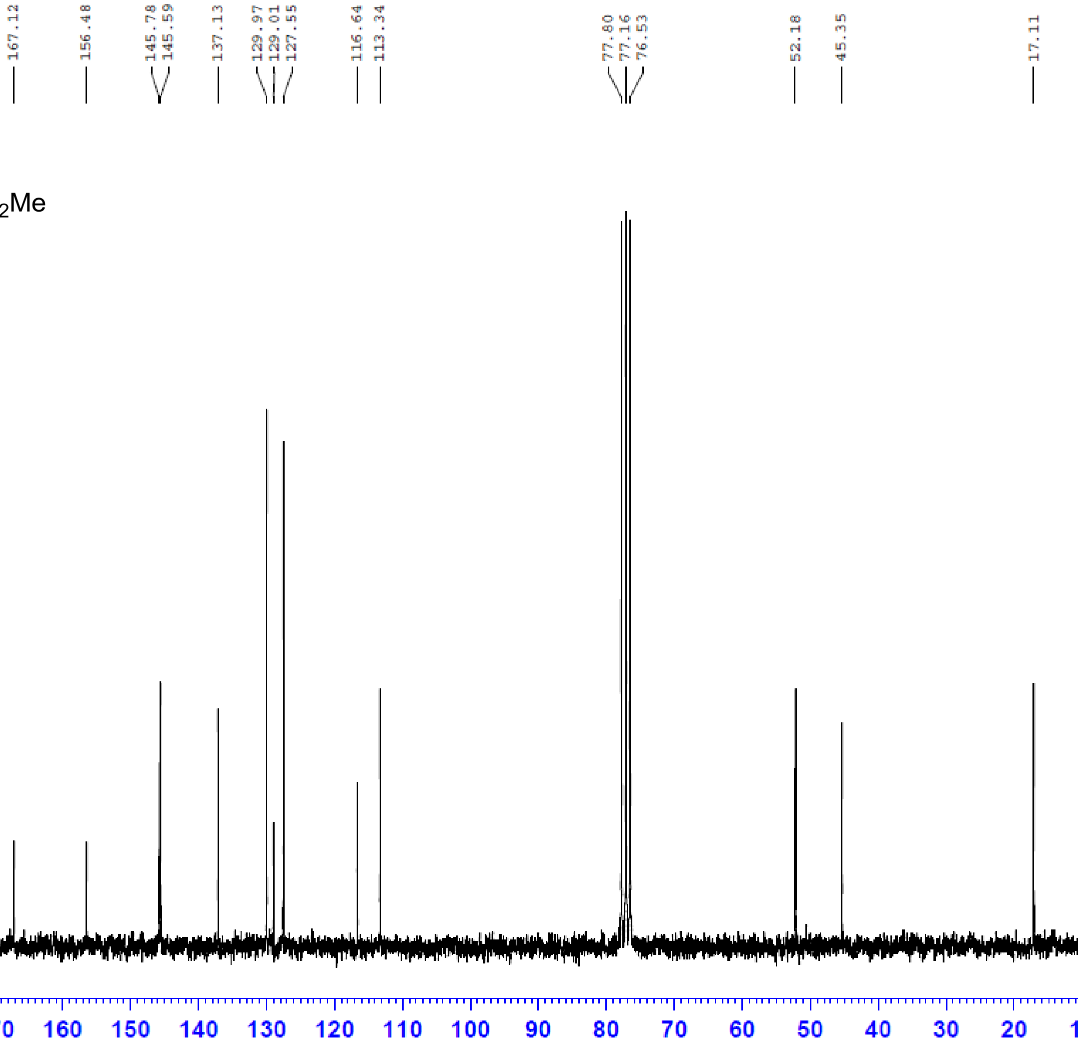

167.12
156.48
145.78
145.59
137.13
129.97
129.01
127.55
116.64
113.34
77.80
77.16
76.53
52.18
45.35
17.11
210 200 190 180 170 160 150 140 130 120 110 100 90 80 70 60 50 40 30 20 1

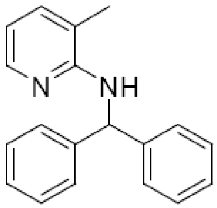

3a

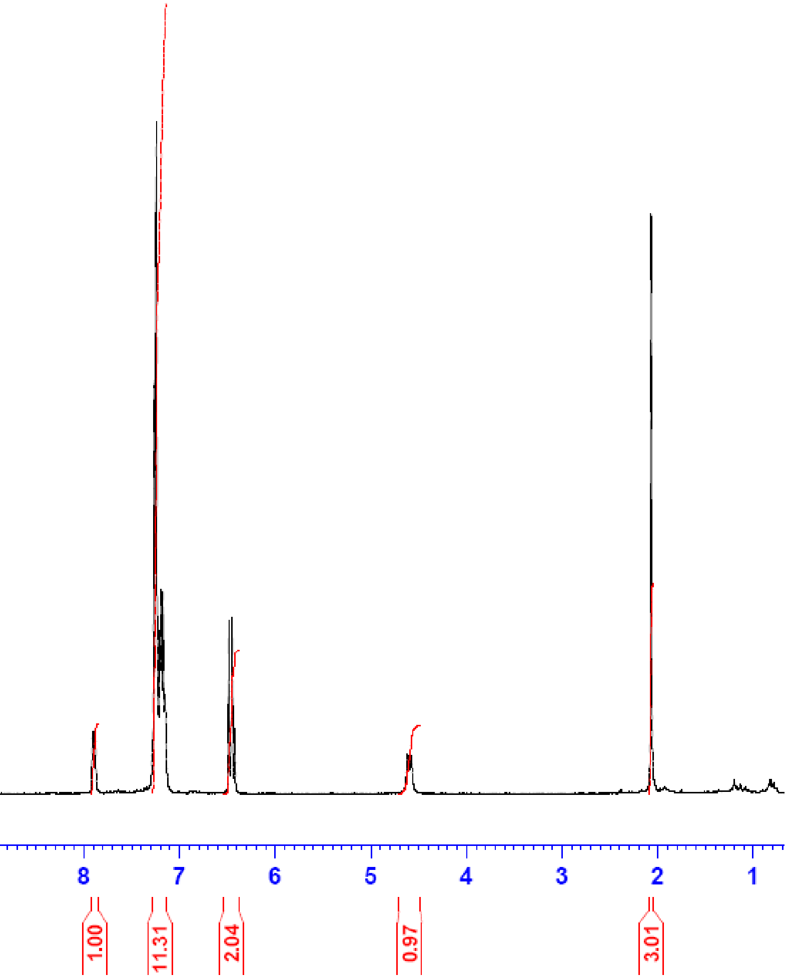

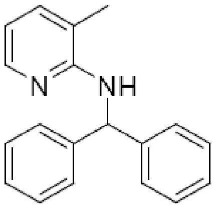
N
NH

3a

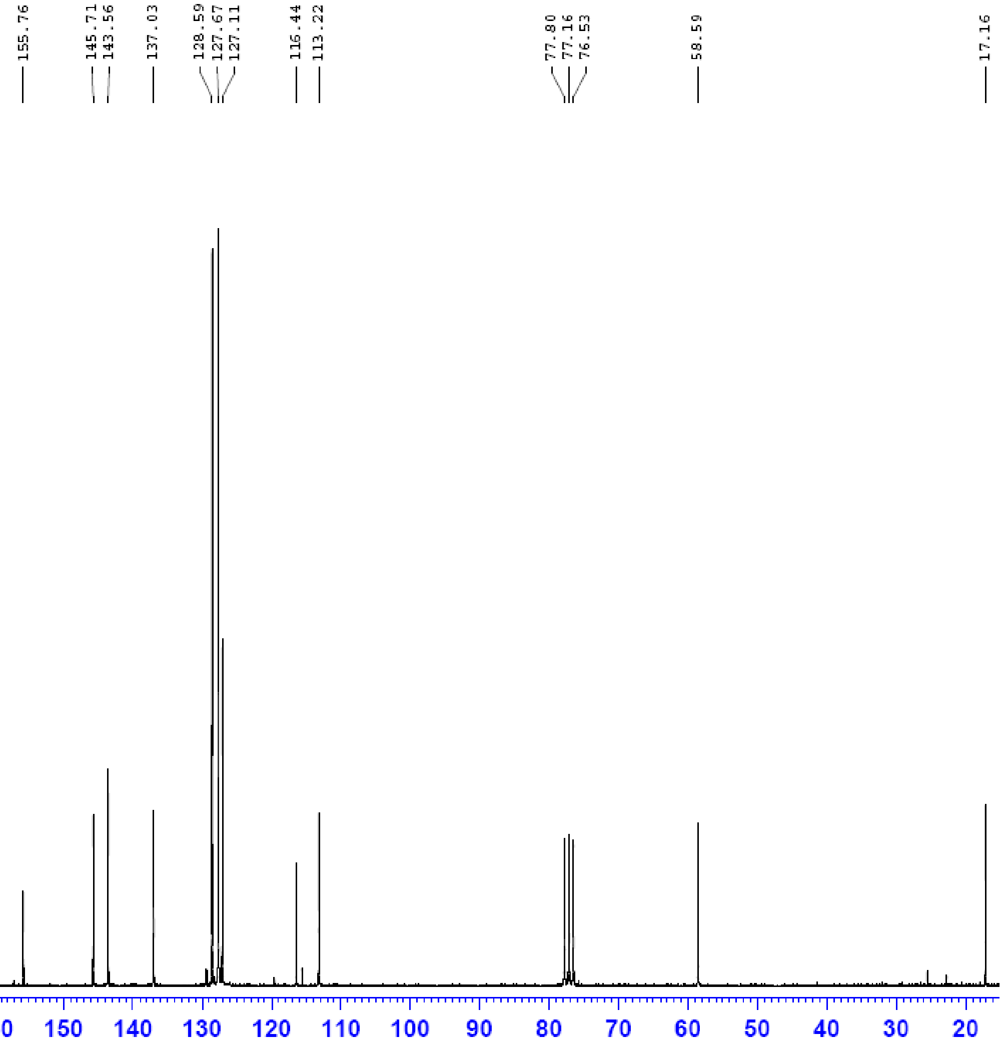
155.76
145.71
143.56
137.03
128.59
127.67
127.11
116.44
113.22
77.80
77.16
76.53
58.59
17.16
210 200 190 180 170 160 150 140 130 120 110 100 90 80 70 60 50 40 30 20

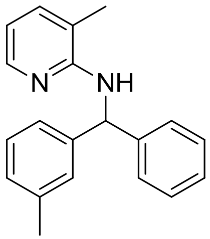

3d

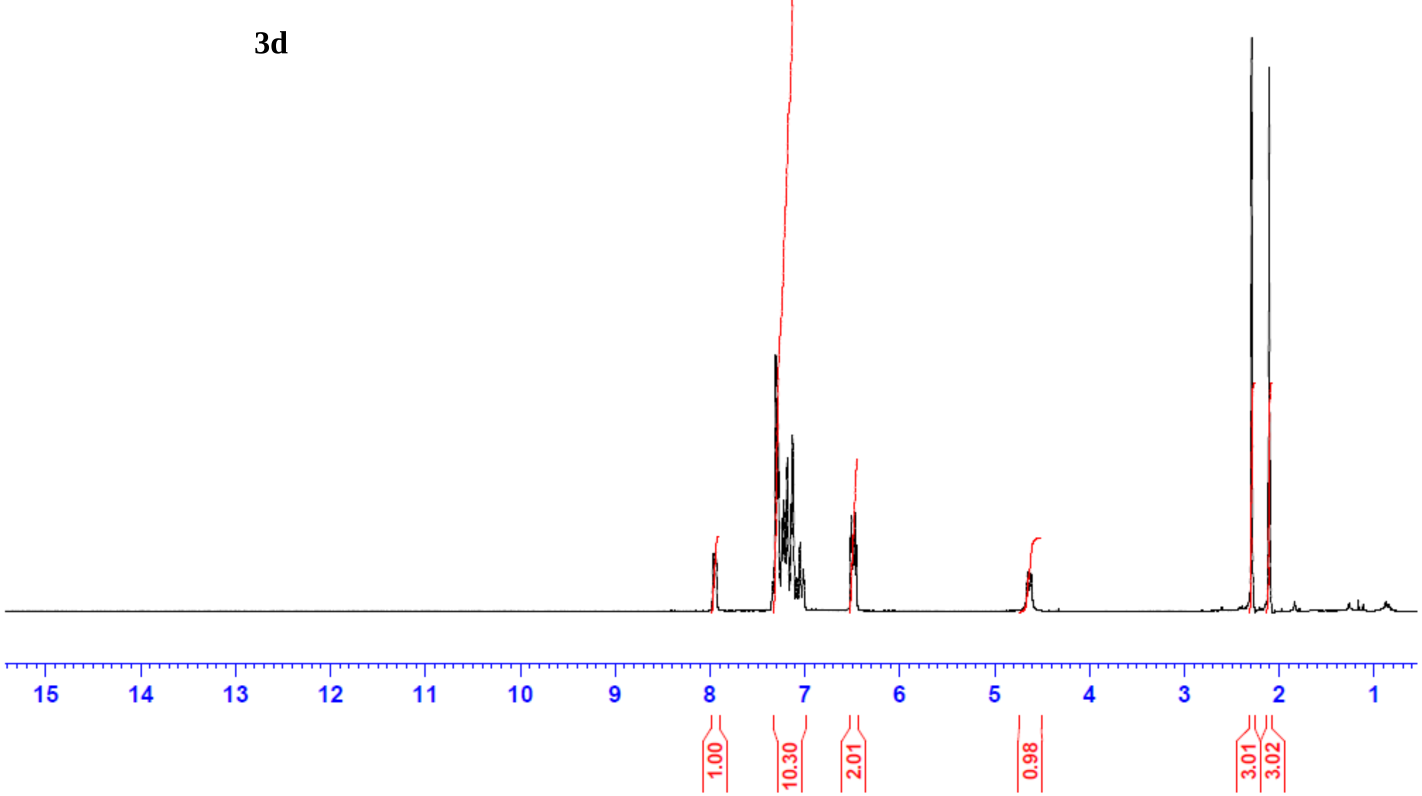

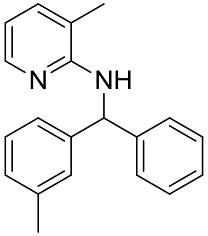
N
NH

3d

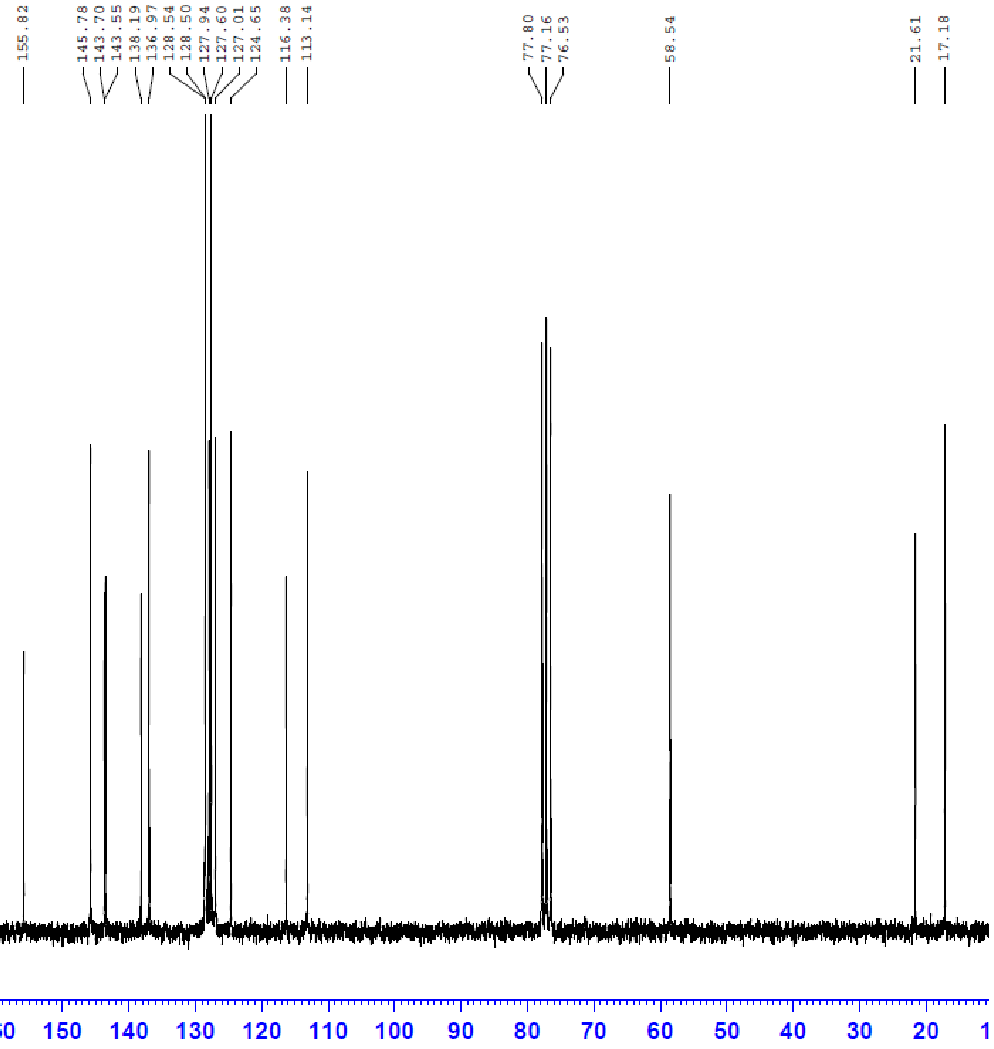
155.82
145.78
143.70
143.55
138.19
136.97
128.54
128.50
127.94
127.60
127.01
124.65
116.38
113.14
77.80
77.16
76.53
58.54
21.61
17.18
220 210 200 190 180 170 160 150 140 130 120 110 100 90 80 70 60 50 40 30 20 1

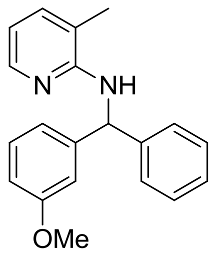
N
NH
OMe

3e

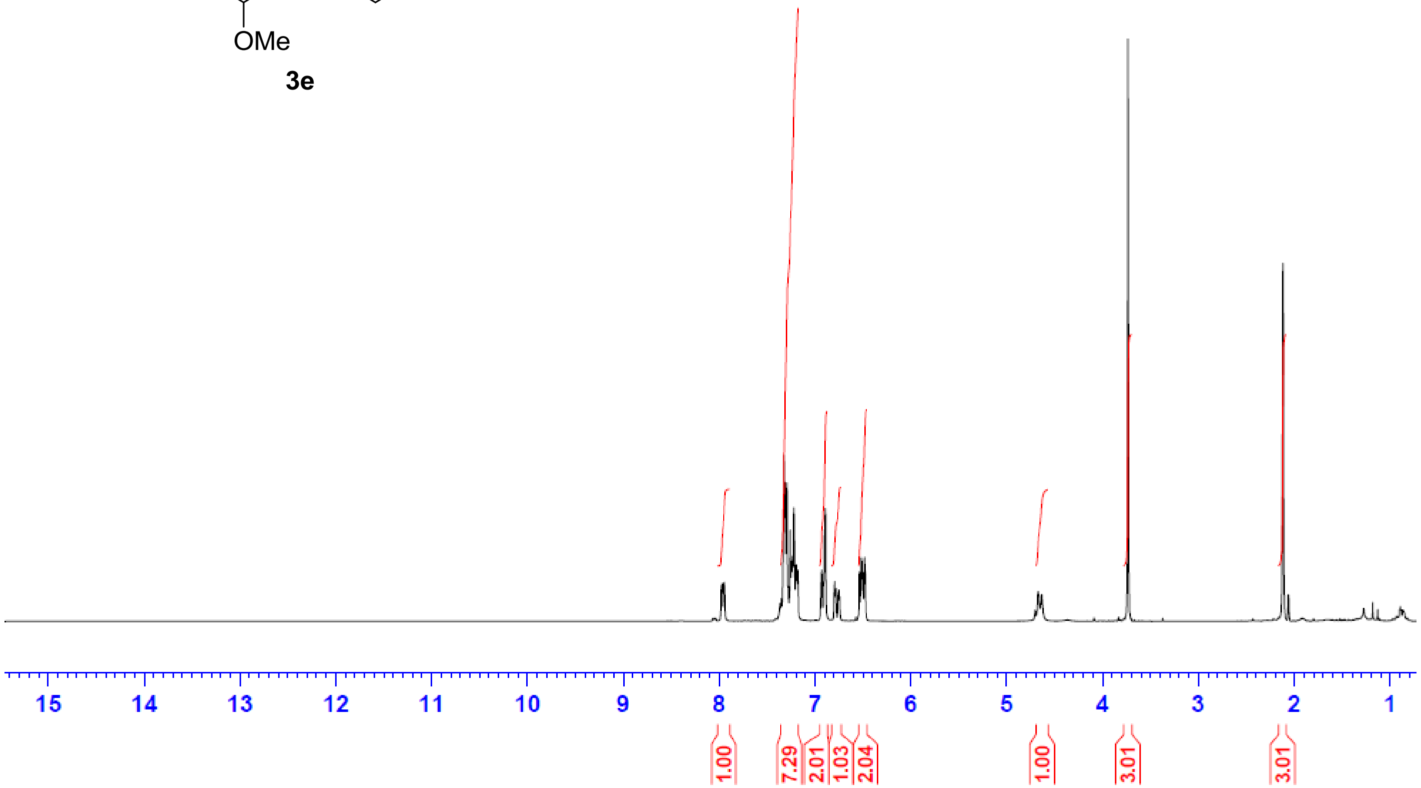
15
14
13
12
11
10
9
8
7
6
5
4
3
2
1
1.00
7.29
2.01
1.03
2.04
1.00
3.01
3.01

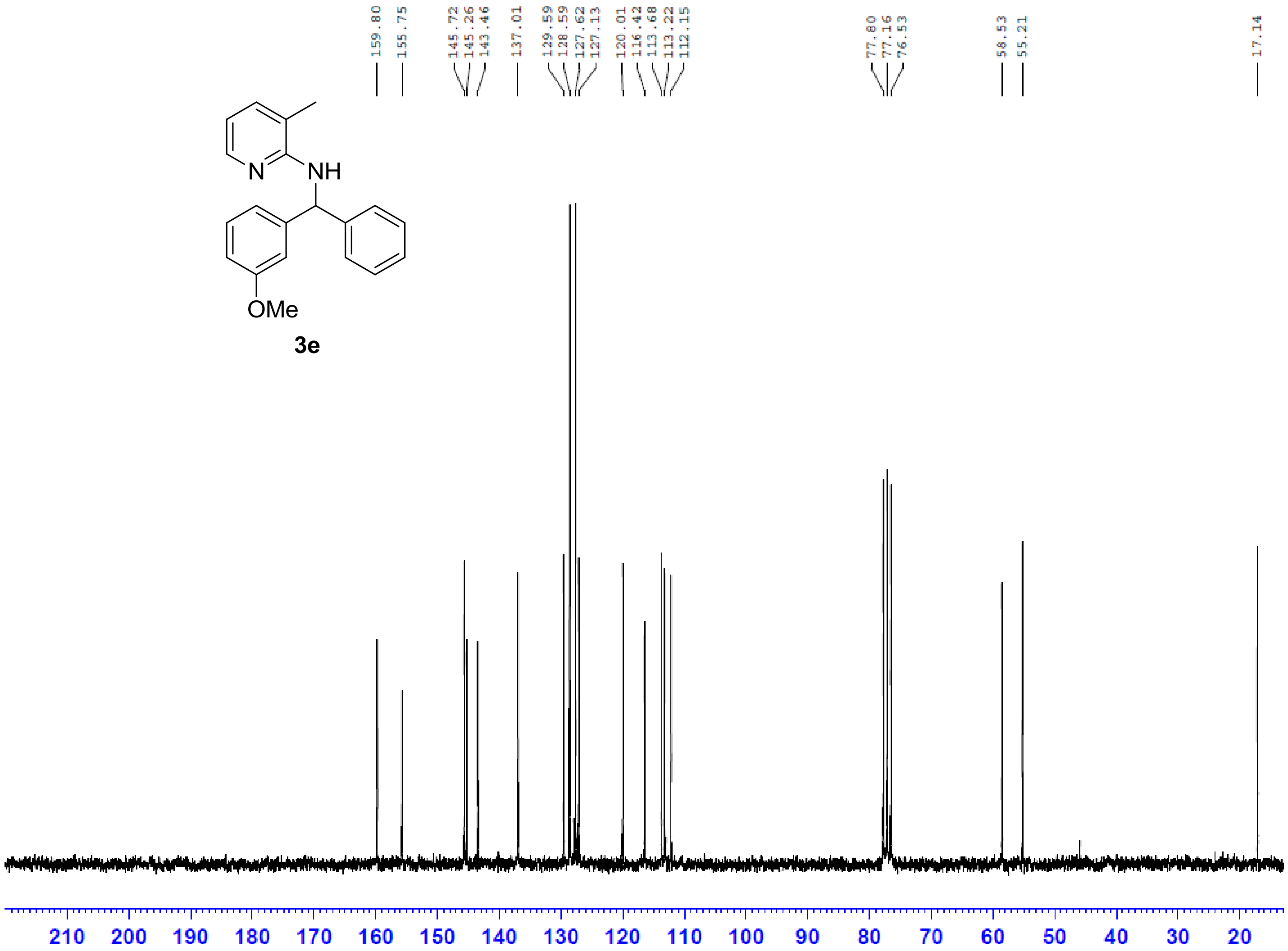

159.80
155.75
145.72
145.26
143.46
137.01
129.59
128.59
127.62
127.13
120.01
116.42
113.68
113.22
112.15
77.80
77.16
76.53
58.53
55.21
17.14
NH
N
OMe
3e
210 200 190 180 170 160 150 140 130 120 110 100 90 80 70 60 50 40 30 20

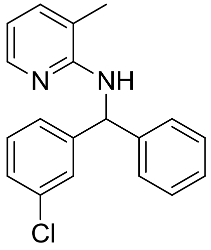

3f

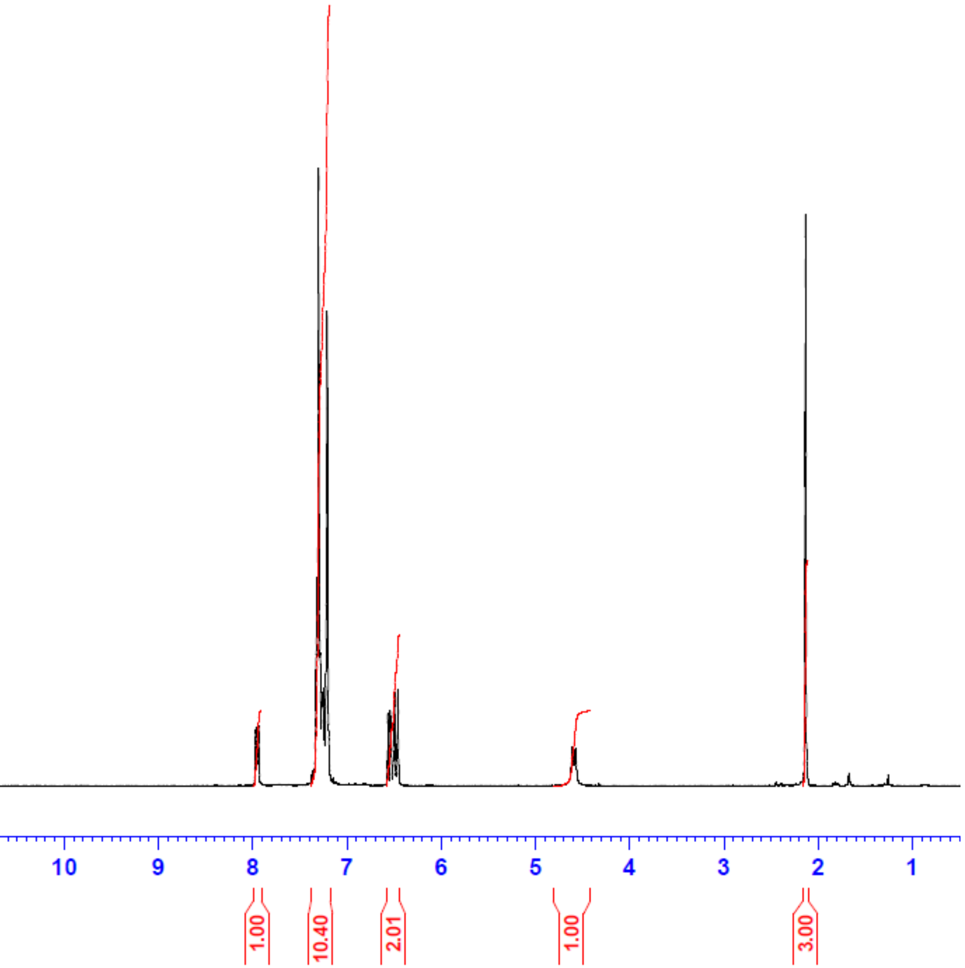

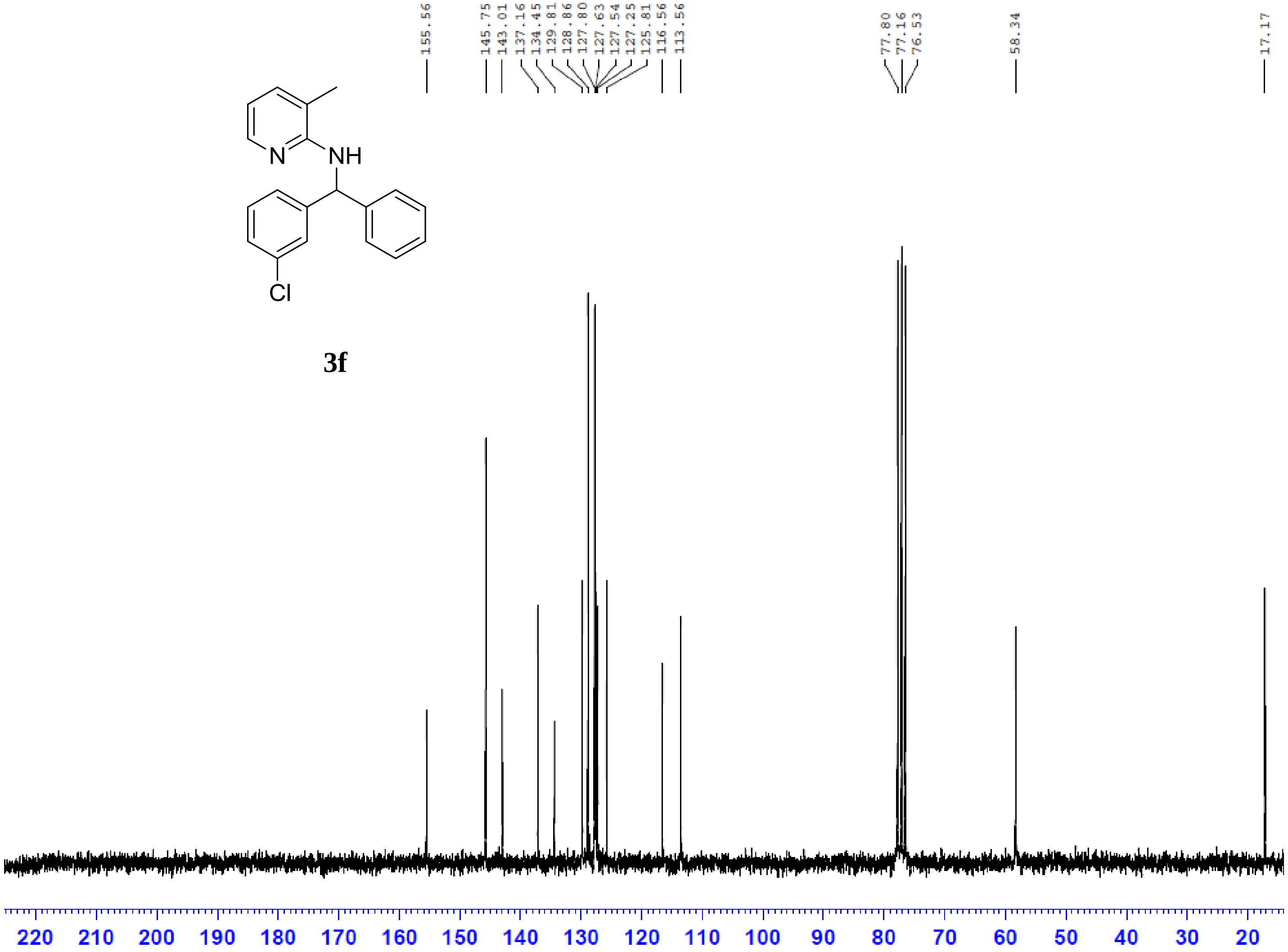
155.56
145.75
143.01
137.16
134.45
129.81
128.86
127.80
127.63
127.54
127.25
125.81
116.56
113.56
77.80
77.16
76.53
58.34
17.17
N
NH
Cl
3f
220 210 200 190 180 170 160 150 140 130 120 110 100 90 80 70 60 50 40 30 20

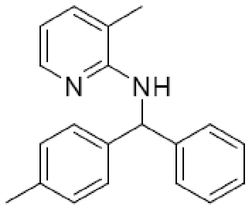

3g

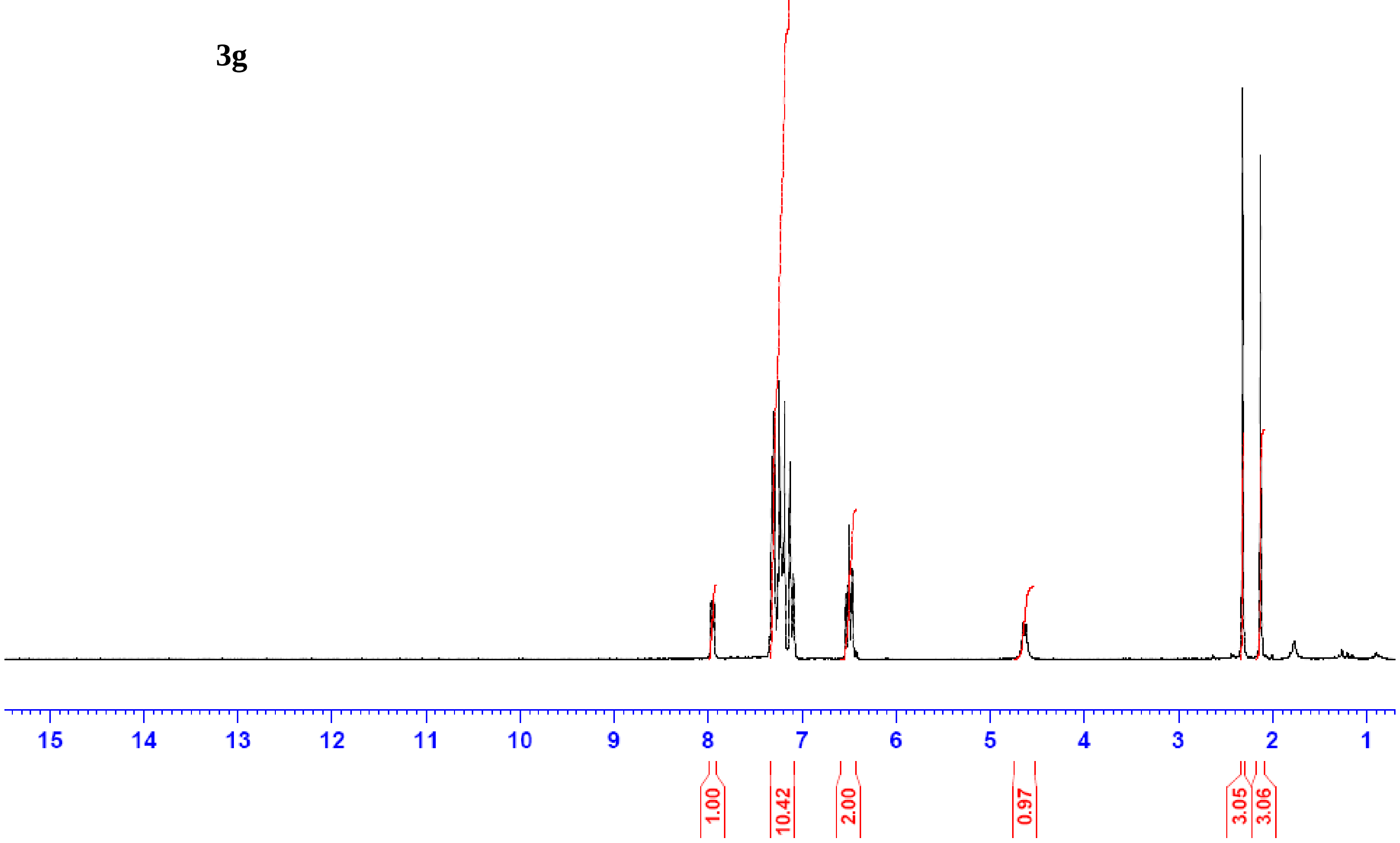

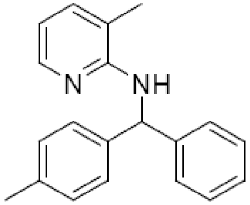
N
NH

3g

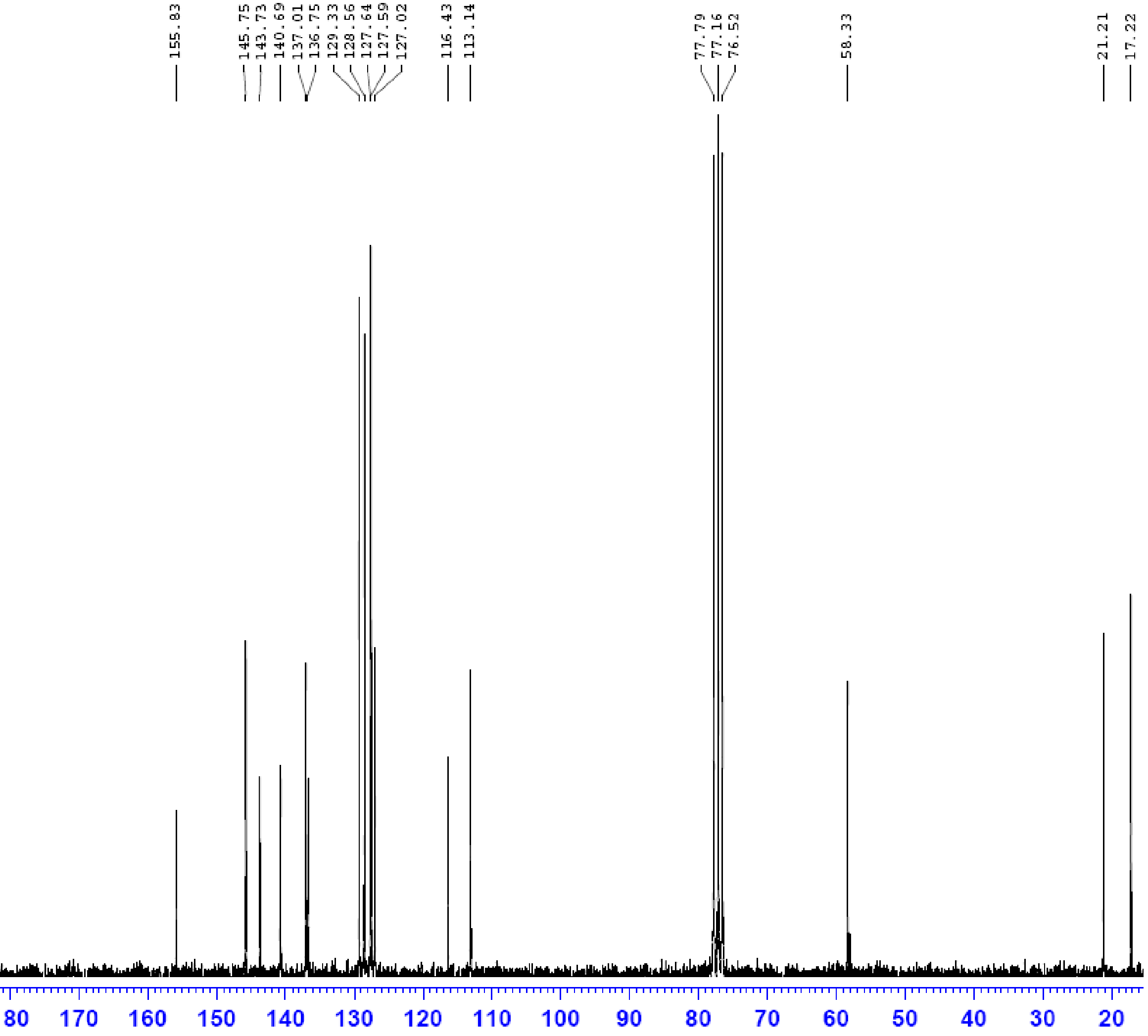
155.83
145.75
143.73
140.69
137.01
136.75
129.33
128.56
127.64
127.59
127.02
116.43
113.14
77.79
77.16
76.52
58.33
21.21
17.22
210 200 190 180 170 160 150 140 130 120 110 100 90 80 70 60 50 40 30 20

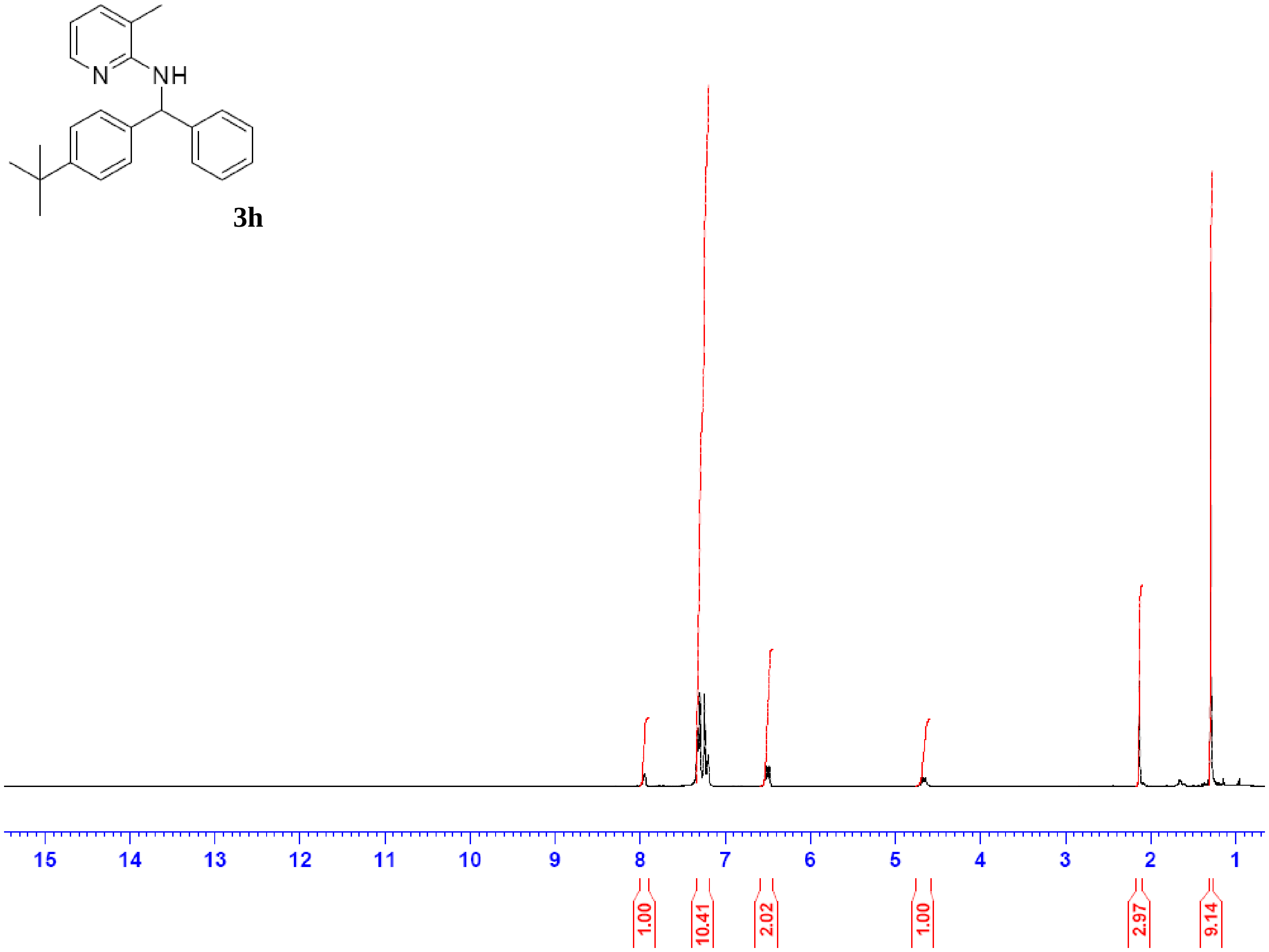

3h

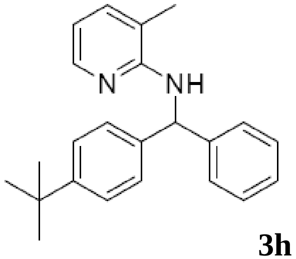

3h

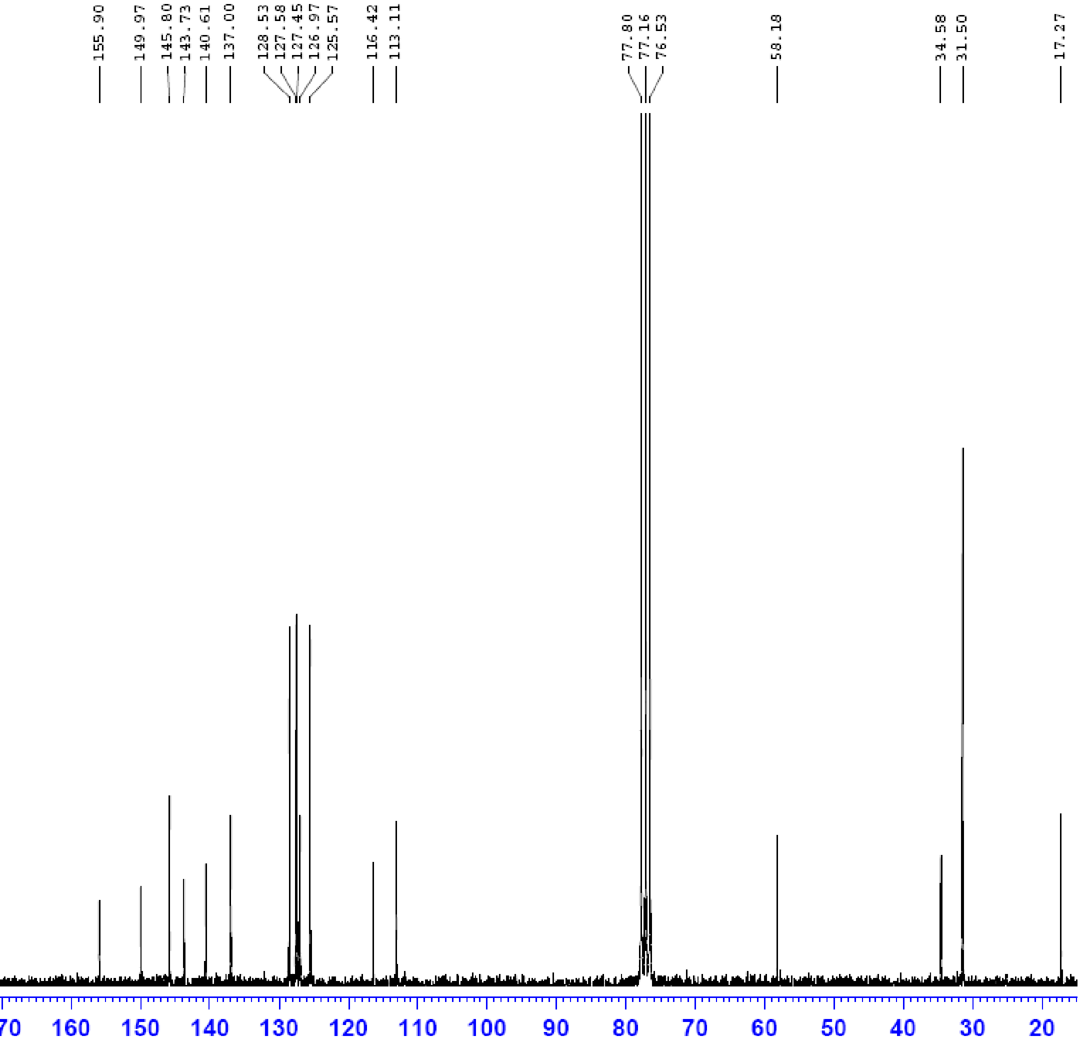
155.90
149.97
145.80
143.73
140.61
137.00
128.53
127.58
127.45
126.97
125.57
116.42
113.11
77.80
77.16
76.53
58.18
34.58
31.50
17.27
210 200 190 180 170 160 150 140 130 120 110 100 90 80 70 60 50 40 30 20

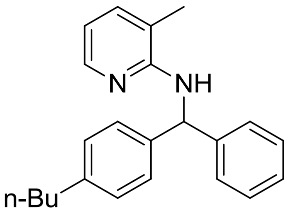

NH
N
n-Bu

3i

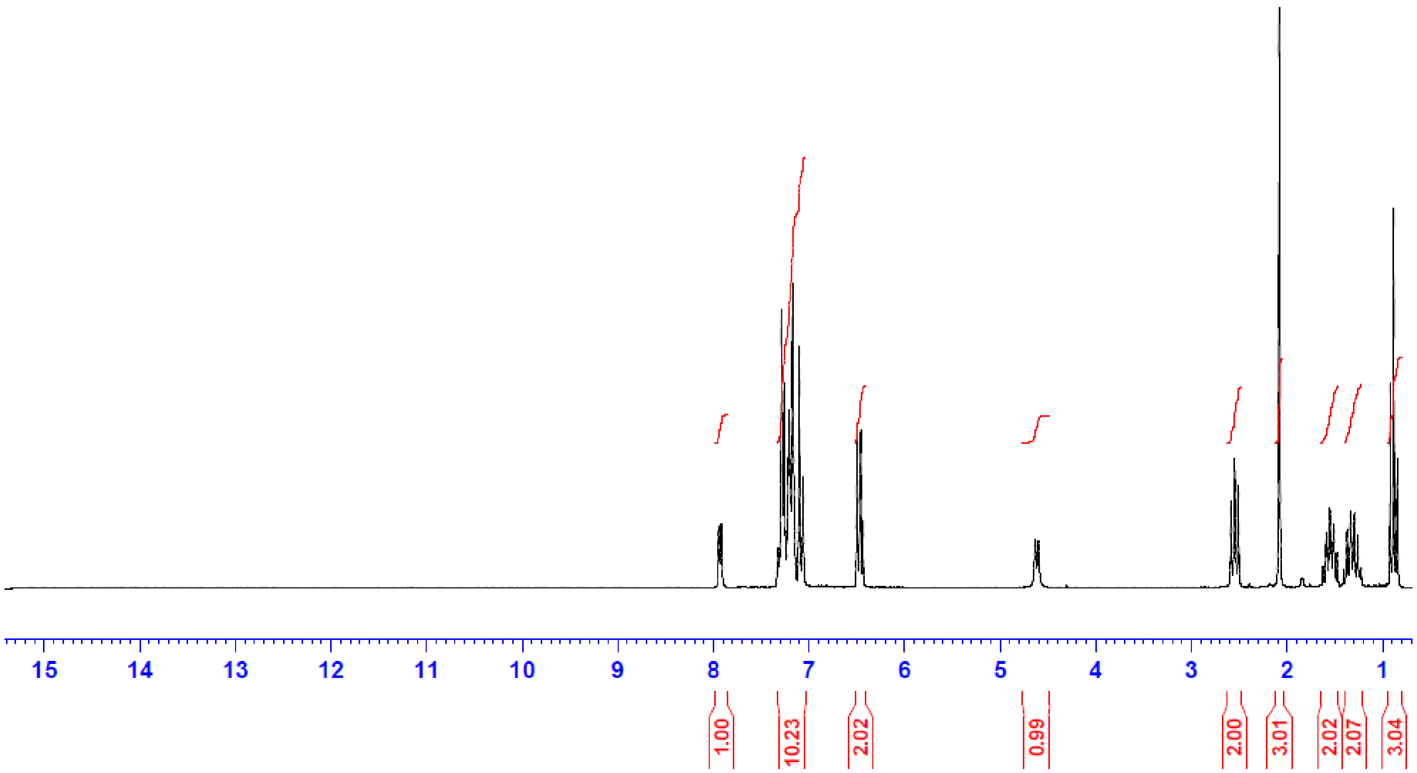

15
14
13
12
11
10
9
8
7
6
5
4
3
2
1
1.00
10.23
2.02
0.99
2.00
3.01
2.02
2.07
3.04

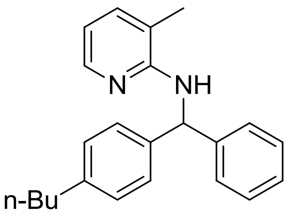

3i

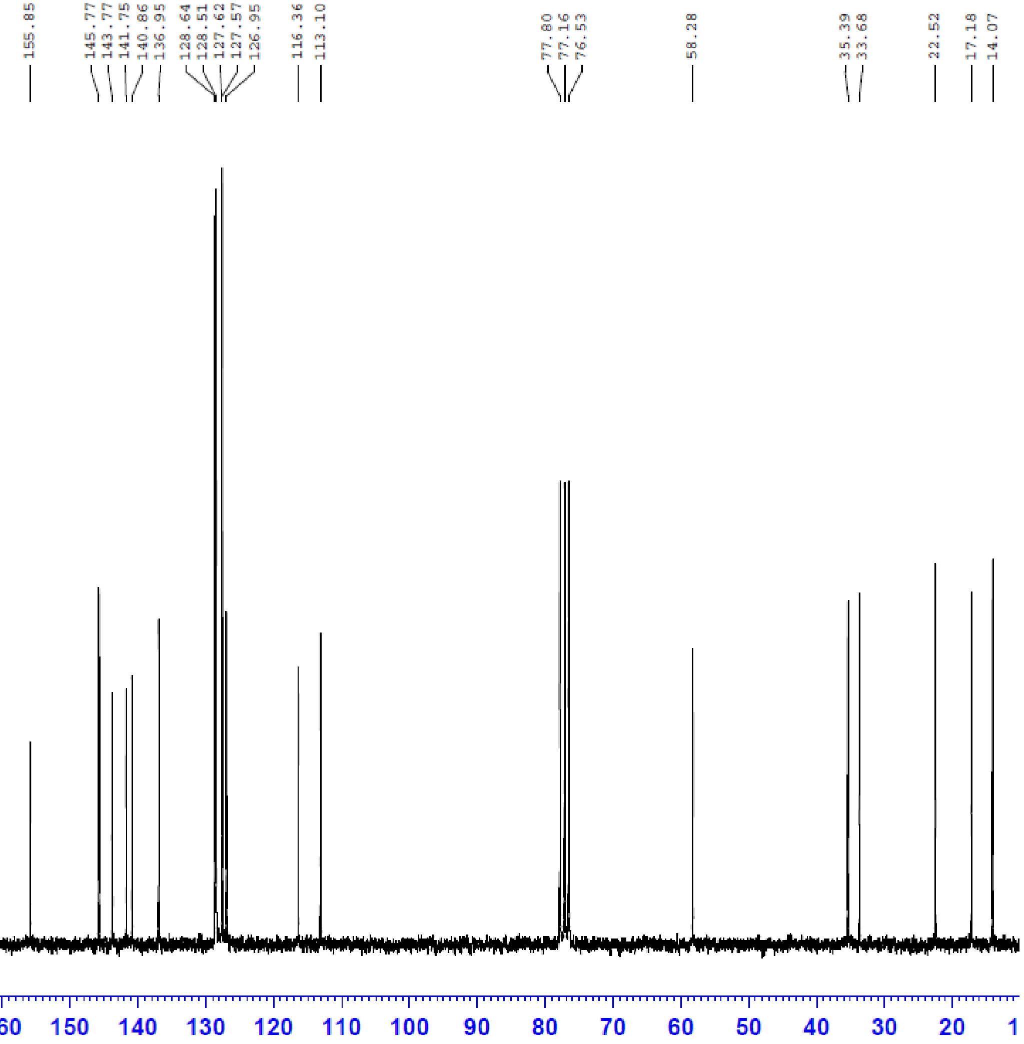
155.85
145.77
143.77
141.75
140.86
136.95
128.64
128.51
127.62
127.57
126.95
116.36
113.10
77.80
77.16
76.53
58.28
35.39
33.68
22.52
17.18
14.07
210 200 190 180 170 160 150 140 130 120 110 100 90 80 70 60 50 40 30 20 1

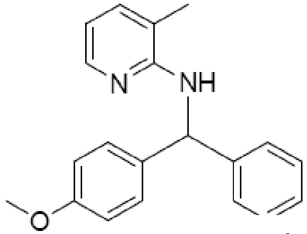

3j

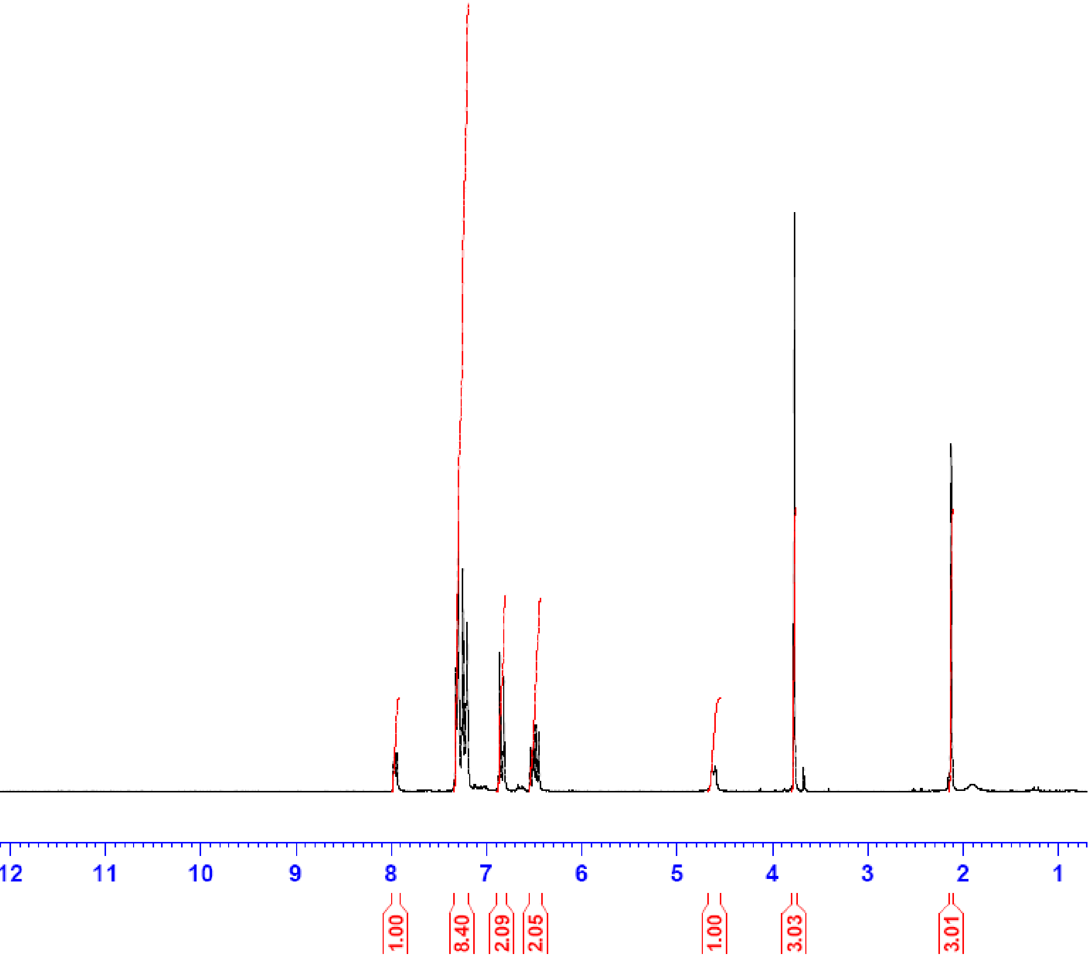

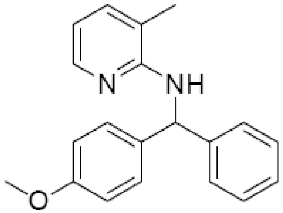

3j

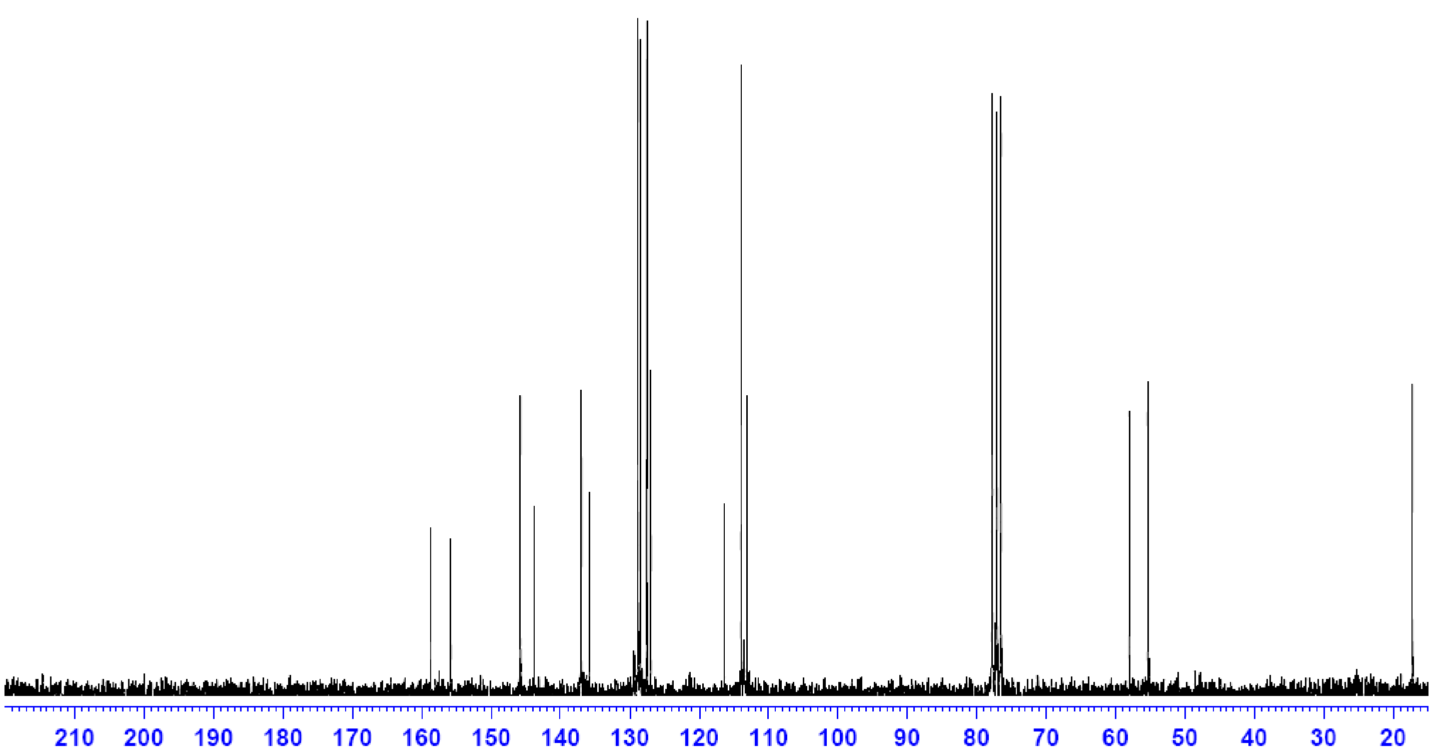
158.72
155.83
145.76
143.80
137.02
135.84
128.87
128.56
127.56
127.02
116.43
113.99
113.15
77.79
77.16
76.52
58.01
55.36
17.23
210 200 190 180 170 160 150 140 130 120 110 100 90 80 70 60 50 40 30 20

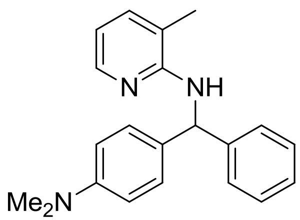

3k

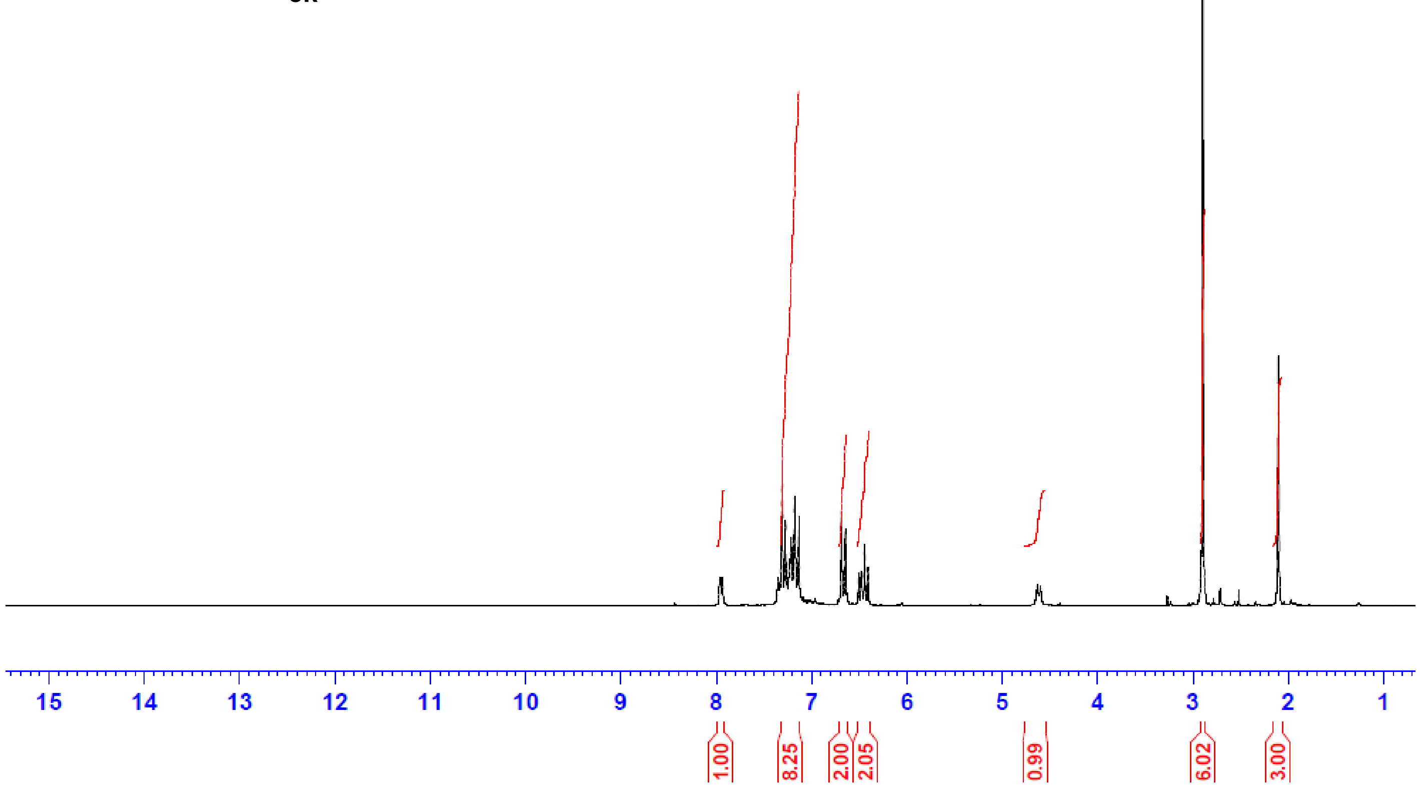

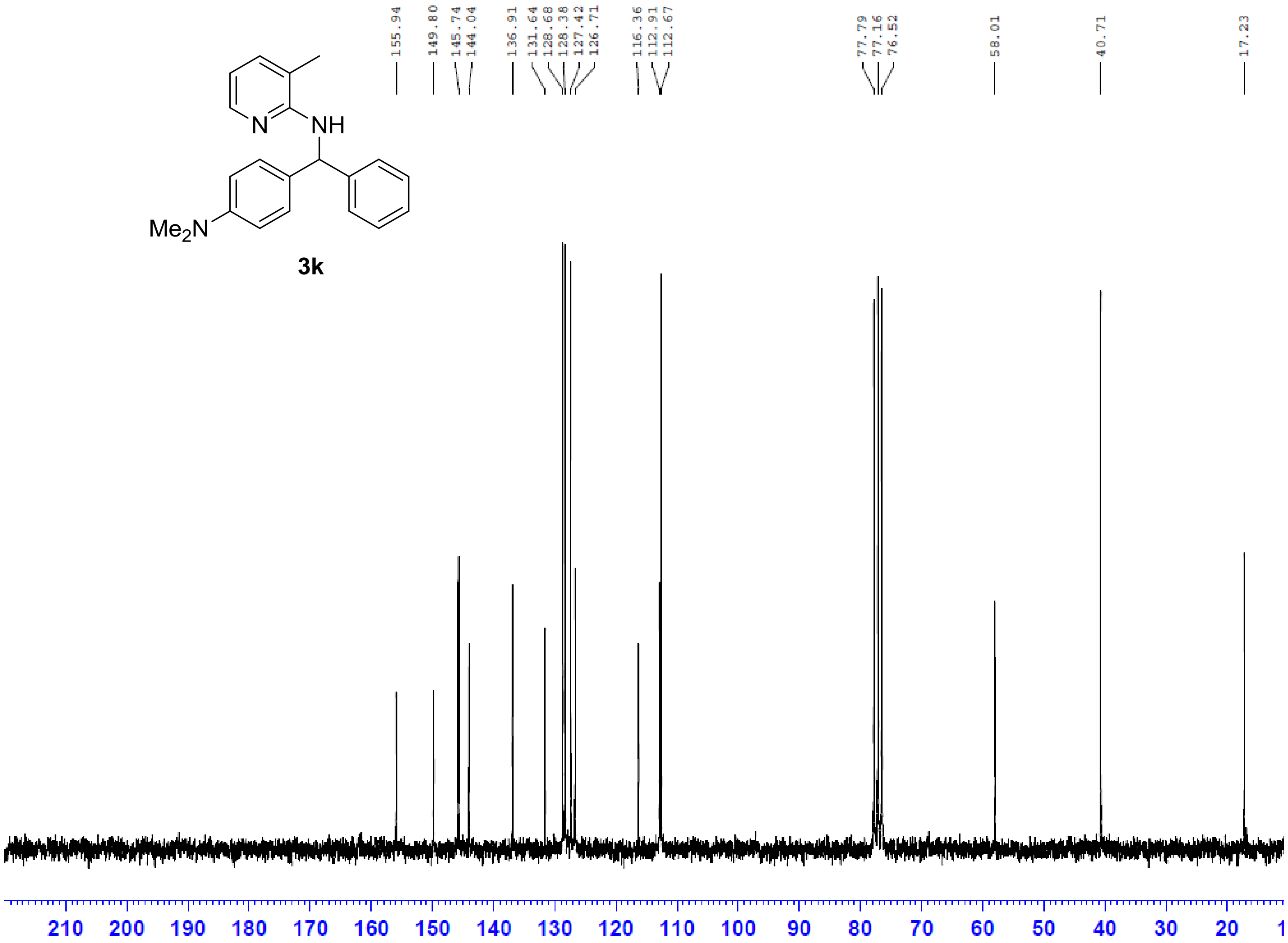

155.94
149.80
145.74
144.04
136.91
131.64
128.68
128.38
127.42
126.71
116.36
112.91
112.67
77.79
77.16
76.52
58.01
40.71
17.23
NH
N
Me2N
210 200 190 180 170 160 150 140 130 120 110 100 90 80 70 60 50 40 30 20

3k

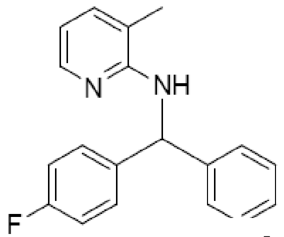

3l

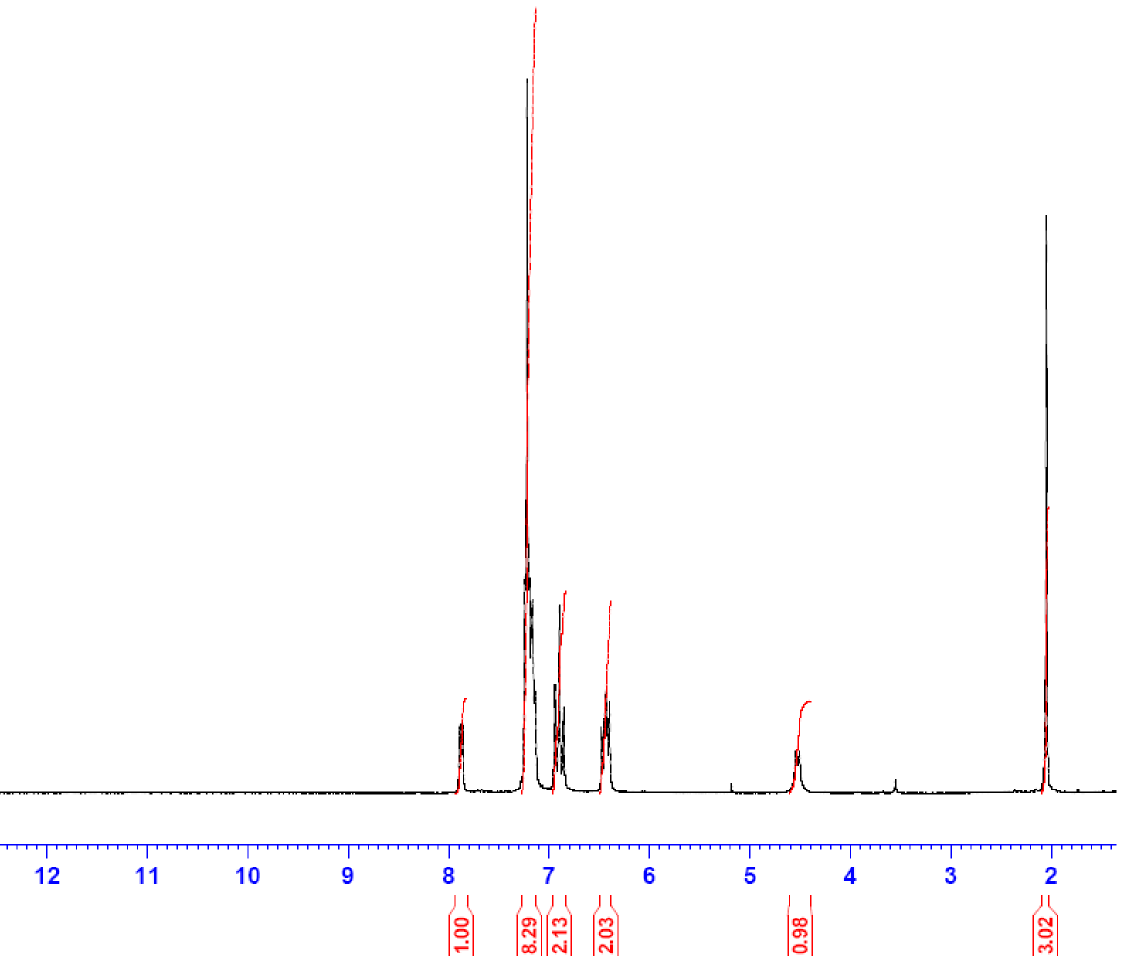

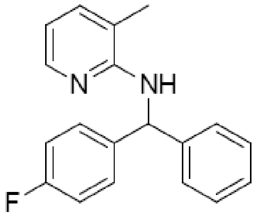
N
NH
F

3l

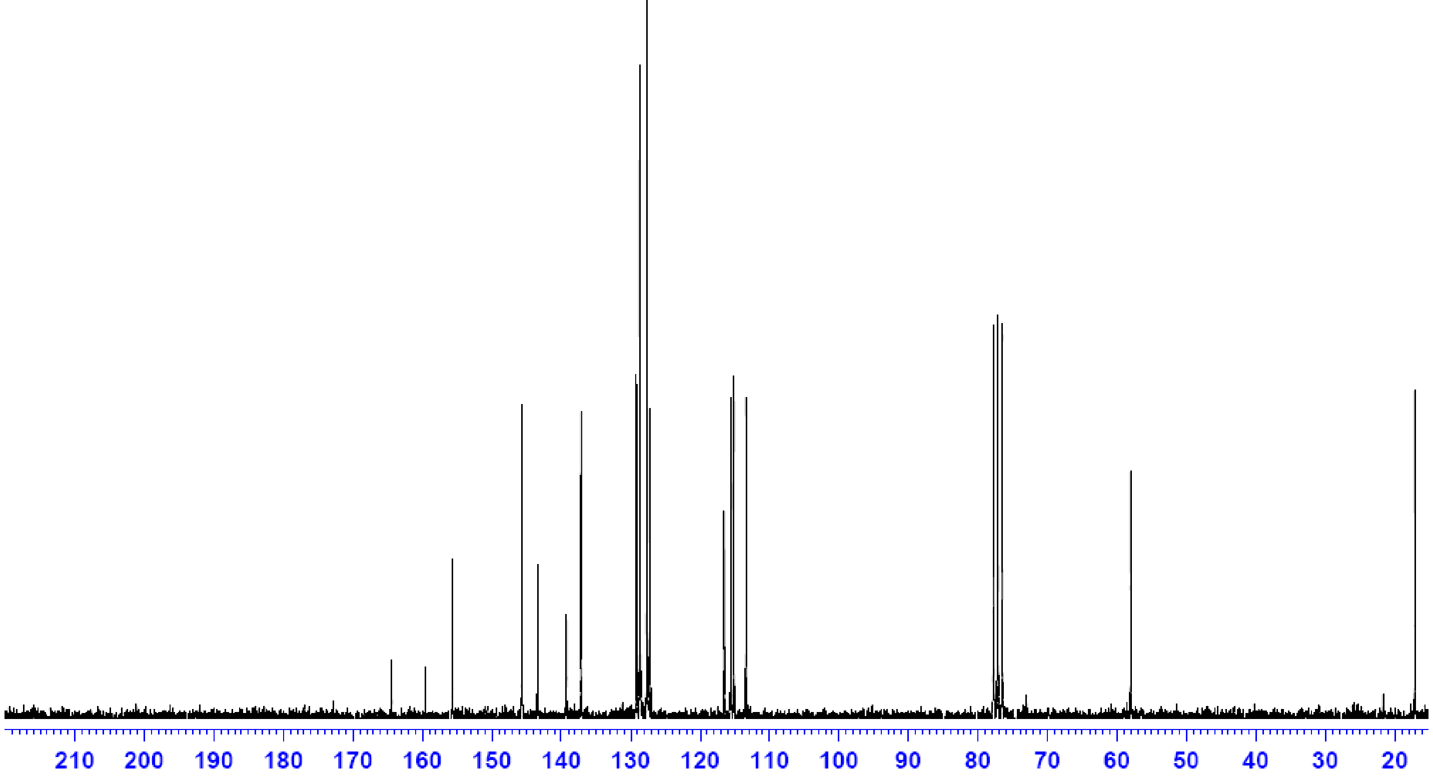
164.38
159.51
155.63
145.66
143.40
139.31
139.25
137.15
129.26
129.10
128.74
127.69
127.34
127.13
116.53
115.57
115.15
113.41
77.80
77.16
76.53
58.03
17.16
210 200 190 180 170 160 150 140 130 120 110 100 90 80 70 60 50 40 30 20

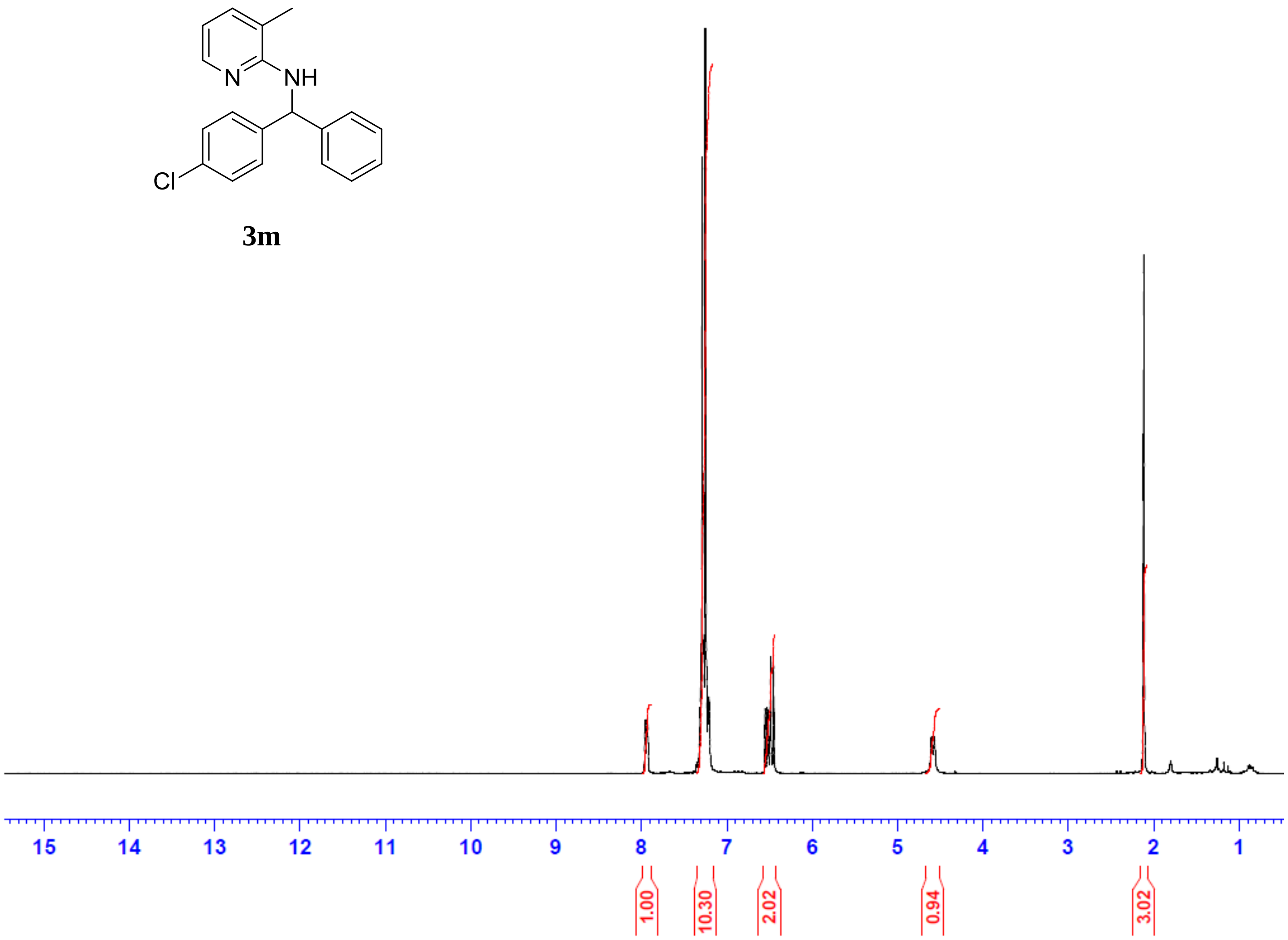

3m

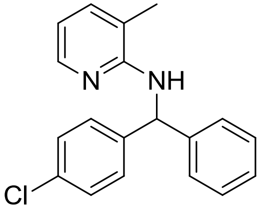
NH
N
Cl

3m

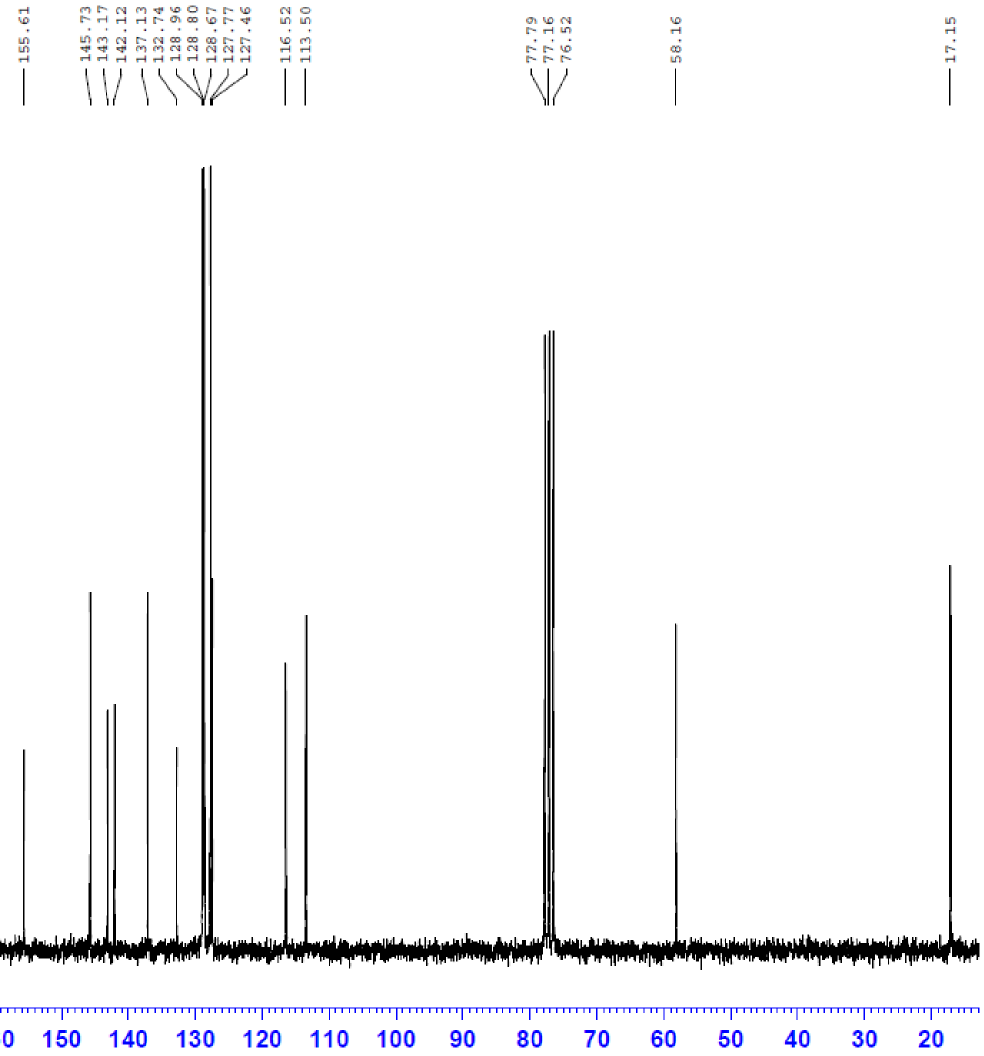
155.61
145.73
143.17
142.12
137.13
132.74
128.96
128.80
128.67
127.77
127.46
116.52
113.50
77.79
77.16
76.52
58.16
17.15
220 210 200 190 180 170 160 150 140 130 120 110 100 90 80 70 60 50 40 30 20

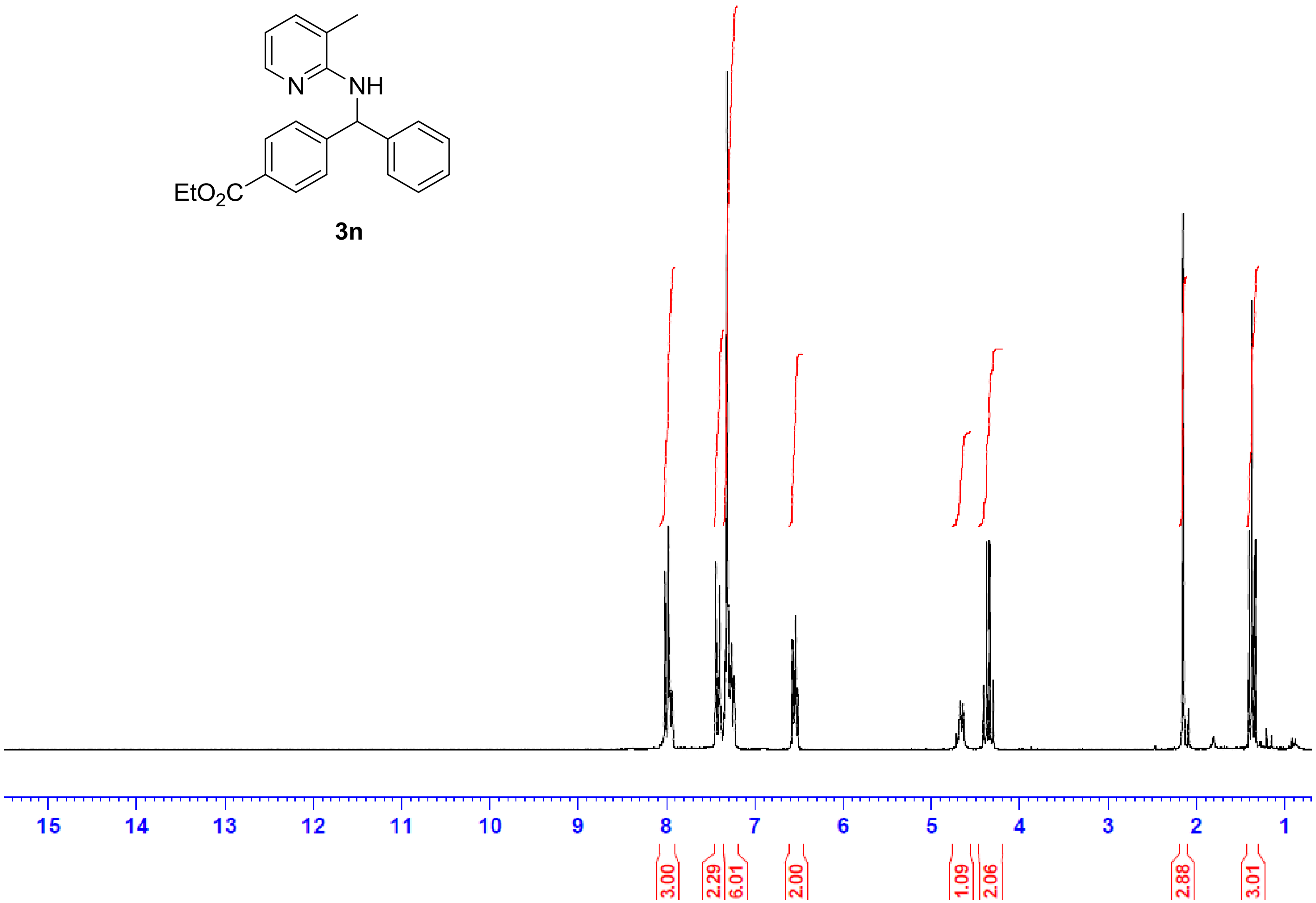
N
NH
EtO2C
3n
15
14
13
12
11
10
9
8
7
6
5
4
3
2
1
3.00
2.29
6.01
2.00
1.09
2.06
2.88
3.01

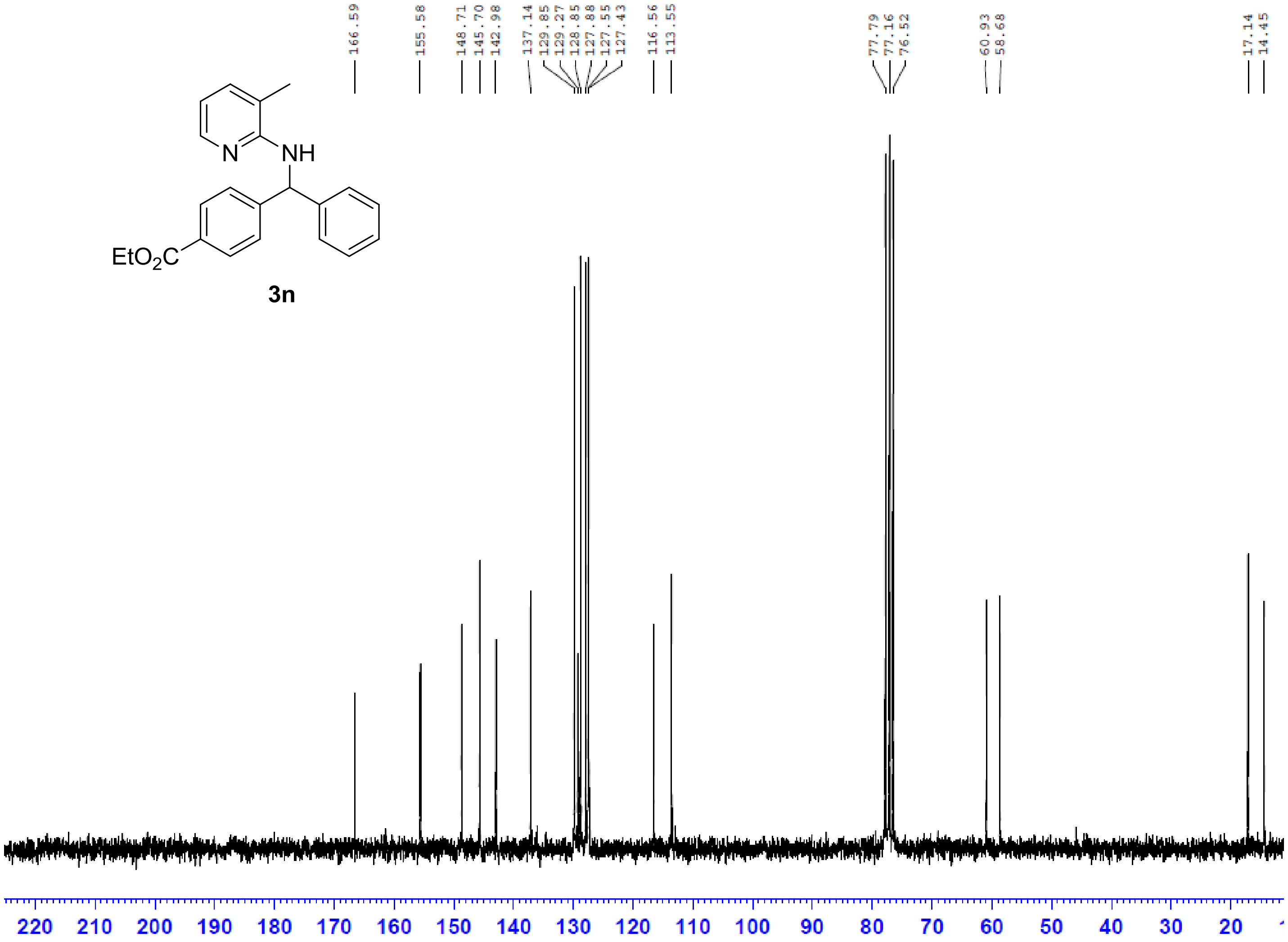
EtO2C
NH
N
166.59
155.58
148.71
145.70
142.98
137.14
129.85
129.27
128.85
127.88
127.55
127.43
116.56
113.55
77.79
77.16
76.52
60.93
58.68
17.14
14.45
220 210 200 190 180 170 160 150 140 130 120 110 100 90 80 70 60 50 40 30 20

3n

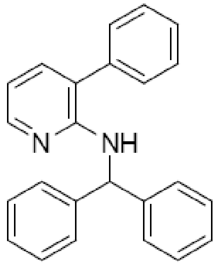

3t

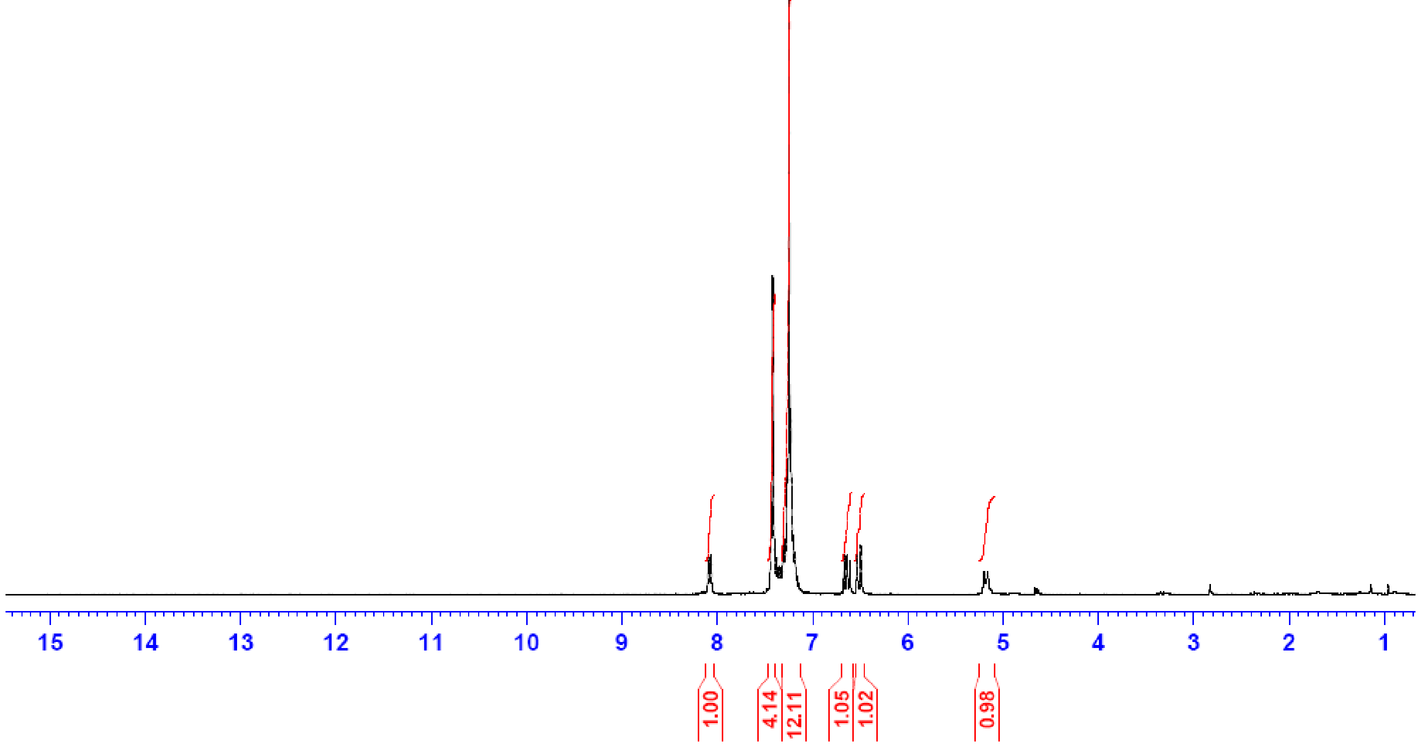

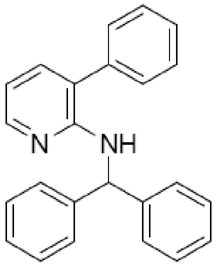
N
NH

3t

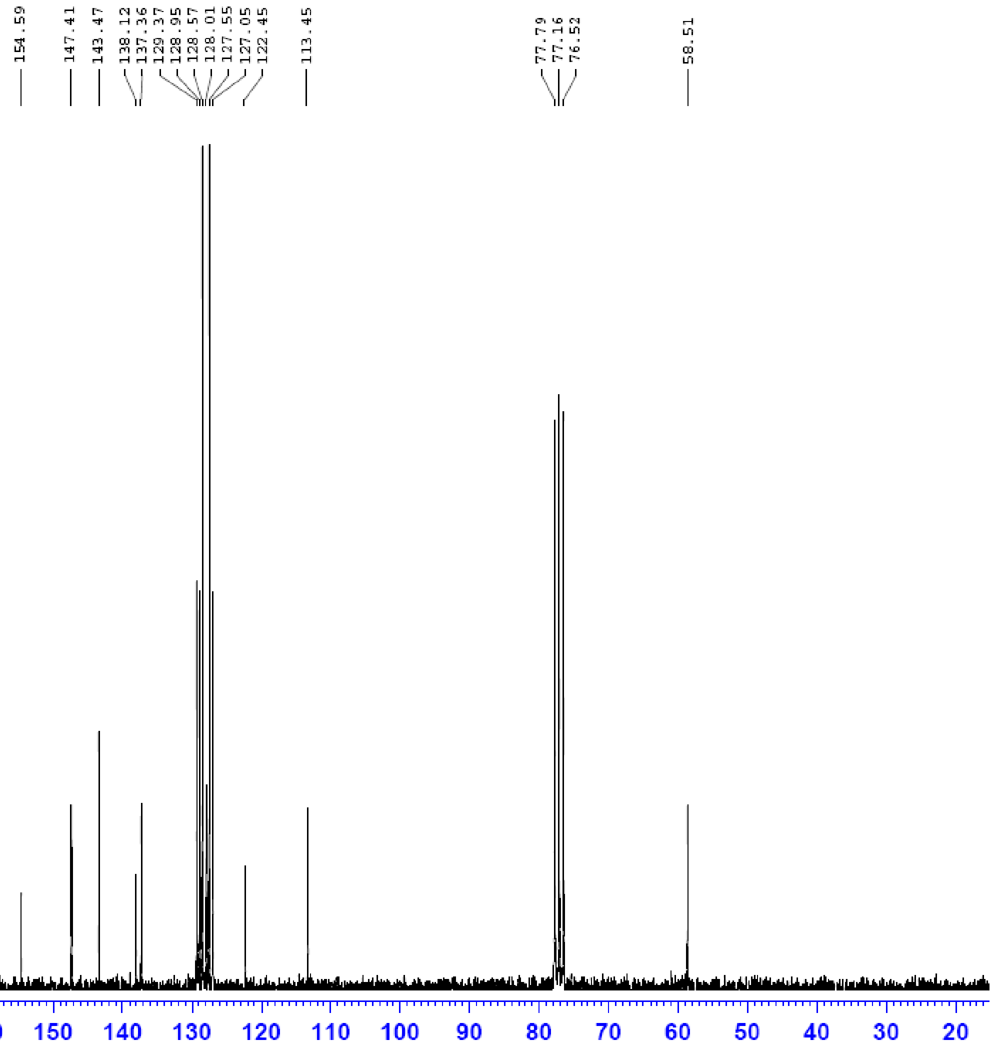
154.59
147.41
143.47
138.12
137.36
129.37
128.95
128.57
128.01
127.55
127.05
122.45
113.45
77.79
77.16
76.52
58.51
210 200 190 180 170 160 150 140 130 120 110 100 90 80 70 60 50 40 30 20

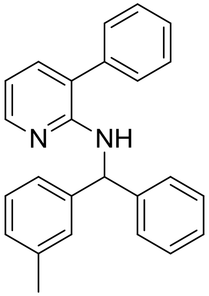

3u

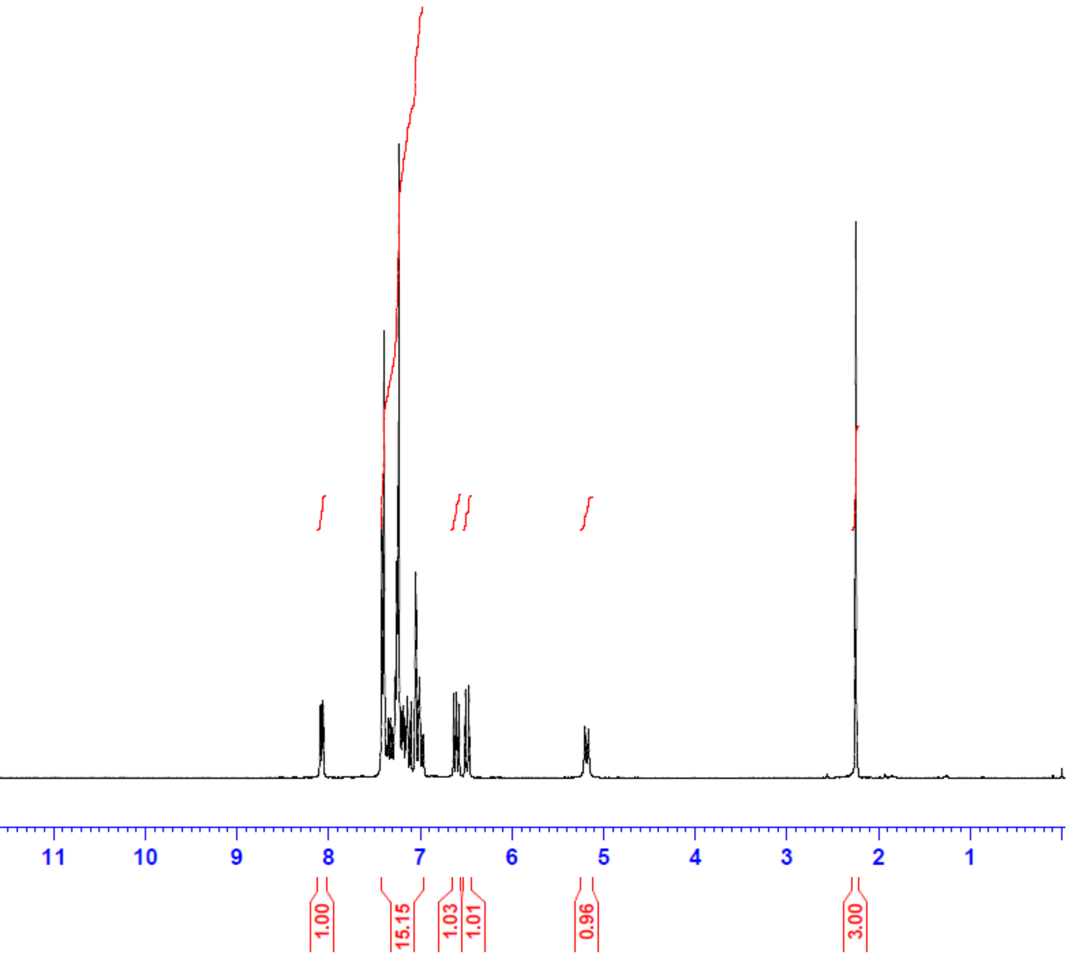

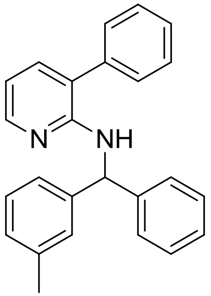
N
NH

3u

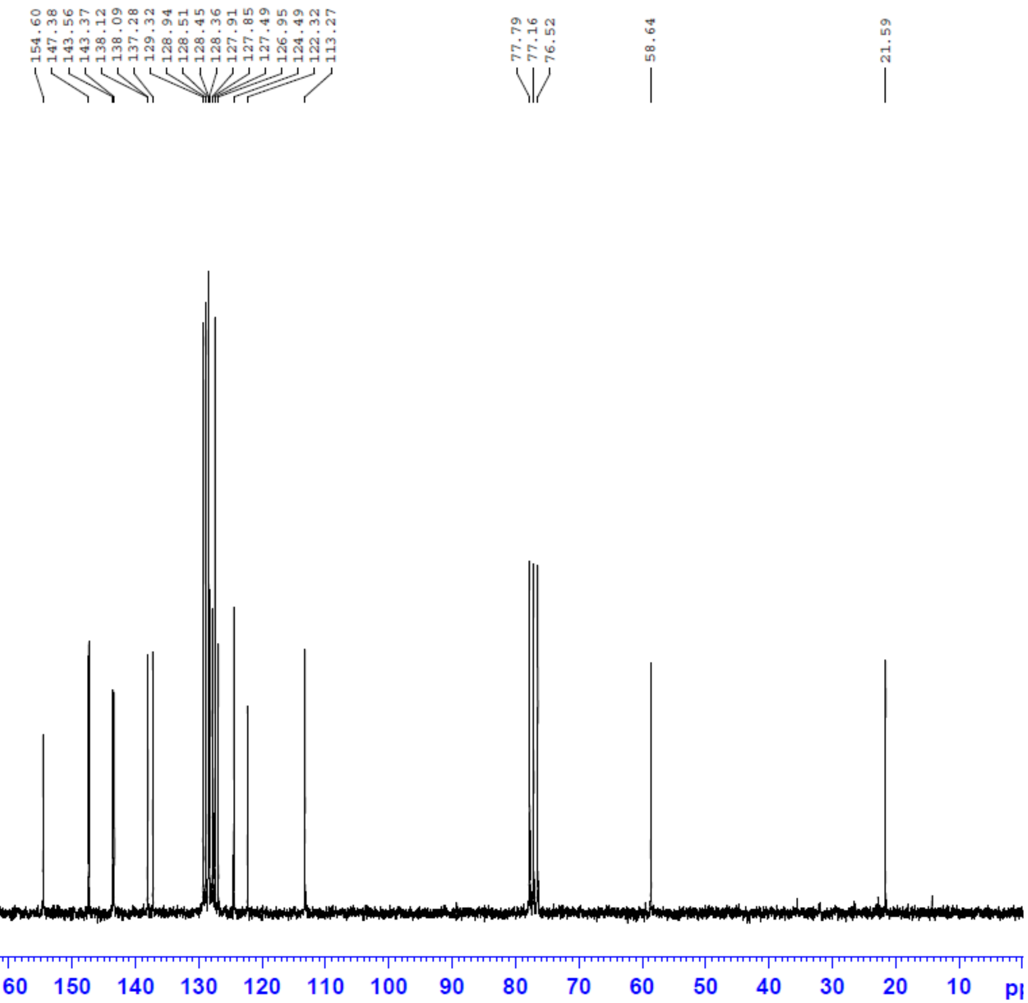
154.60
147.38
143.56
143.37
138.12
138.09
137.28
129.32
128.94
128.51
128.45
128.36
127.91
127.85
127.49
126.95
124.49
122.32
113.27
77.79
77.16
76.52
58.64
21.59
220 210 200 190 180 170 160 150 140 130 120 110 100 90 80 70 60 50 40 30 20 10

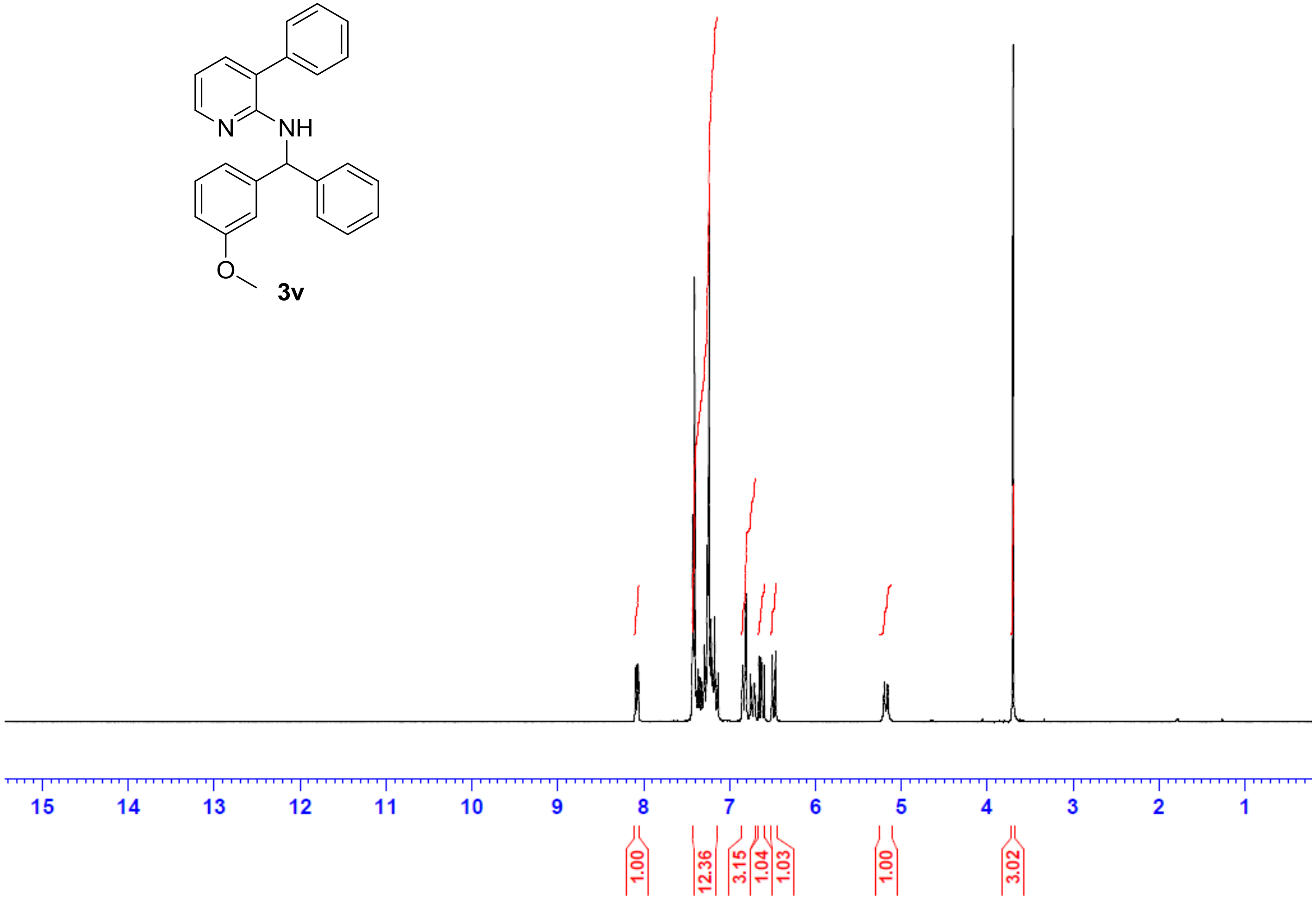

3v
15
14
13
12
11
10
9
8
7
6
5
4
3
2
1
1.00
12.36
3.15
1.04
1.03
1.00
3.02

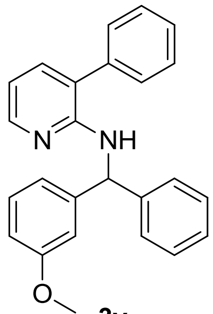

3v

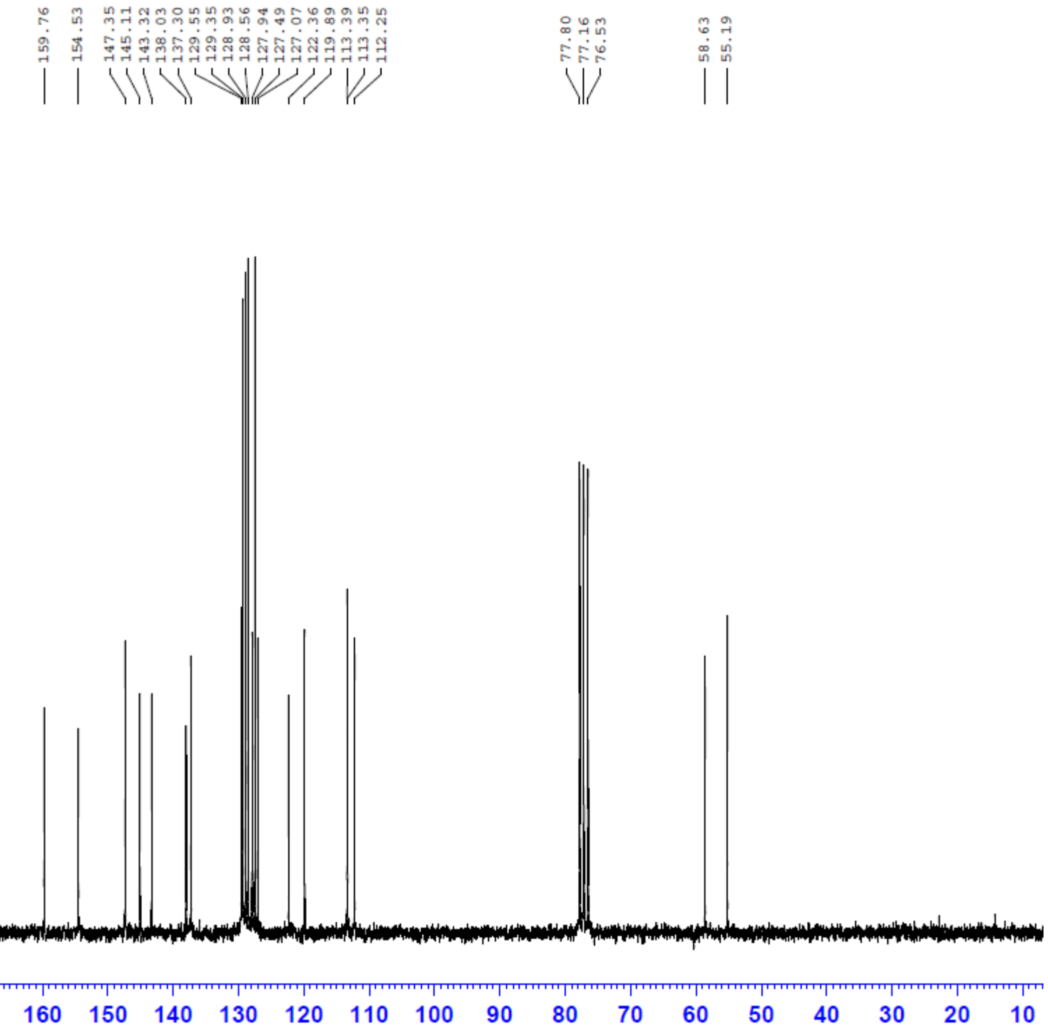
159.76
154.53
147.35
145.11
143.32
138.03
137.30
129.55
129.35
128.93
128.56
127.94
127.49
127.07
122.36
119.89
113.39
113.35
112.25
77.80
77.16
76.53
58.63
55.19
220
210
200
190
180
170
160
150
140
130
120
110
100
90
80
70
60
50
40
30
20
10

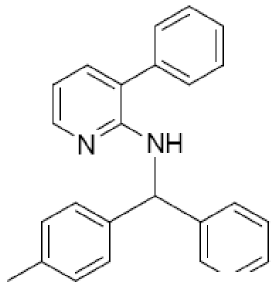

3w

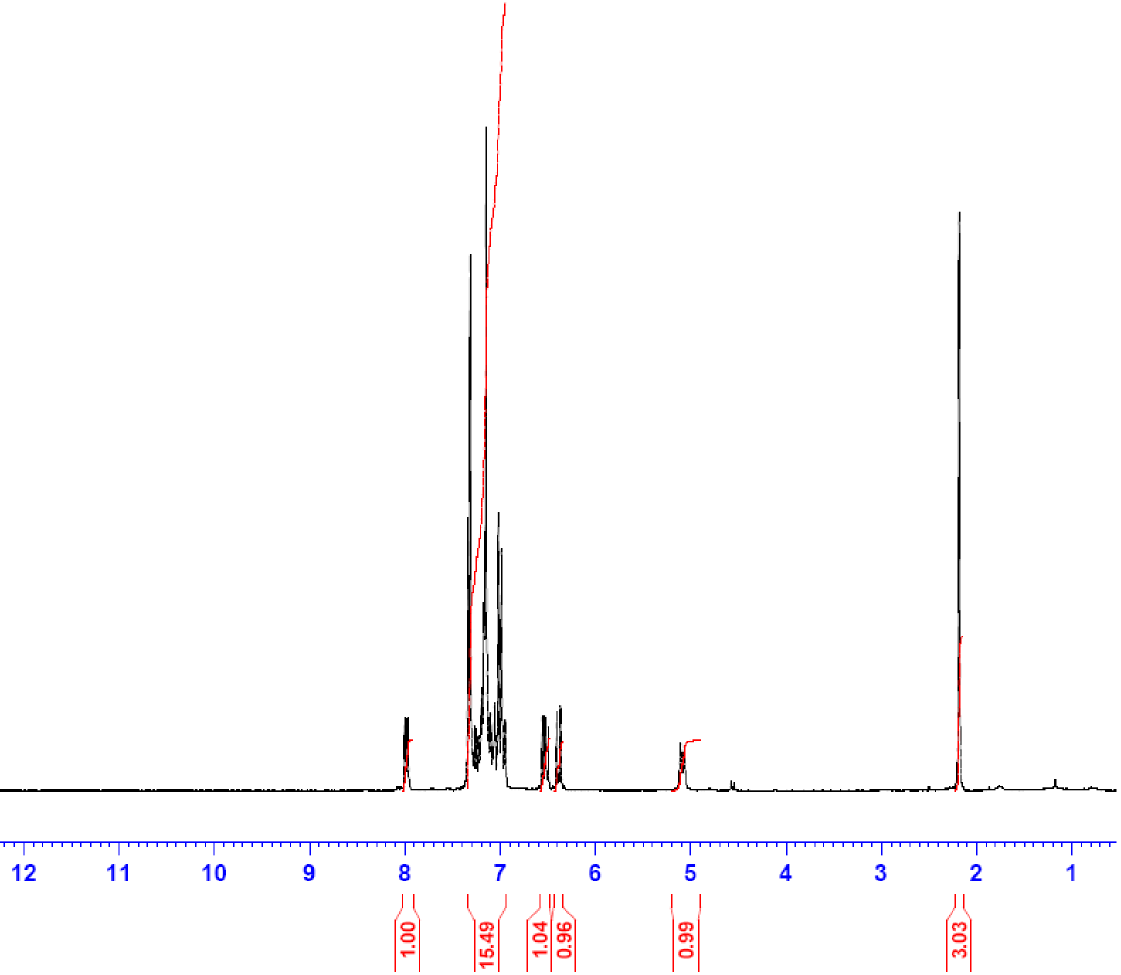

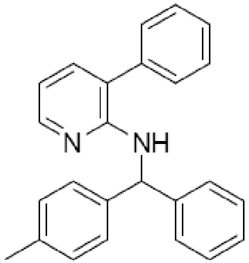
N
NH

3w

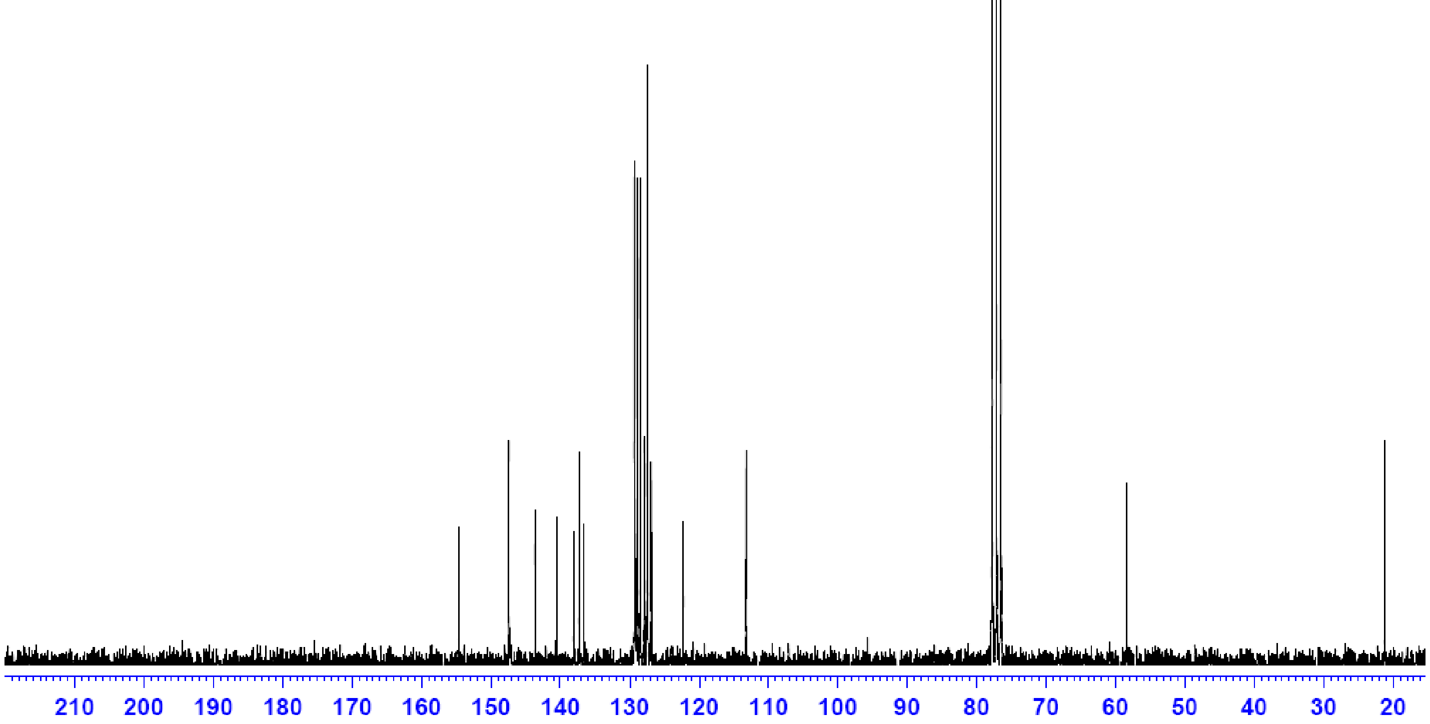
154.62
147.40
143.65
140.54
138.12
137.30
136.65
129.36
129.29
128.97
128.53
127.93
127.49
127.47
126.95
122.33
113.26
77.79
77.16
76.52
58.38
21.19
210 200 190 180 170 160 150 140 130 120 110 100 90 80 70 60 50 40 30 20

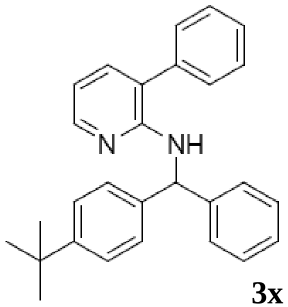

3x

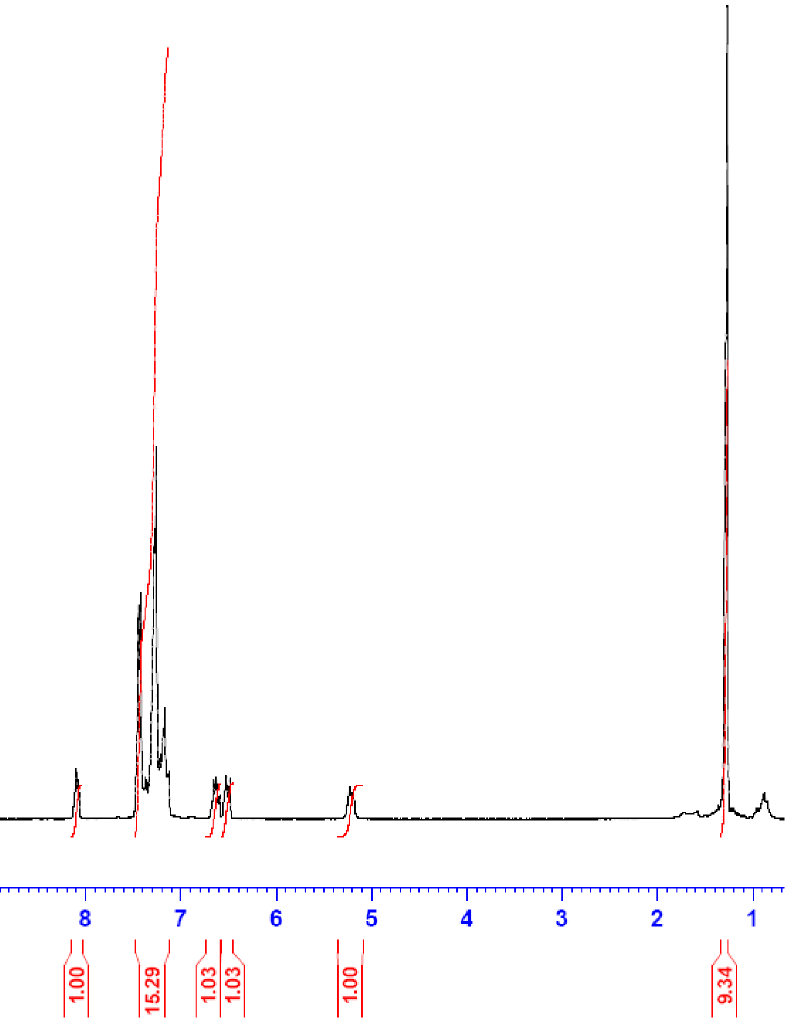

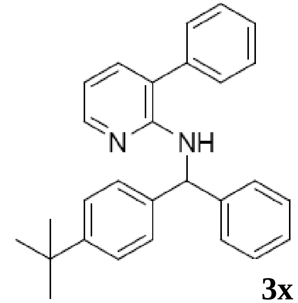
N
NH

3x

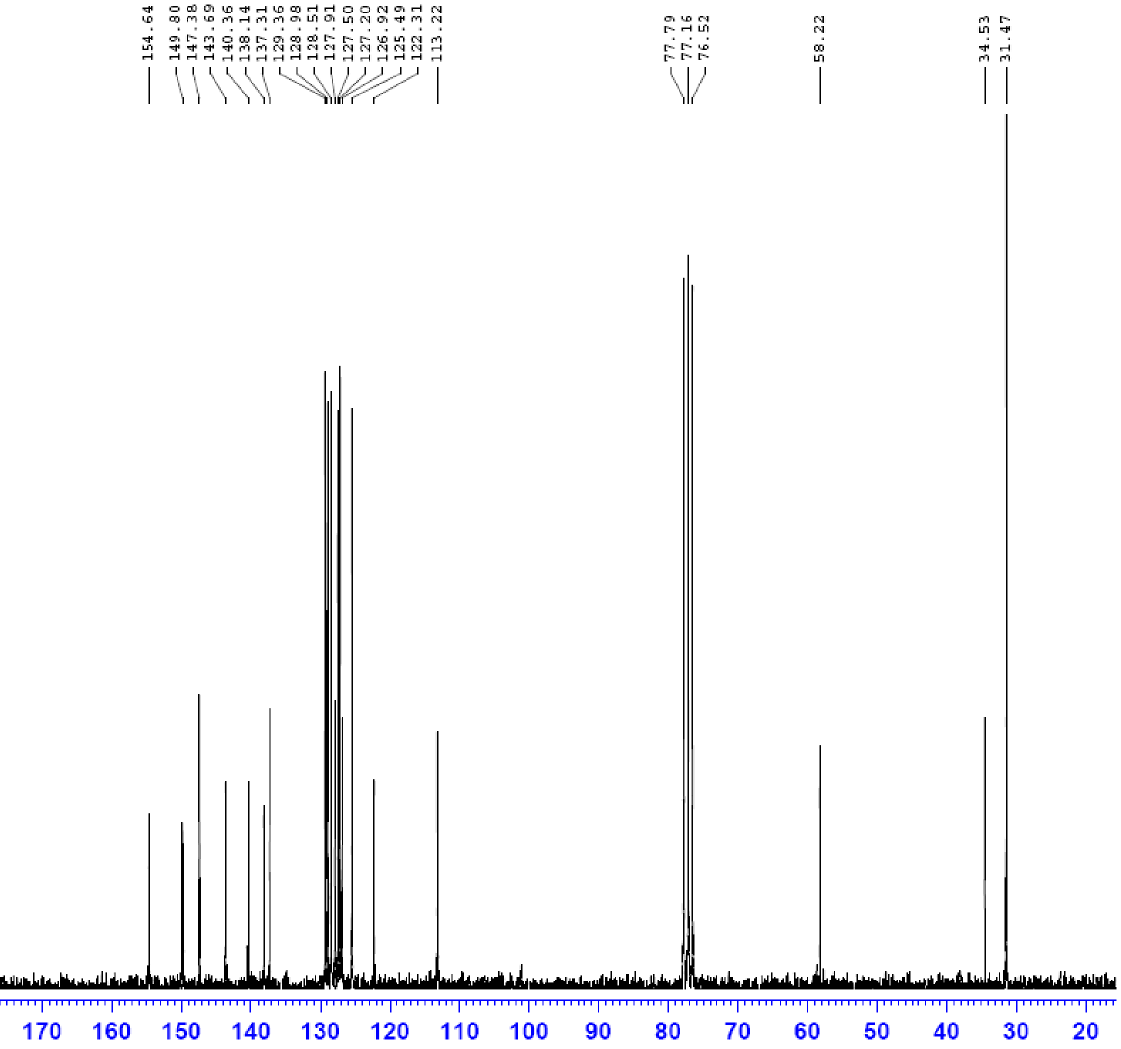
154.64
149.80
147.38
143.69
140.36
138.14
137.31
129.36
128.98
128.51
127.91
127.50
127.20
126.92
125.49
122.31
113.22
77.79
77.16
76.52
58.22
34.53
31.47
210 200 190 180 170 160 150 140 130 120 110 100 90 80 70 60 50 40 30 20

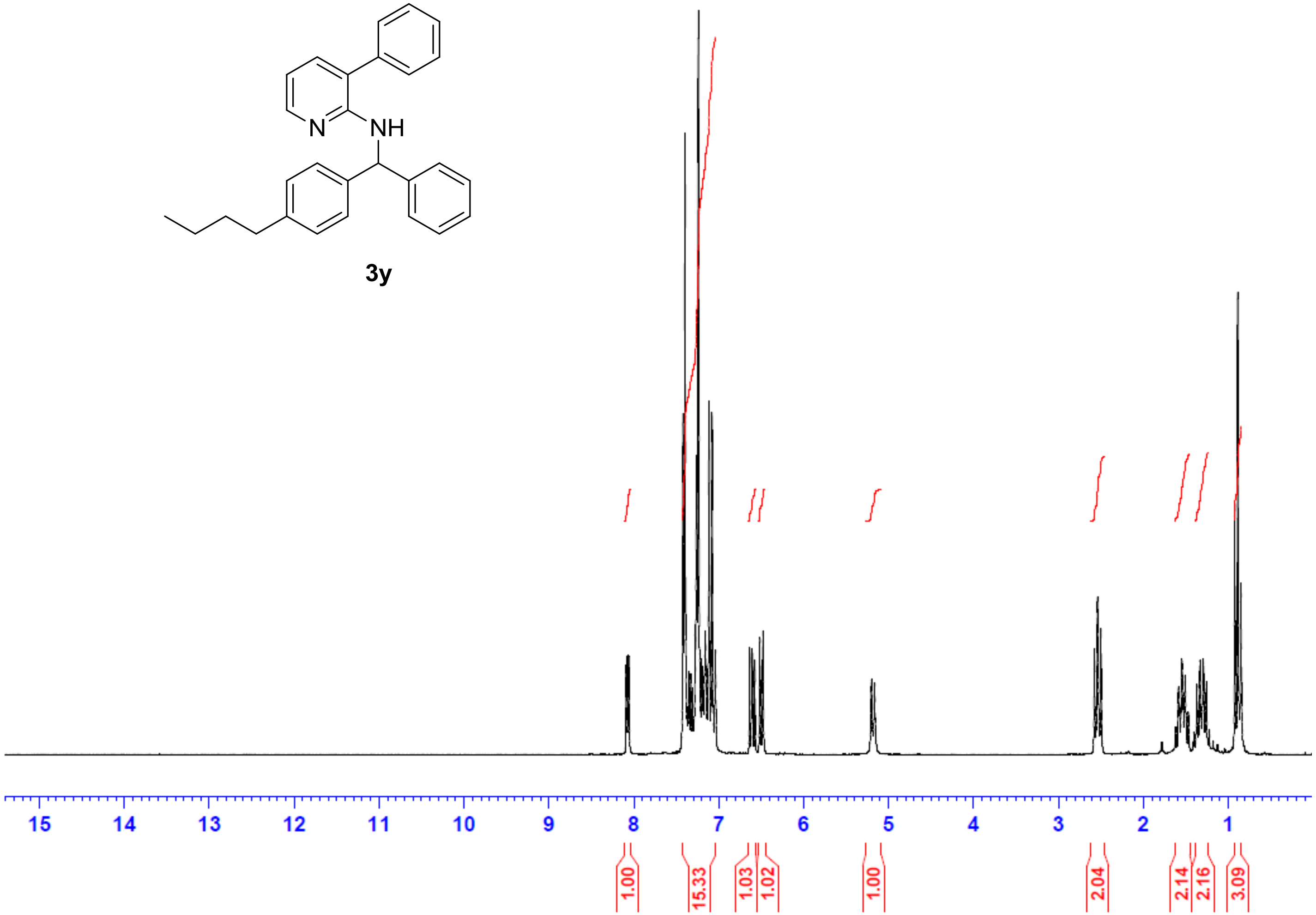
N
NH
3y
15 14 13 12 11 10 9 8 7 6 5 4 3 2 1
1.00
15.33
1.03
1.02
1.00
2.04
2.14
2.16
3.09

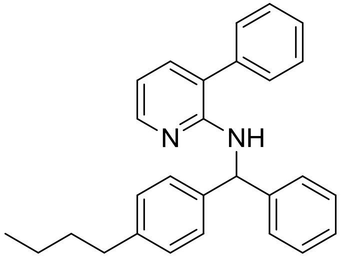

3y

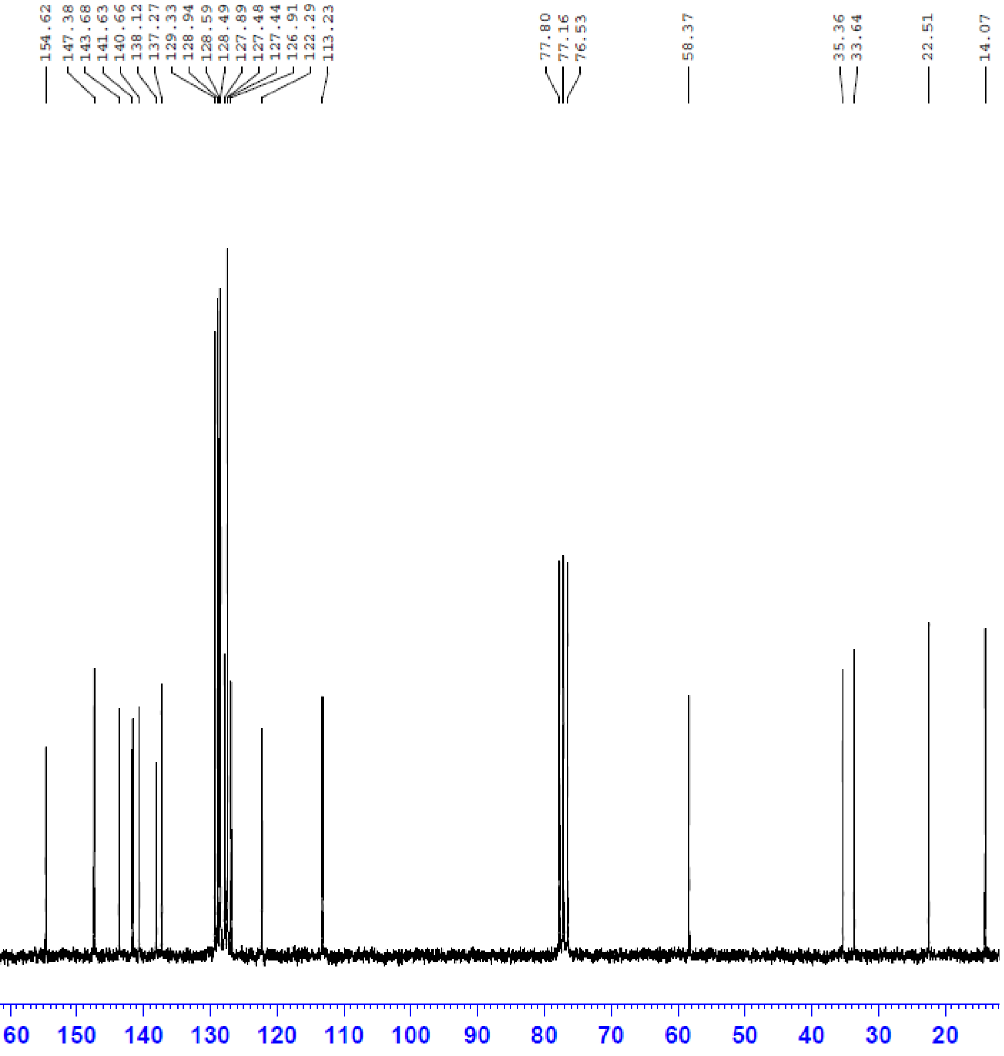
154.62
147.38
143.68
141.63
140.66
138.12
137.27
129.33
128.94
128.59
128.49
127.89
127.48
127.44
126.91
122.29
113.23
77.80
77.16
76.53
58.37
35.36
33.64
22.51
14.07
220 210 200 190 180 170 160 150 140 130 120 110 100 90 80 70 60 50 40 30 20

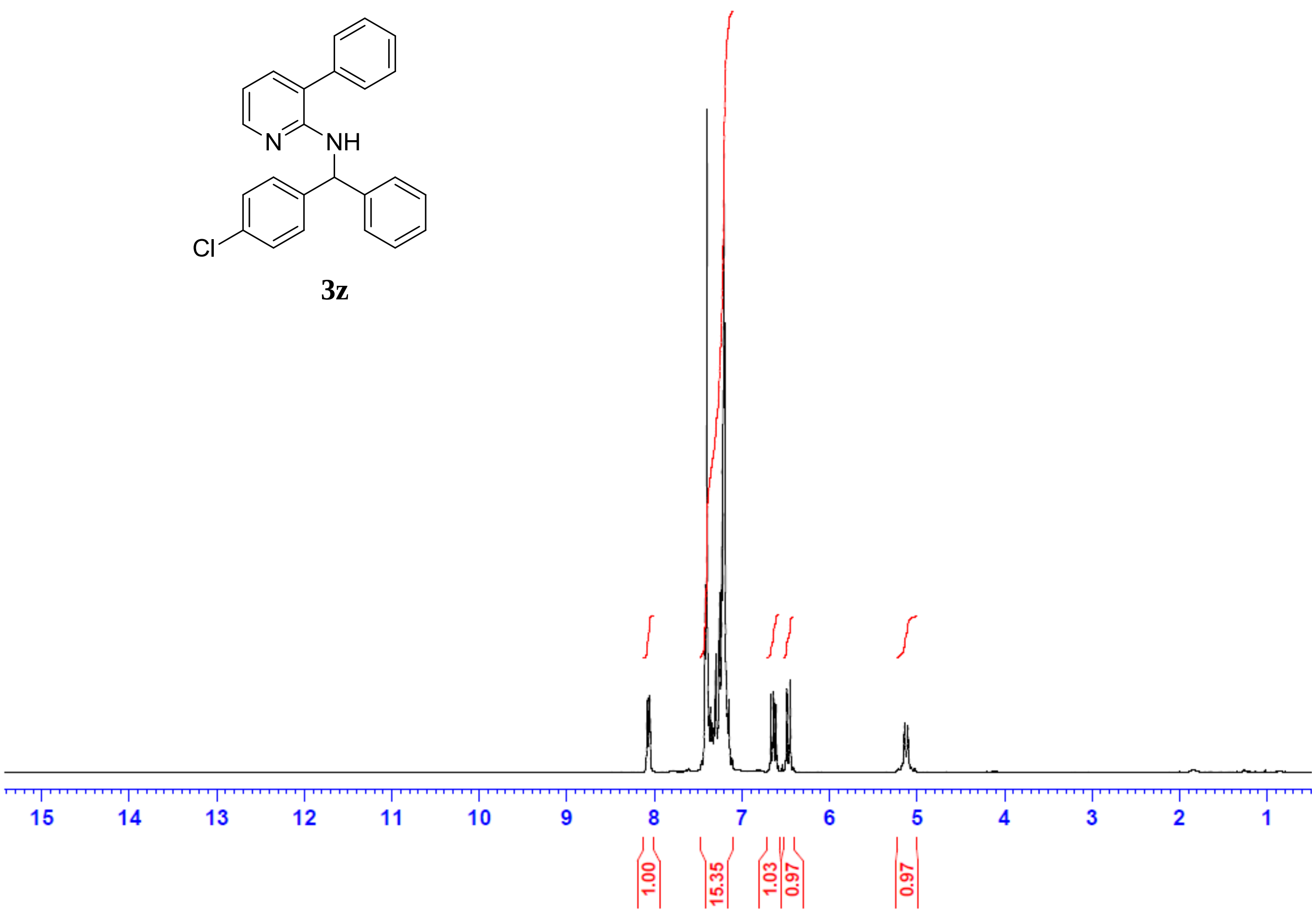

3z

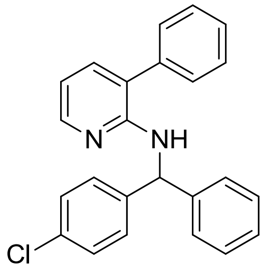

3z

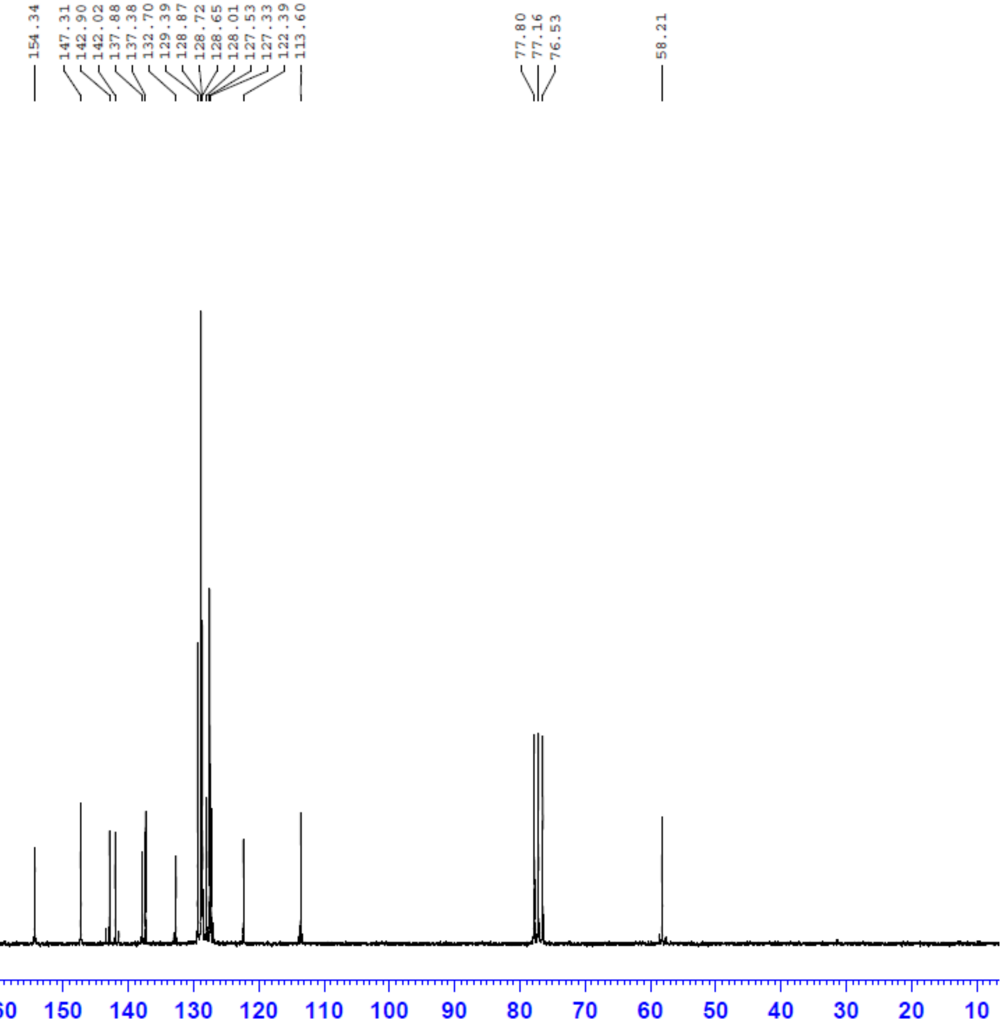
154.34
147.31
142.90
142.02
137.88
137.38
132.70
129.39
128.87
128.72
128.65
128.01
127.53
127.33
122.39
113.60
77.80
77.16
76.53
58.21
220 210 200 190 180 170 160 150 140 130 120 110 100 90 80 70 60 50 40 30 20 10

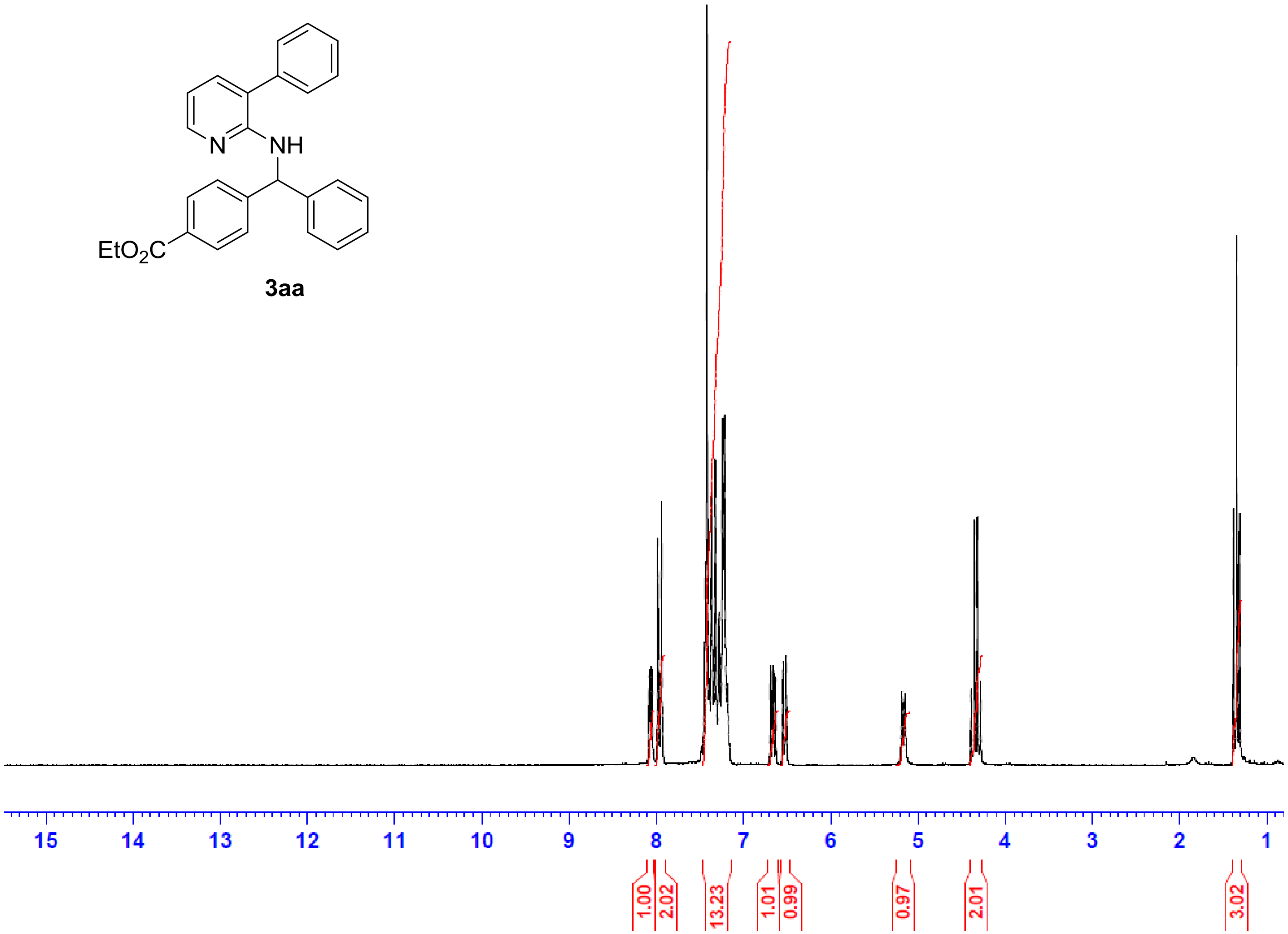
N
NH
EtO2C
3aa
15
14
13
12
11
10
9
8
7
6
5
4
3
2
1
1.00
2.02
13.23
1.01
0.99
0.97
2.01
3.02

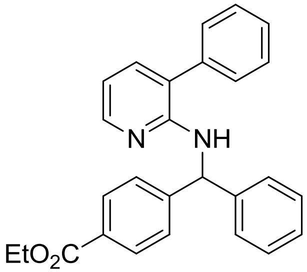

3aa

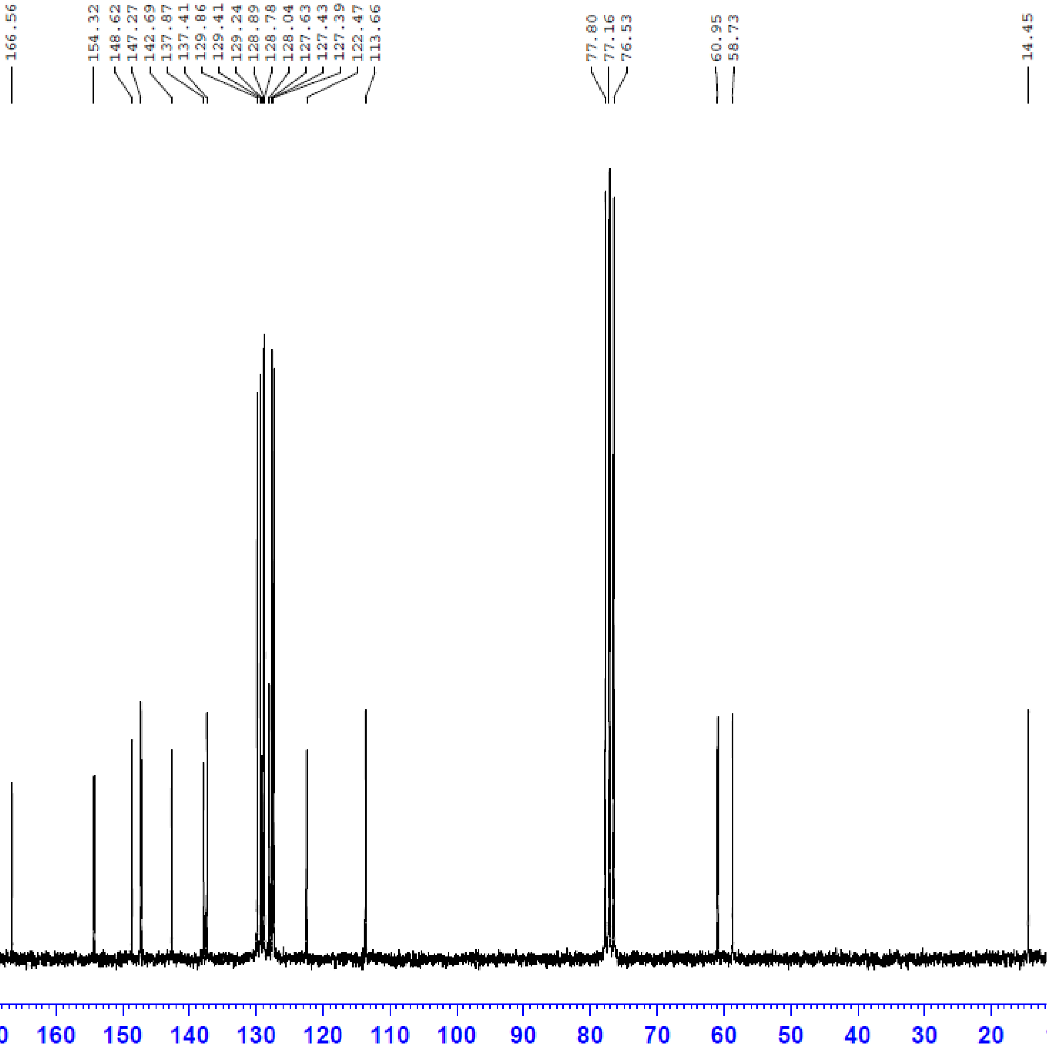
166.56
154.32
148.62
147.27
142.69
137.87
137.41
129.86
129.41
129.24
128.89
128.78
128.04
127.63
127.43
127.39
122.47
113.66
77.80
77.16
76.53
60.95
58.73
14.45
220 210 200 190 180 170 160 150 140 130 120 110 100 90 80 70 60 50 40 30 20

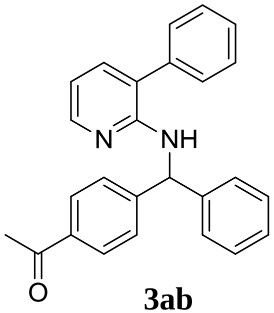

3ab

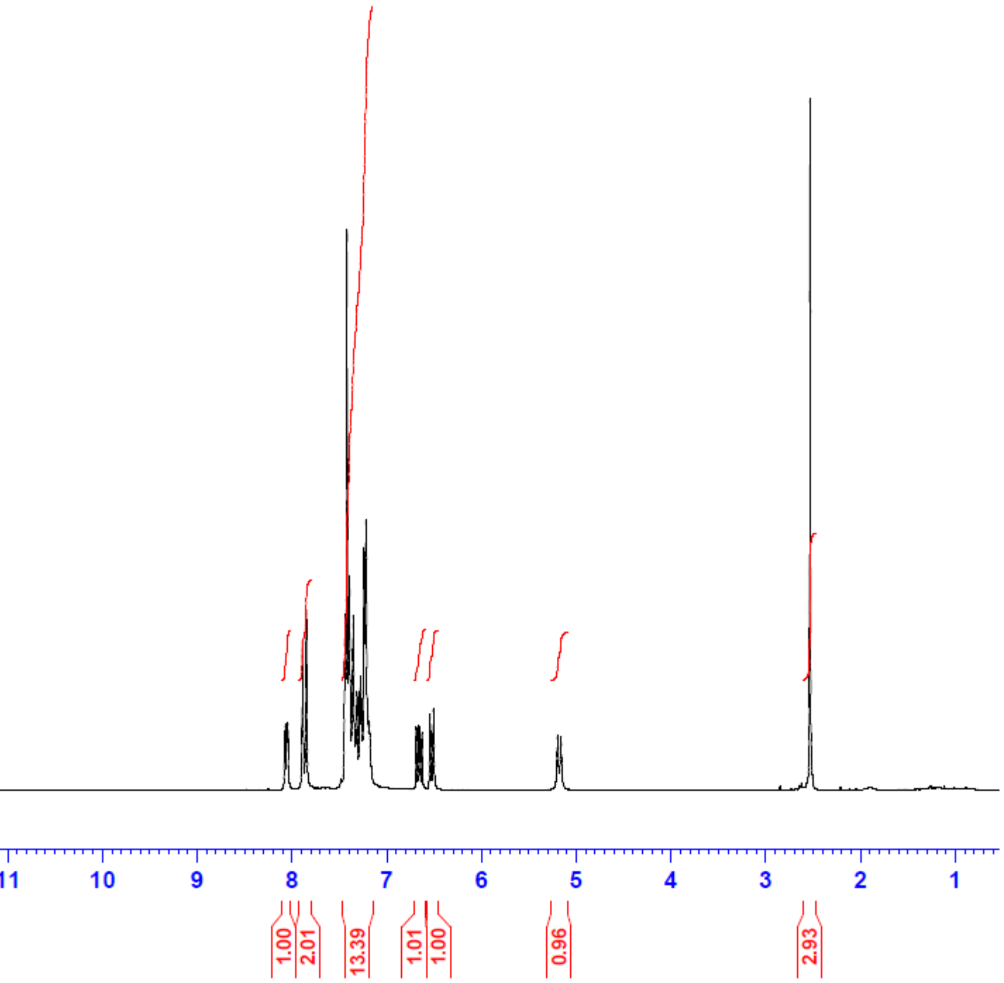

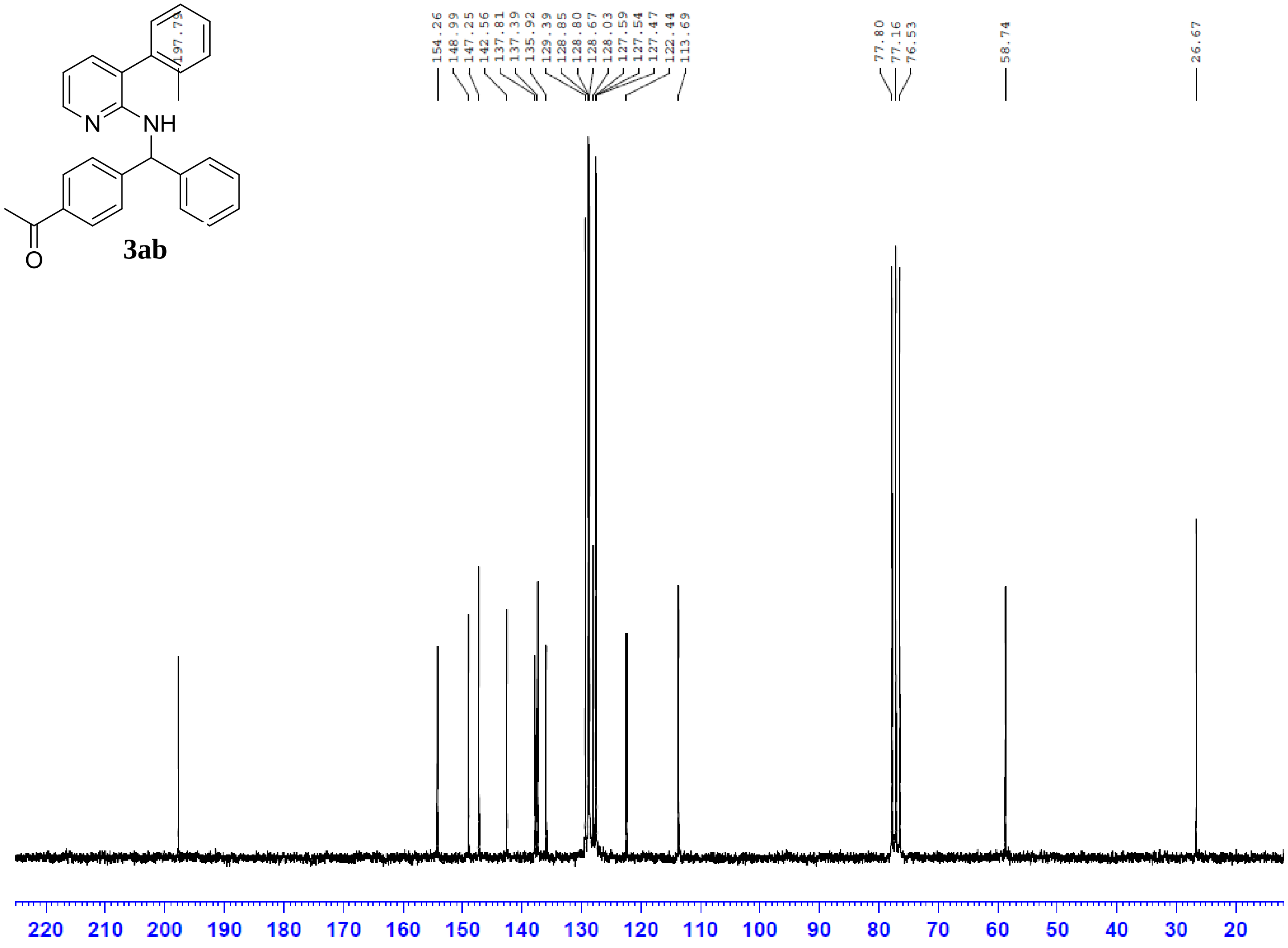

N
NH
O
197.79
154.26
148.99
147.25
142.56
137.81
137.39
135.92
129.39
128.85
128.80
128.67
128.03
127.59
127.54
127.47
122.44
113.69
77.80
77.16
76.53
58.74
26.67
220
210
200
190
180
170
160
150
140
130
120
110
100
90
80
70
60
50
40
30
20

3ab

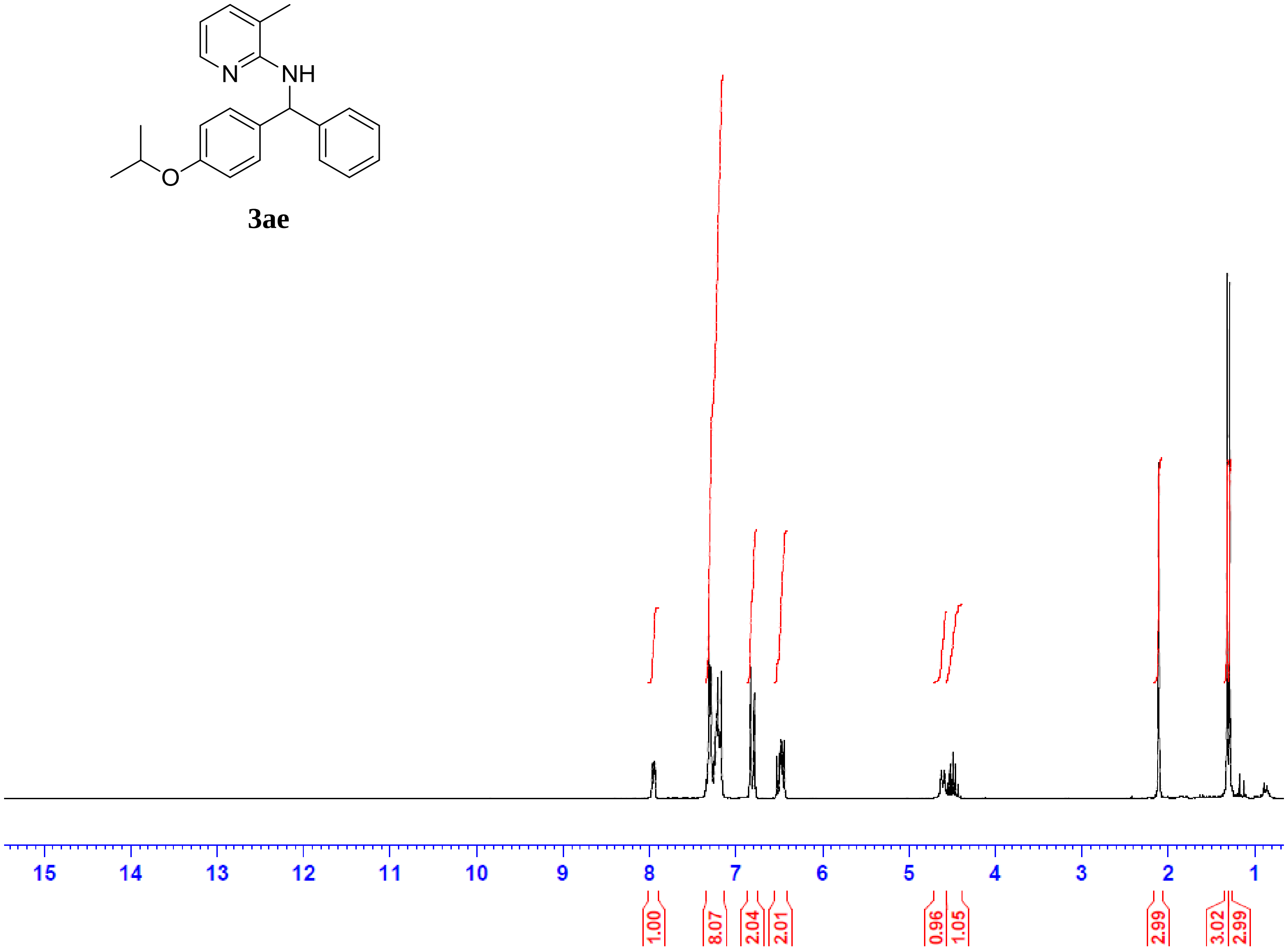
N
NH
O
15 14 13 12 11 10 9 8 7 6 5 4 3 2 1
1.00
8.07
2.04
2.01
0.96
1.05
2.99
3.02
2.99

3ae

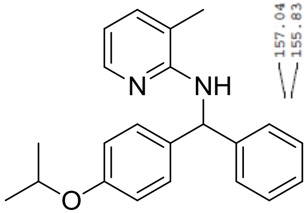

N
NH
O

3ae

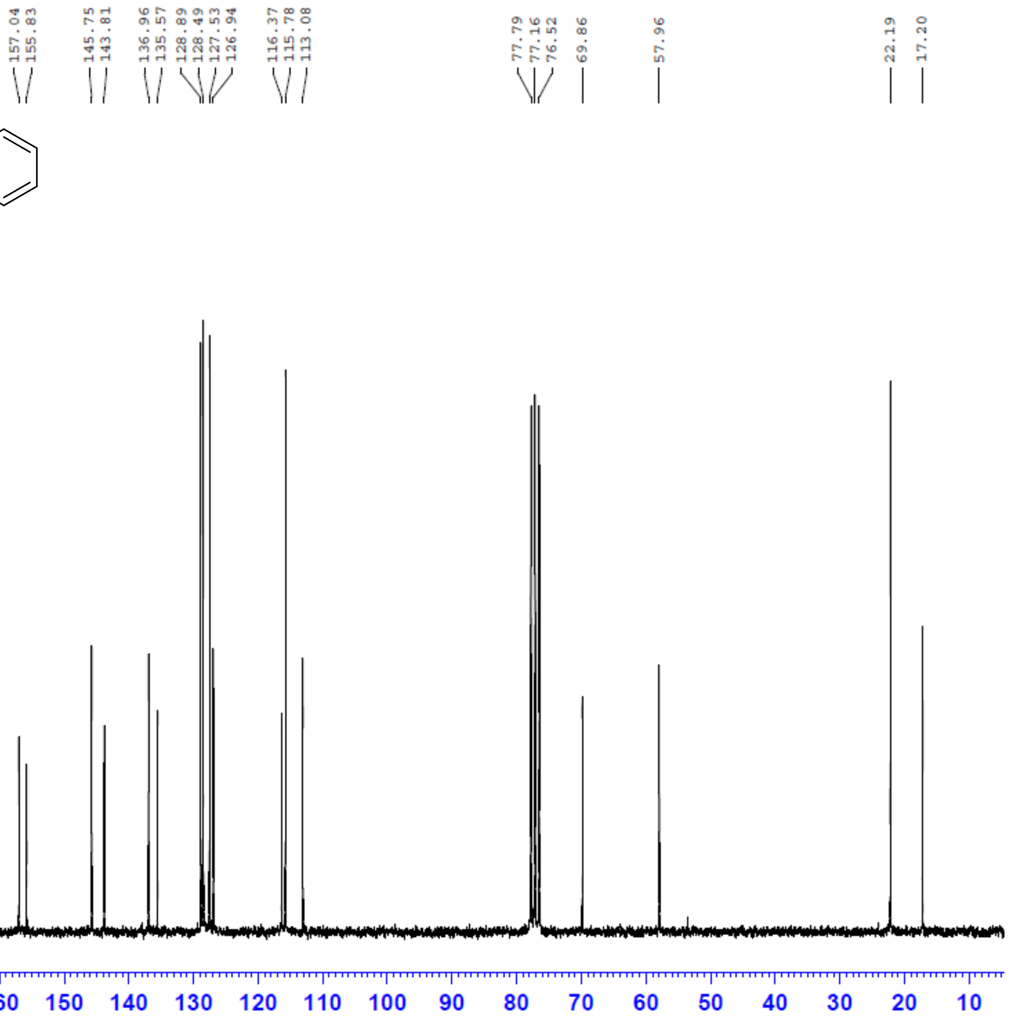

157.04
155.83
145.75
143.81
136.96
135.57
128.89
128.49
127.53
126.94
116.37
115.78
113.08
77.79
77.16
76.52
69.86
57.96
22.19
17.20
220 210 200 190 180 170 160 150 140 130 120 110 100 90 80 70 60 50 40 30 20 10

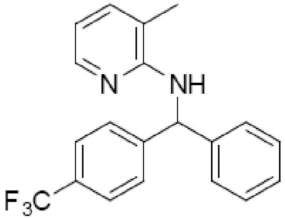

3af

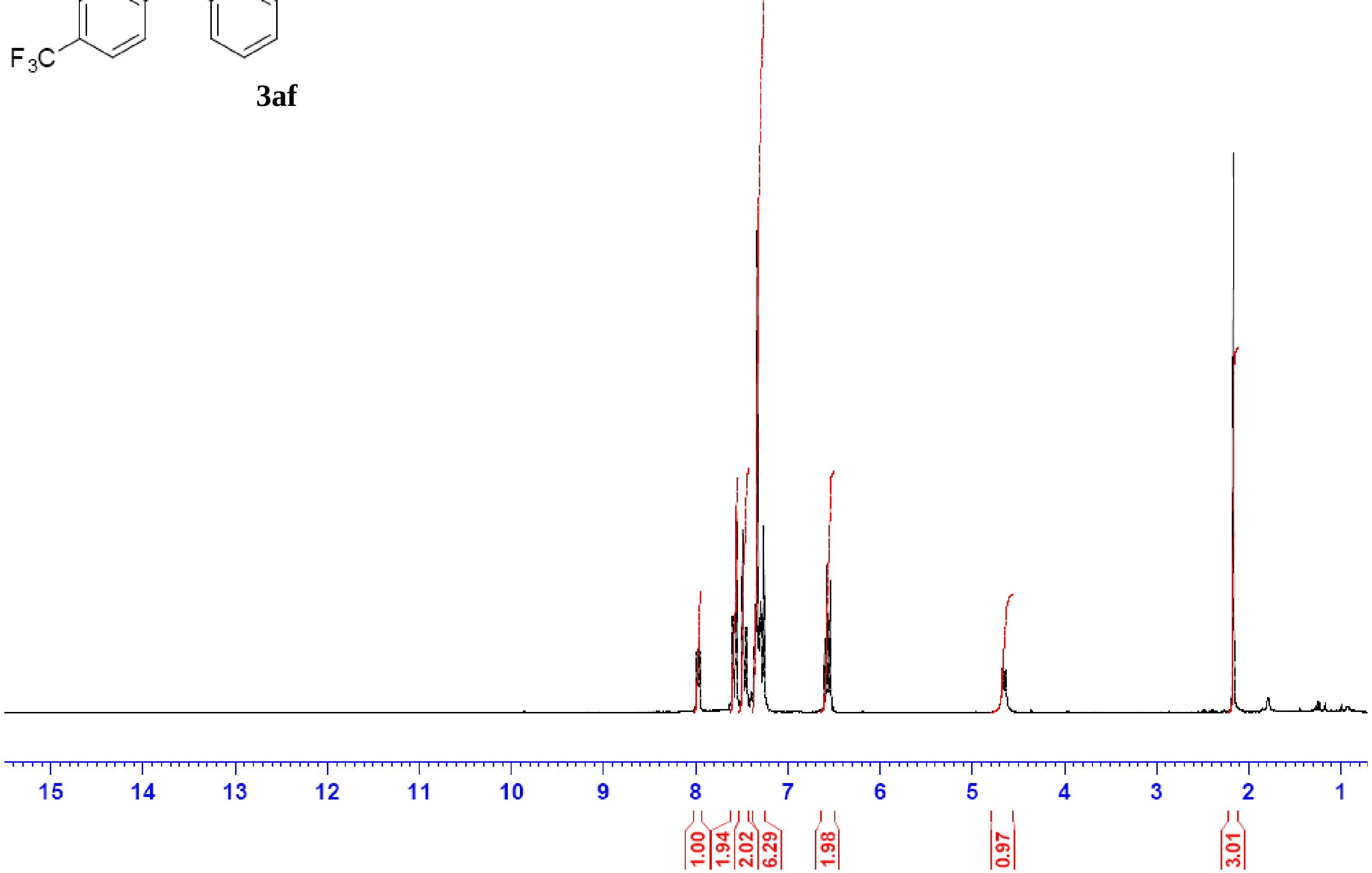

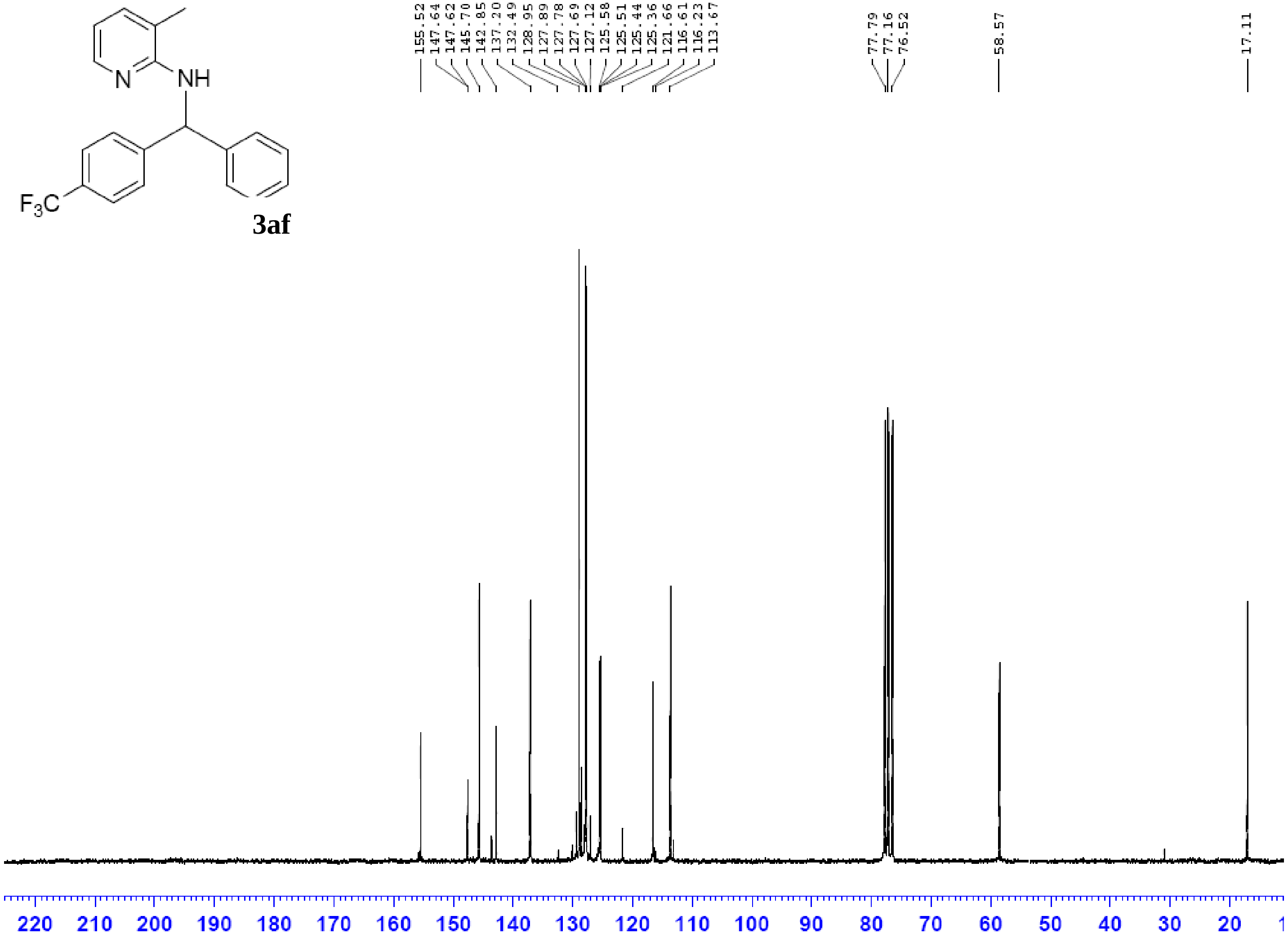

N
NH
F3C
3af
155.52
147.64
147.62
145.70
142.85
137.20
132.49
128.95
127.89
127.78
127.69
127.12
125.58
125.51
125.44
125.36
121.66
116.61
116.23
113.67
77.79
77.16
76.52
58.57
17.11
220 210 200 190 180 170 160 150 140 130 120 110 100 90 80 70 60 50 40 30 20

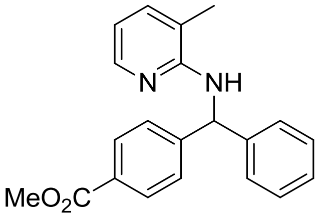
NH
N
MeO2C

3ag

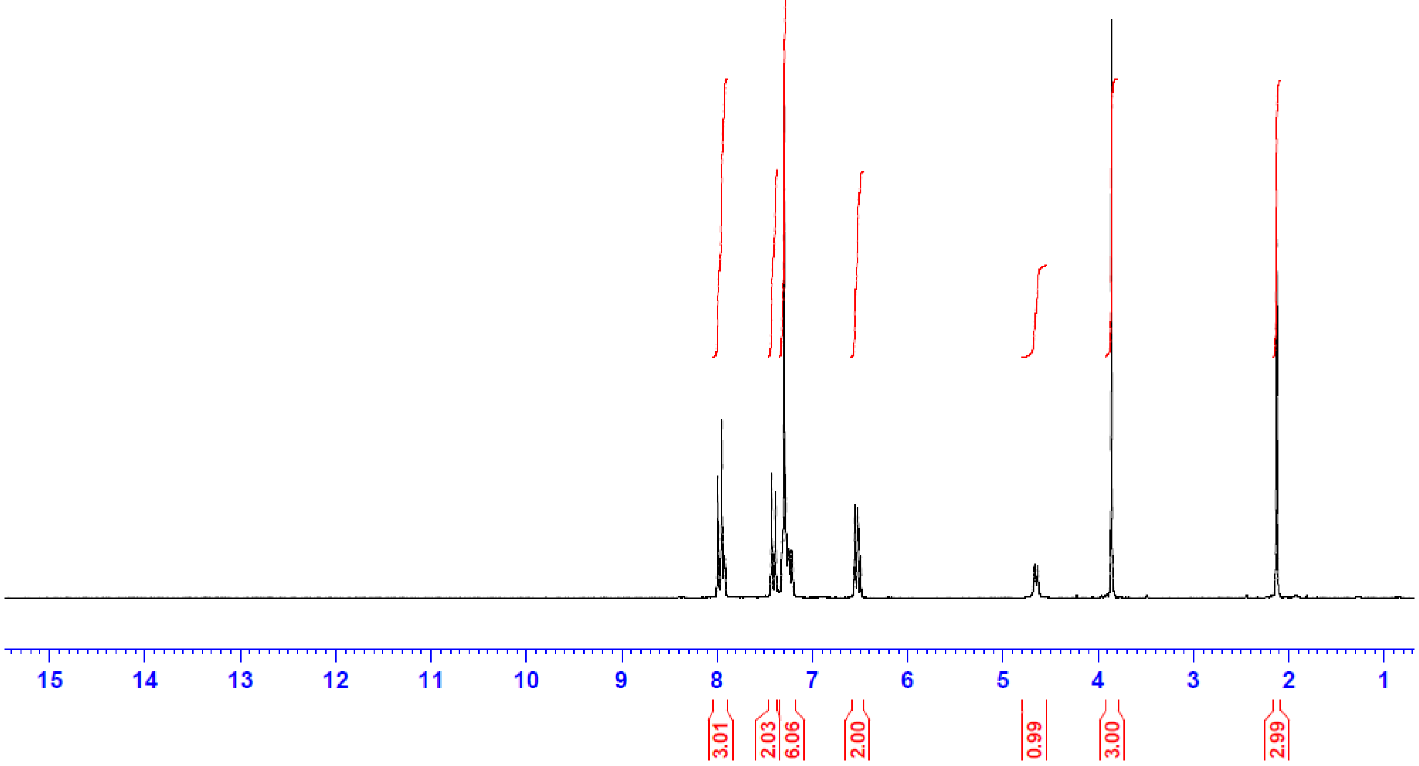
15
14
13
12
11
10
9
8
7
6
5
4
3
2
1
3.01
2.03
6.06
2.00
0.99
3.00
2.99

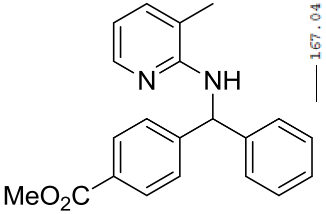
N
NH
MeO2C

3ag

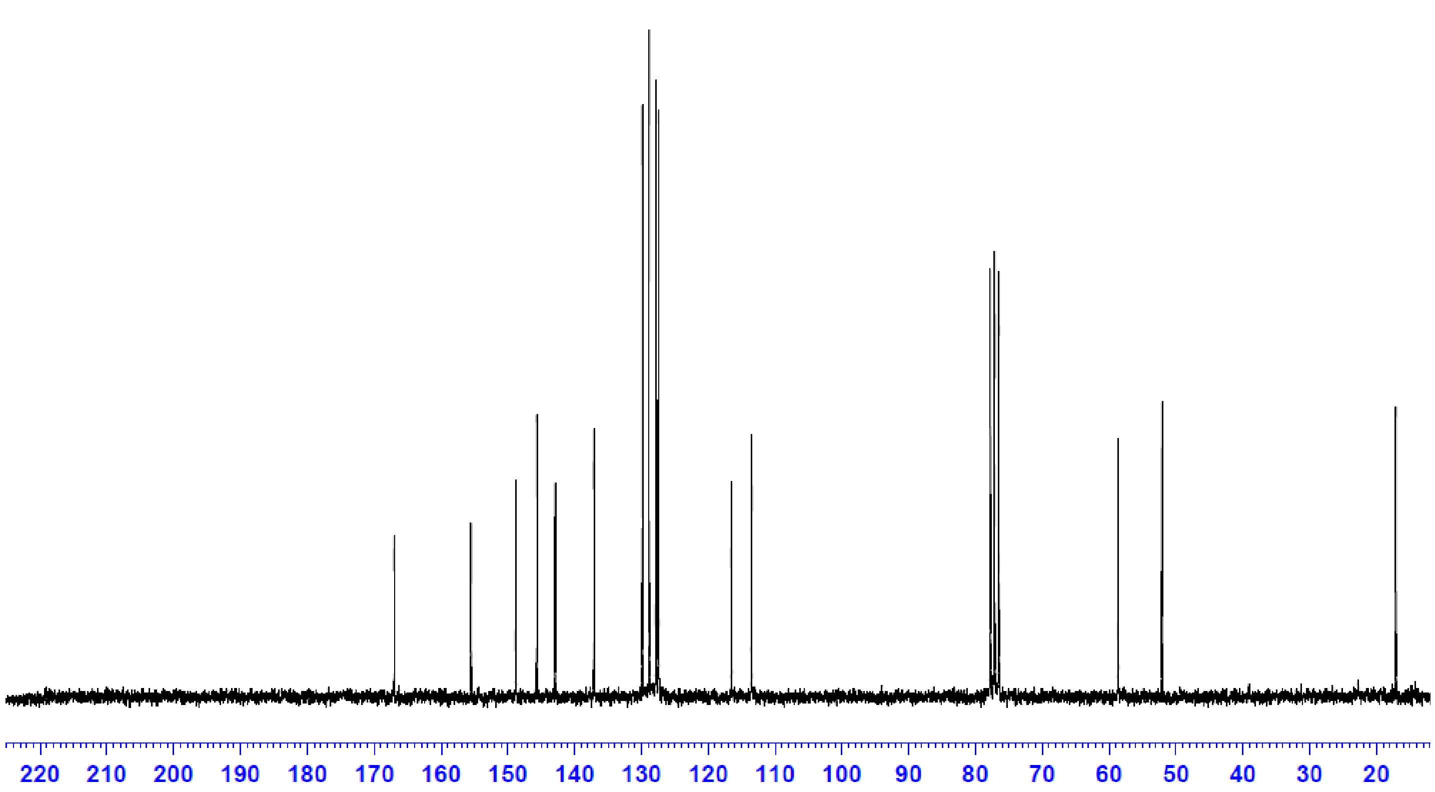
167.04
155.54
148.81
145.66
142.92
137.12
129.86
128.84
127.86
127.55
127.45
116.55
113.53
77.79
77.16
76.52
58.65
52.09
17.11
220 210 200 190 180 170 160 150 140 130 120 110 100 90 80 70 60 50 40 30 20

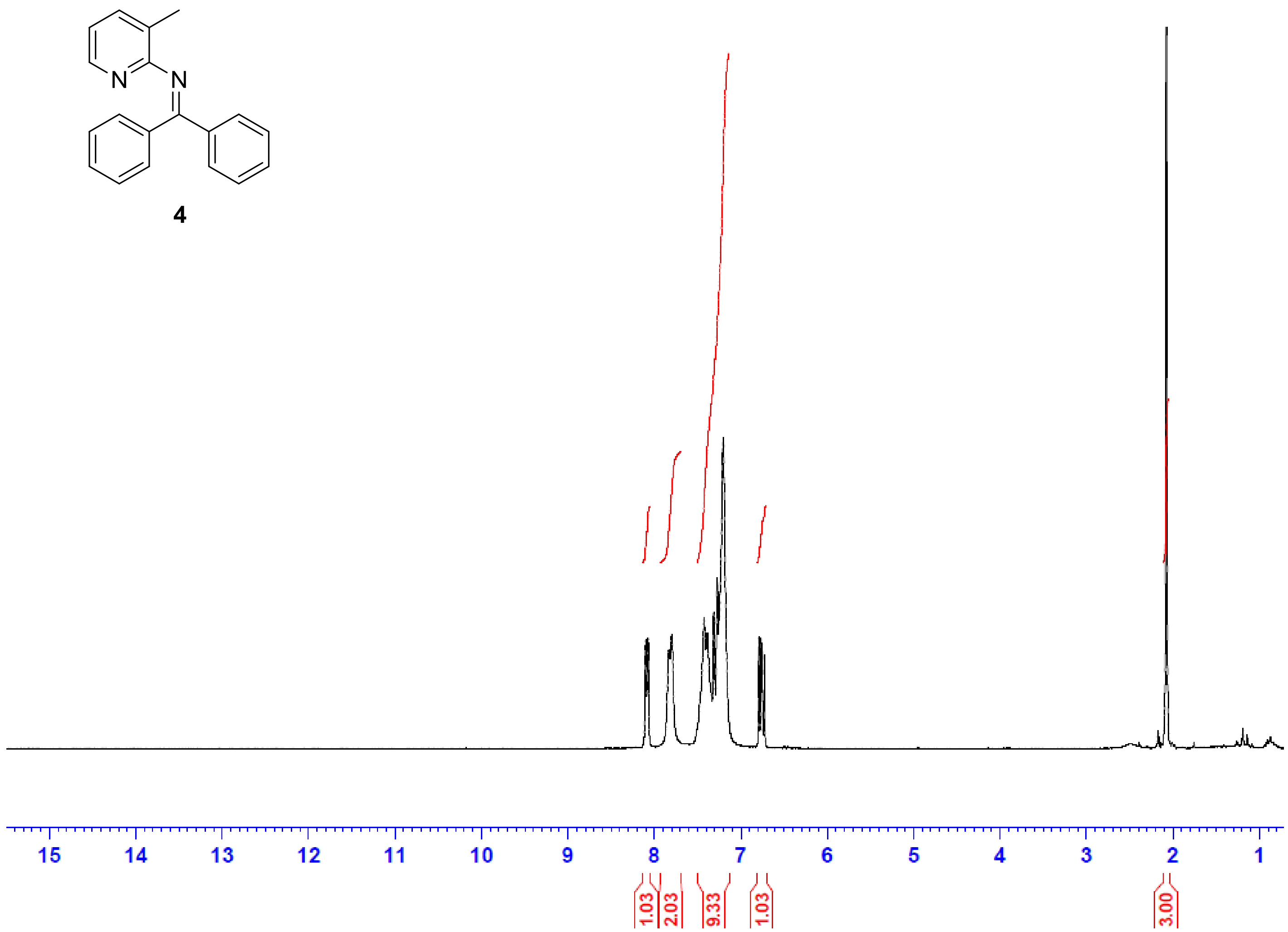

N
N
4
15
14
13
12
11
10
9
8
7
6
5
4
3
2
1
1.03
2.03
9.33
1.03
3.00

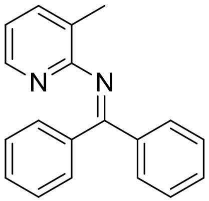
N
N

4

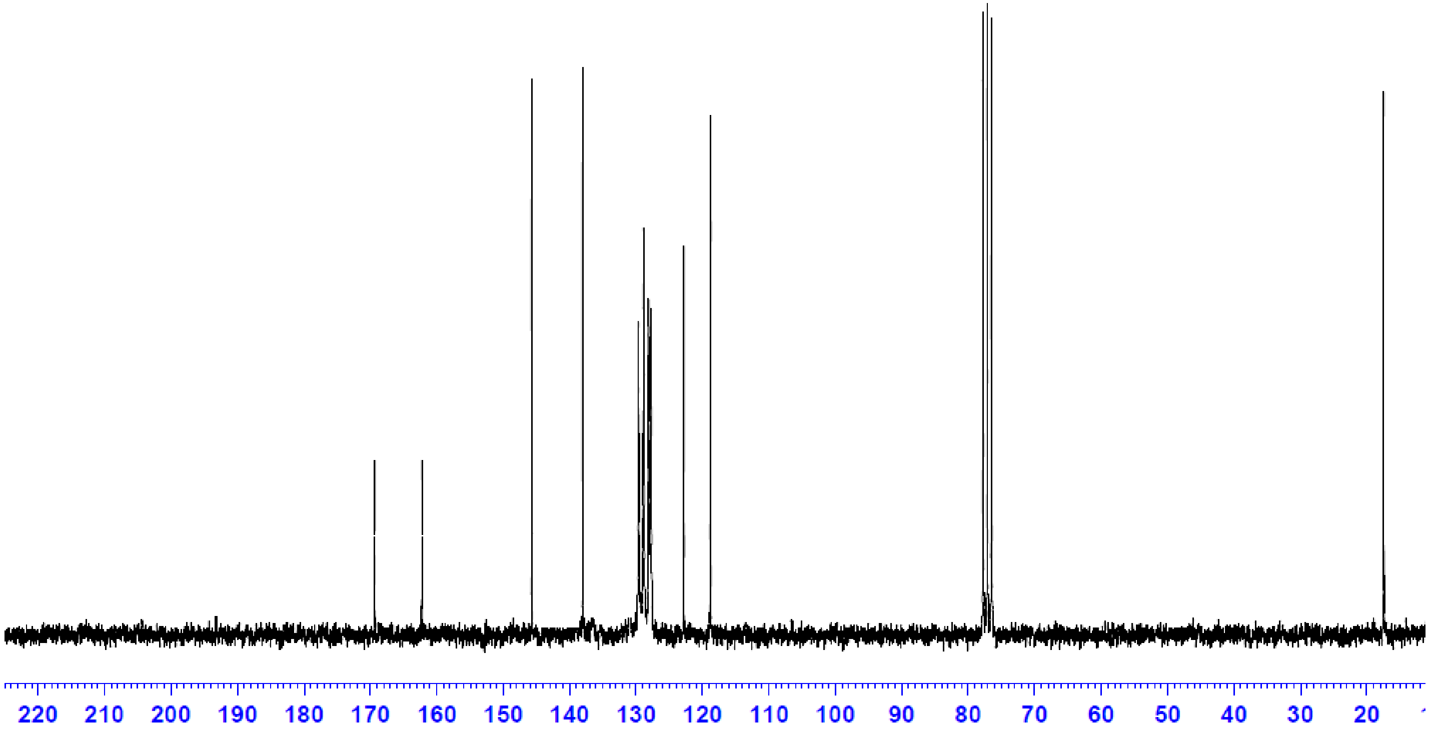
169.37
162.27
145.75
138.06
129.66
128.88
128.12
127.77
122.81
118.80
77.79
77.16
76.52
17.48
220 210 200 190 180 170 160 150 140 130 120 110 100 90 80 70 60 50 40 30 20

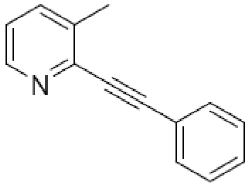

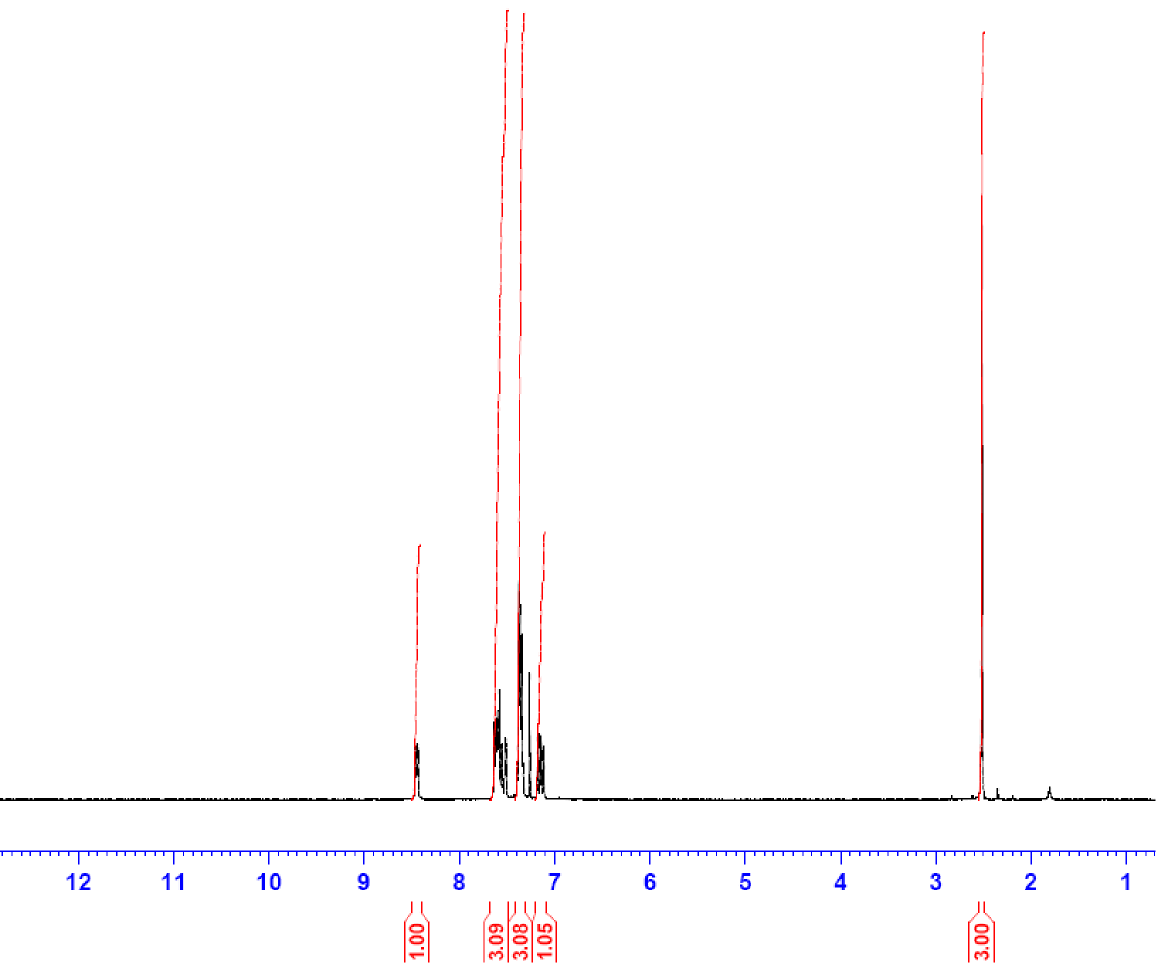

15 14 13 12 11 10 9 8 7 6 5 4 3 2 1
1.00
3.09
3.08
1.05
3.00

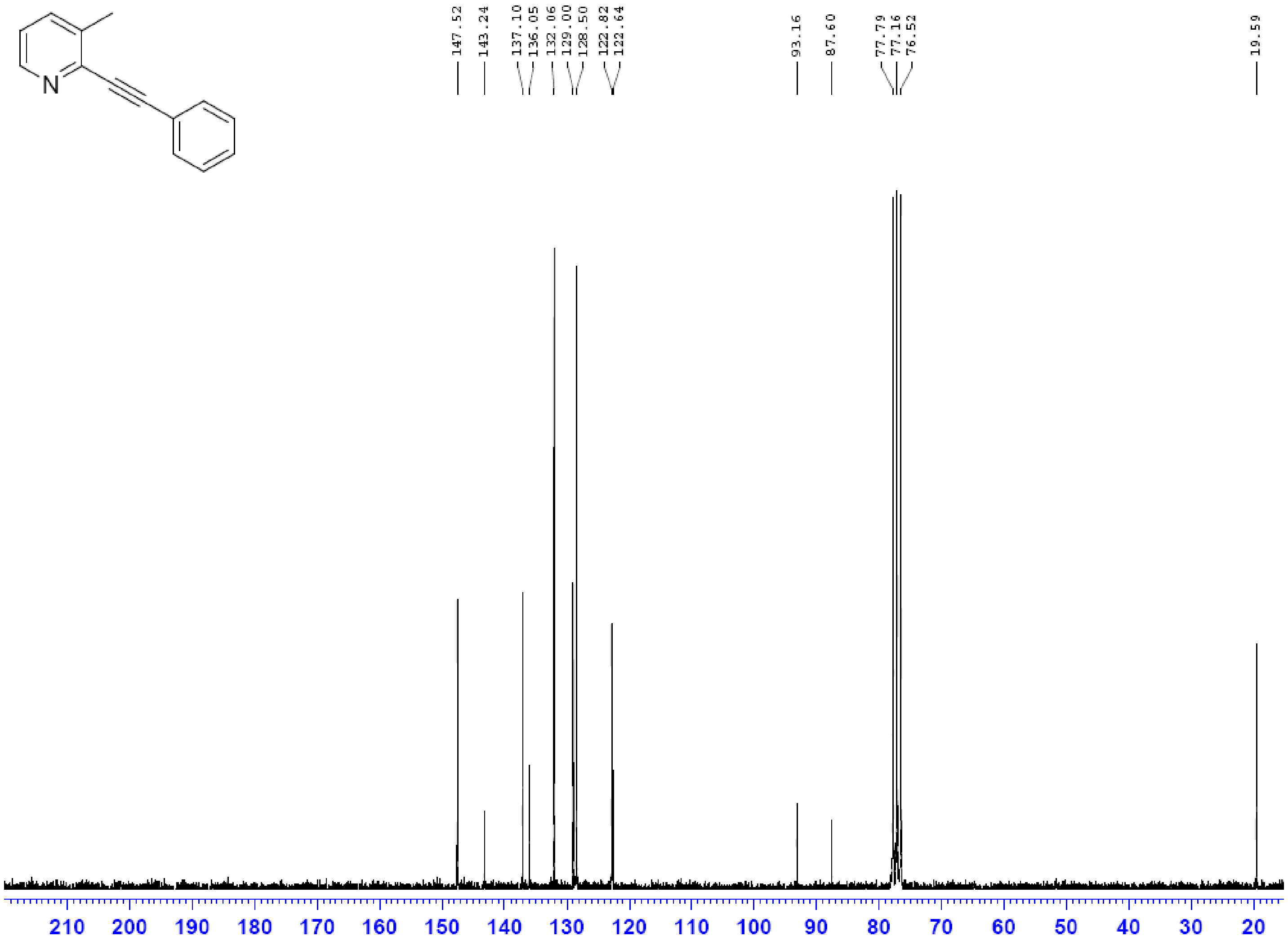

N
147.52
143.24
137.10
136.05
132.06
129.00
128.50
122.82
122.64
93.16
87.60
77.79
77.16
76.52
19.59
210
200
190
180
170
160
150
140
130
120
110
100
90
80
70
60
50
40
30
20

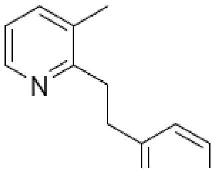

5

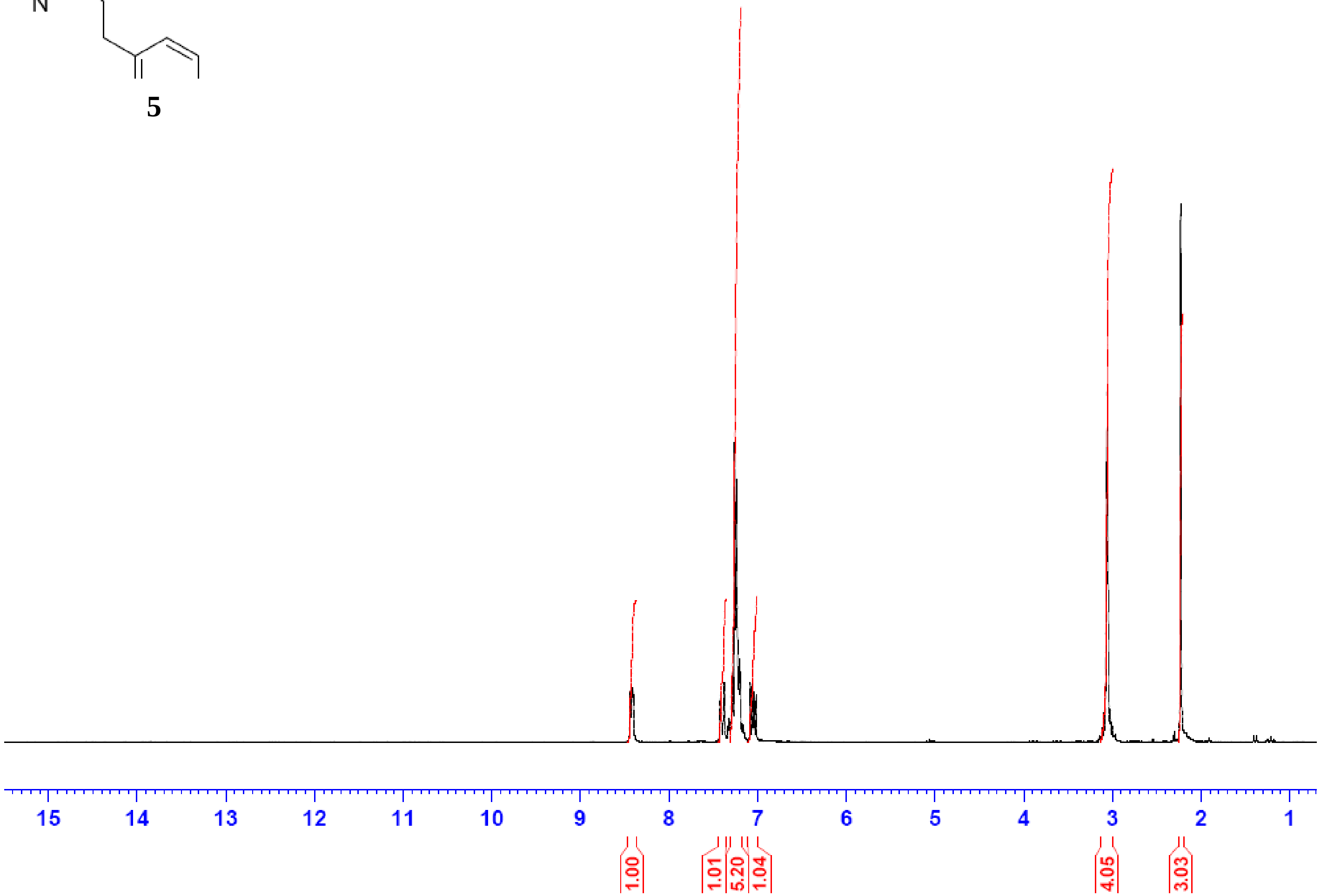

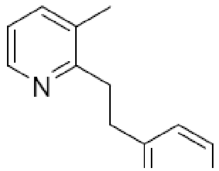
N

5

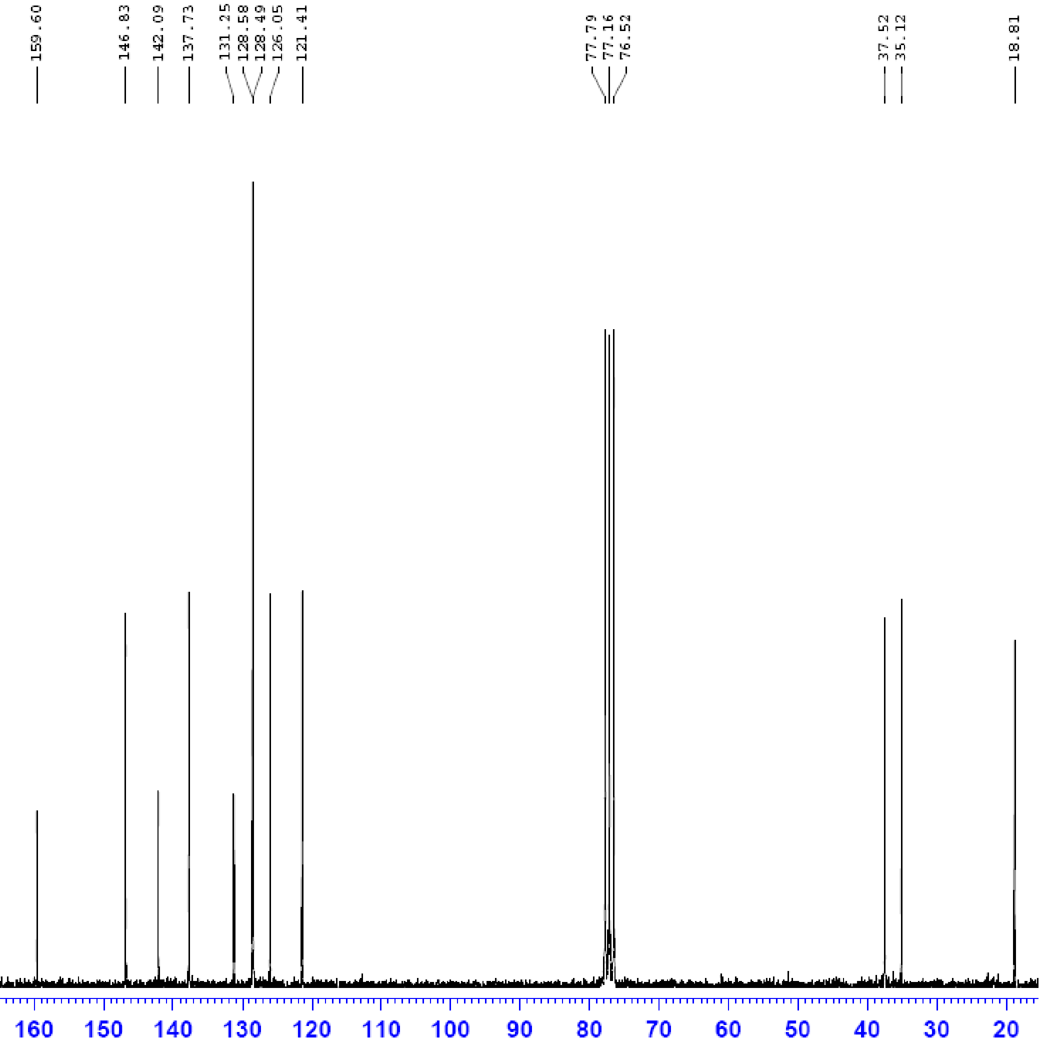
159.60
146.83
142.09
137.73
131.25
128.58
128.49
126.05
121.41
77.79
77.16
76.52
37.52
35.12
18.81
210 200 190 180 170 160 150 140 130 120 110 100 90 80 70 60 50 40 30 20

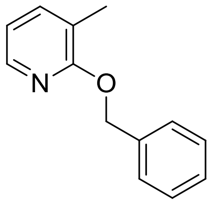

6

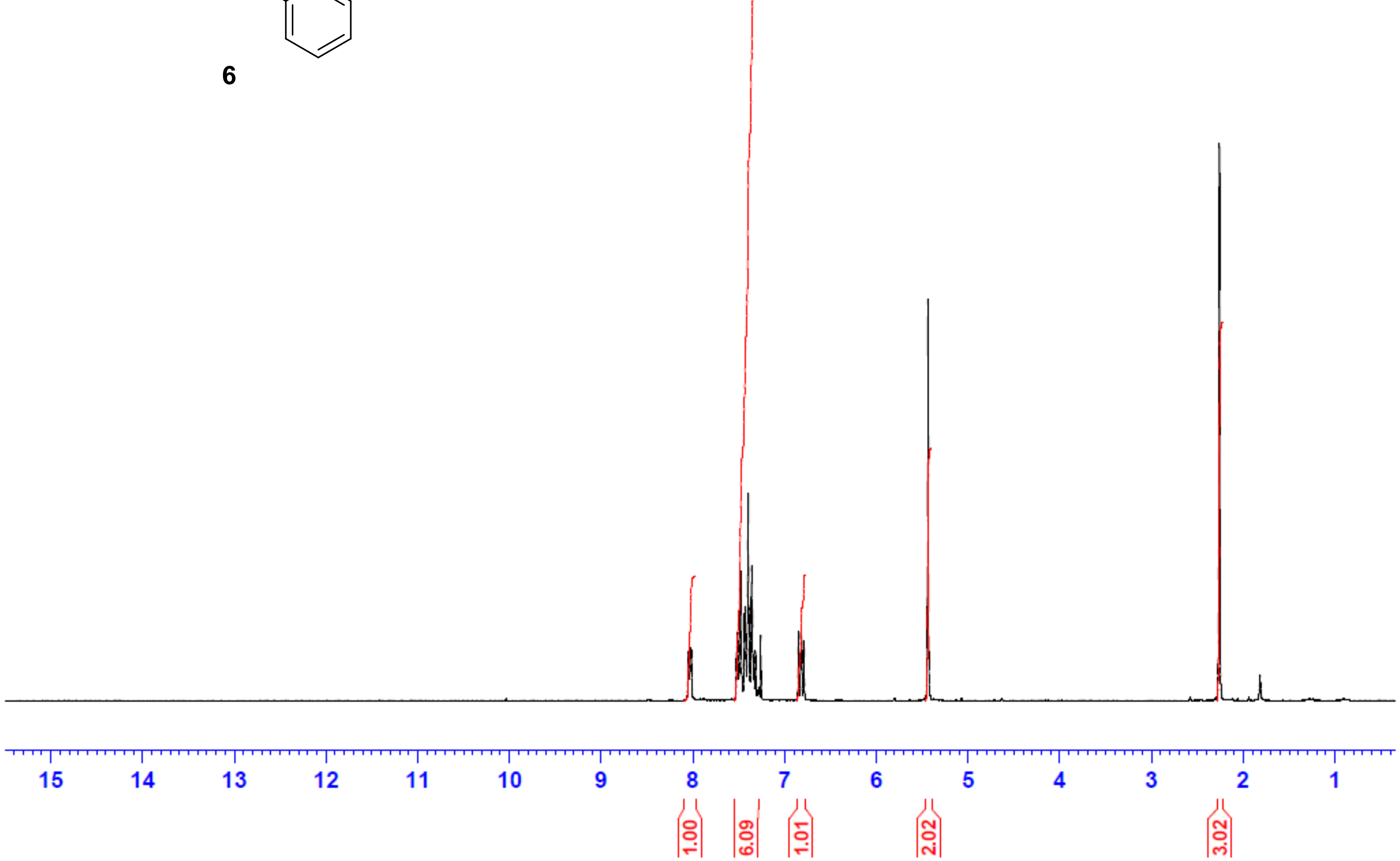

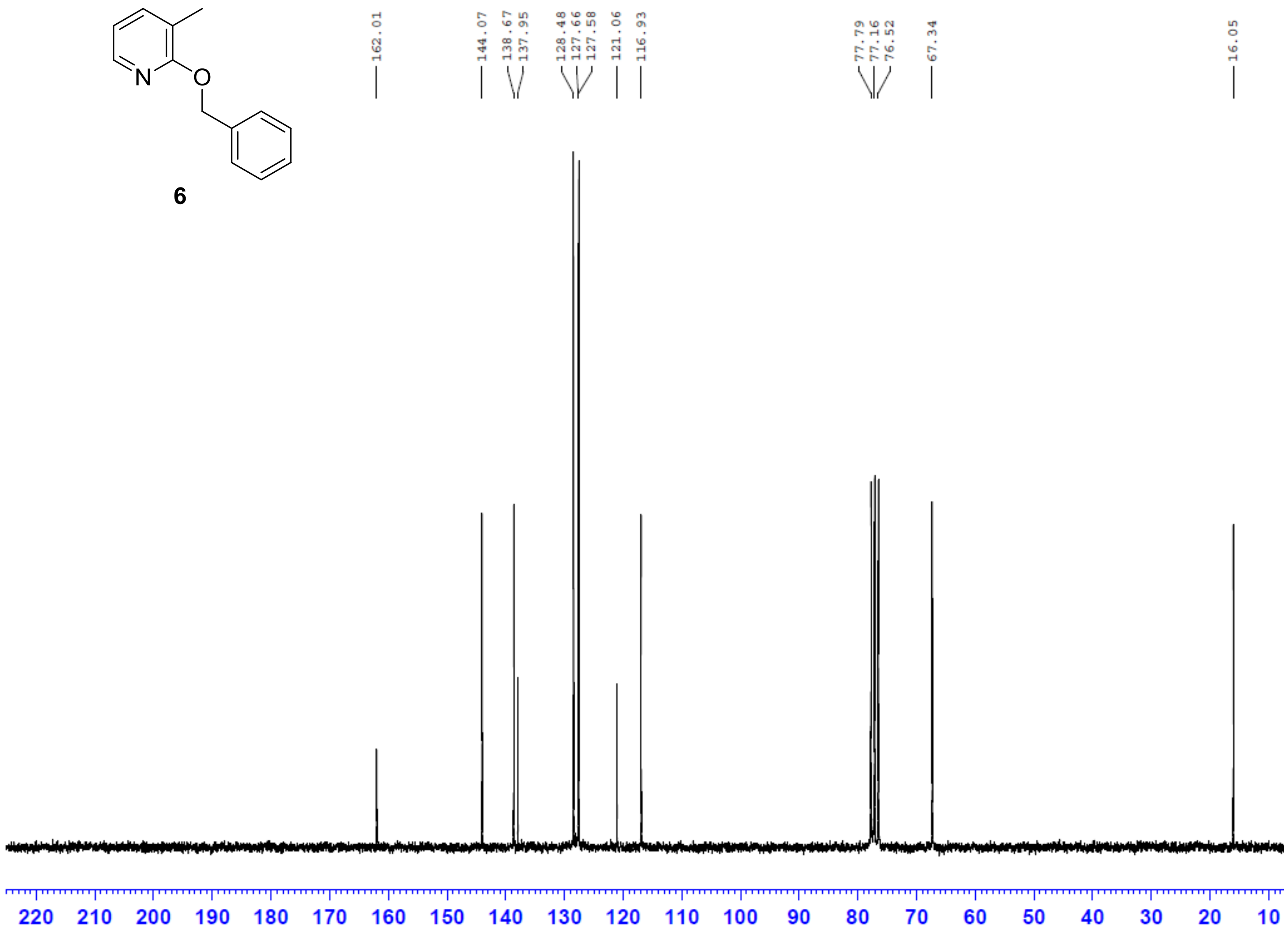

N
O
162.01
144.07
138.67
137.95
128.48
127.66
127.58
121.06
116.93
77.79
77.16
76.52
67.34
16.05
220 210 200 190 180 170 160 150 140 130 120 110 100 90 80 70 60 50 40 30 20 10

6

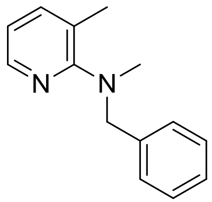

7a

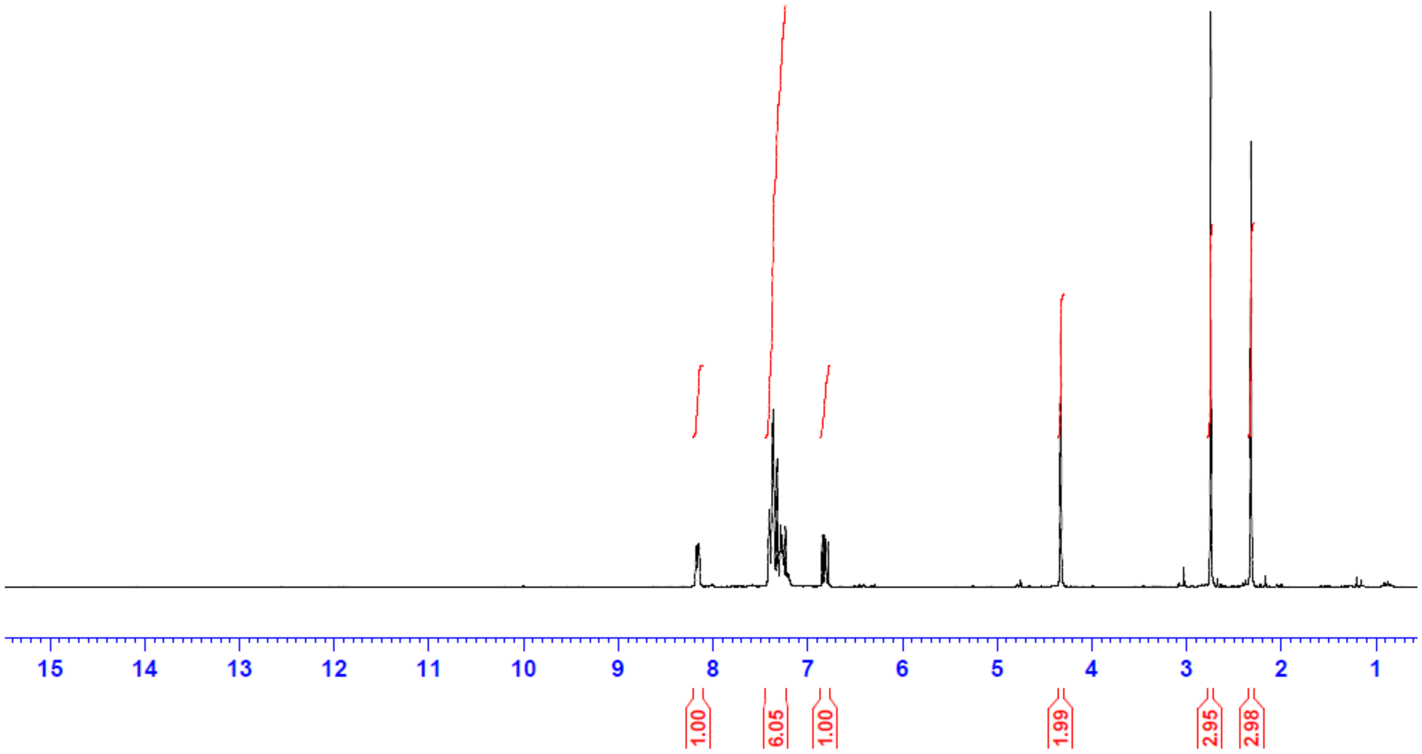

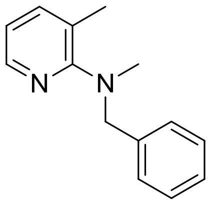

7a

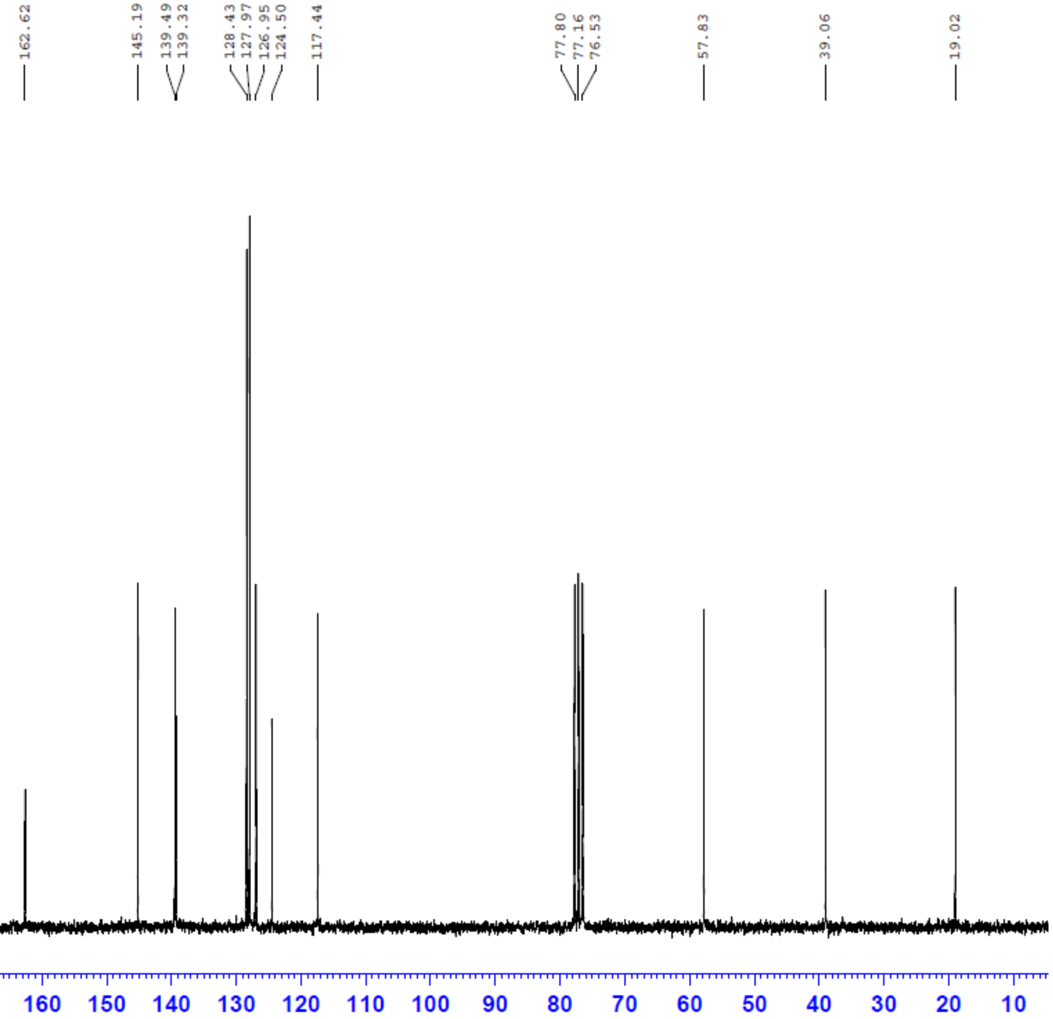
162.62
145.19
139.49
139.32
128.43
127.97
126.95
124.50
117.44
77.80
77.16
76.53
57.83
39.06
19.02
220 210 200 190 180 170 160 150 140 130 120 110 100 90 80 70 60 50 40 30 20 10

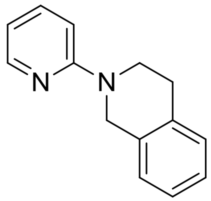

7b

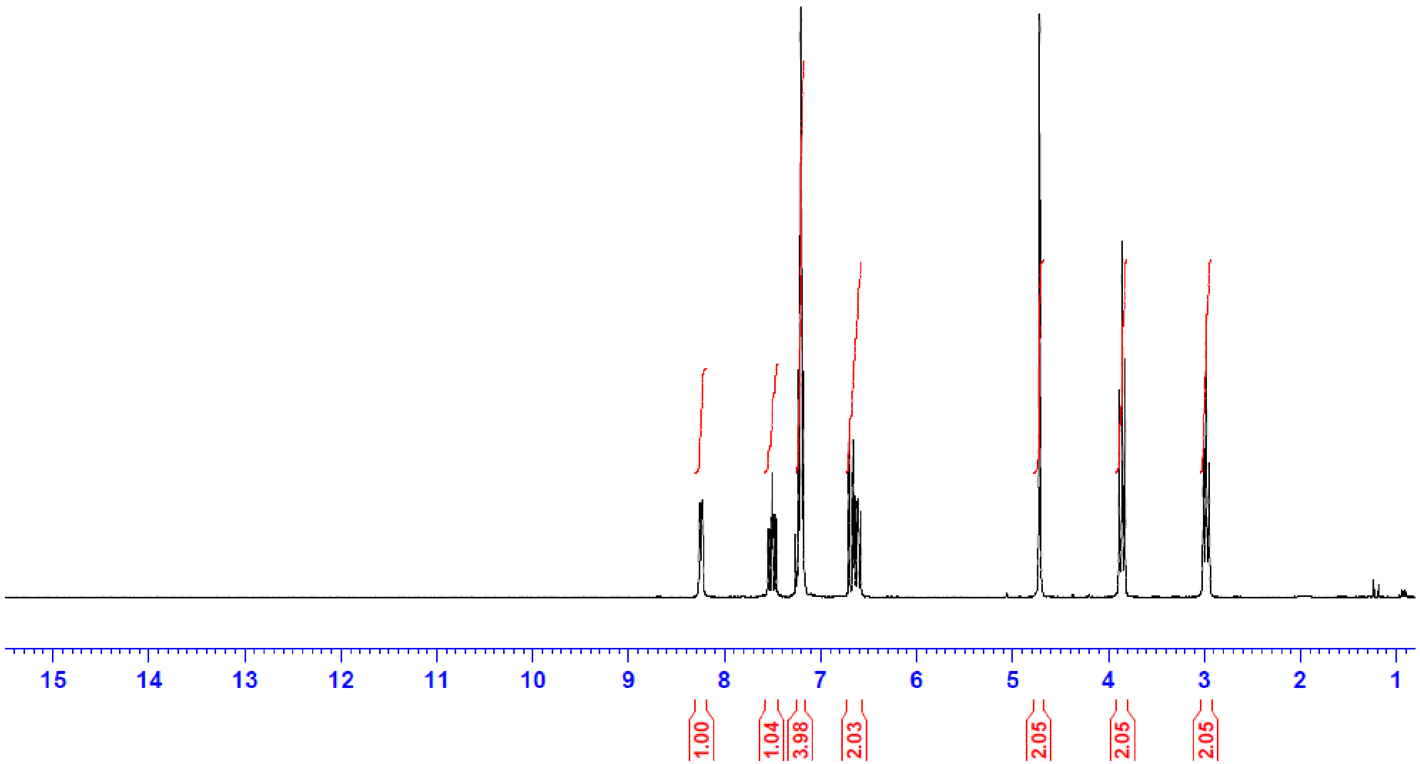

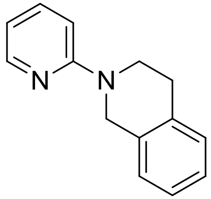
N
N

7b

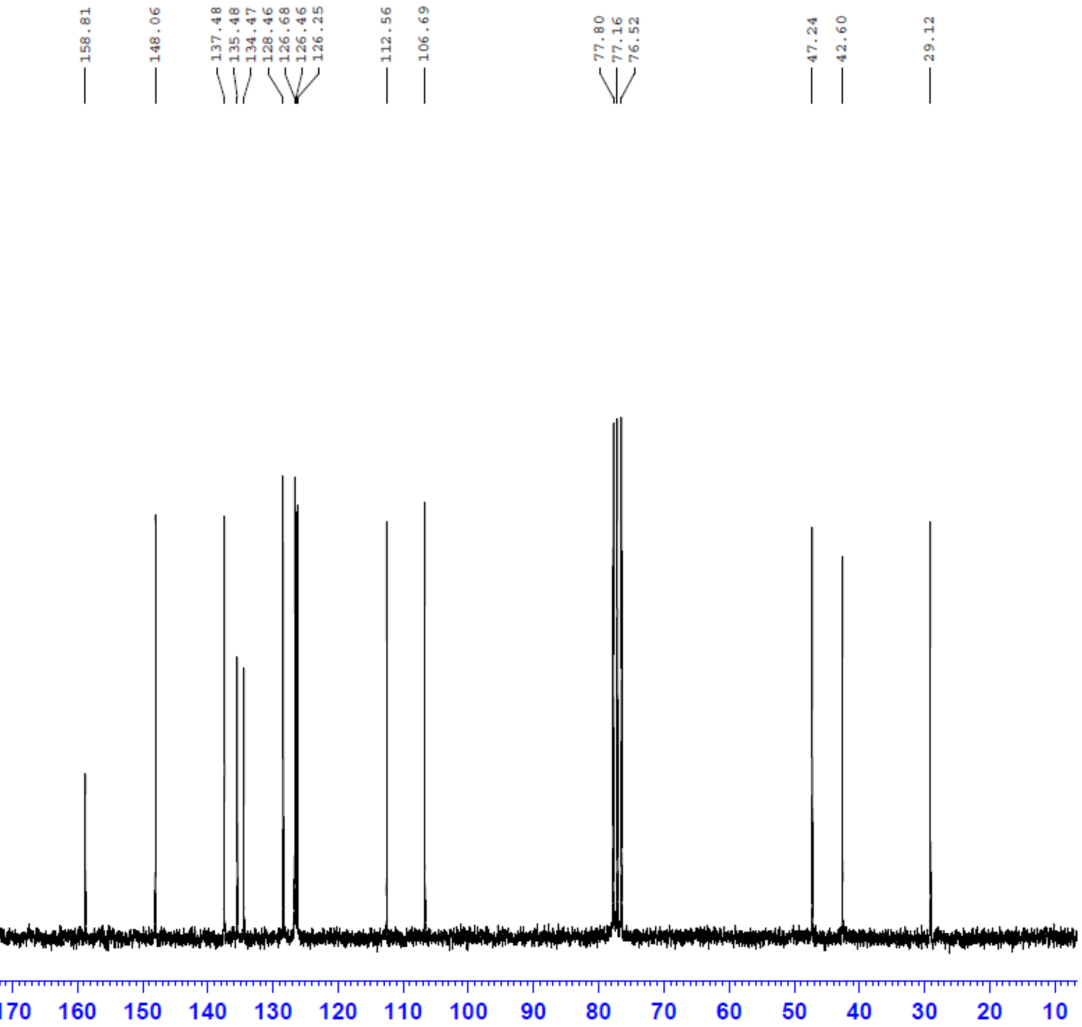
158.81
148.06
137.48
135.48
134.47
128.46
126.68
126.46
126.25
112.56
106.69
77.80
77.16
76.52
47.24
42.60
29.12
220 210 200 190 180 170 160 150 140 130 120 110 100 90 80 70 60 50 40 30 20 10

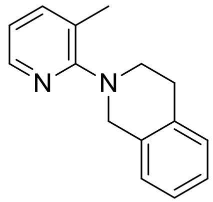

7c

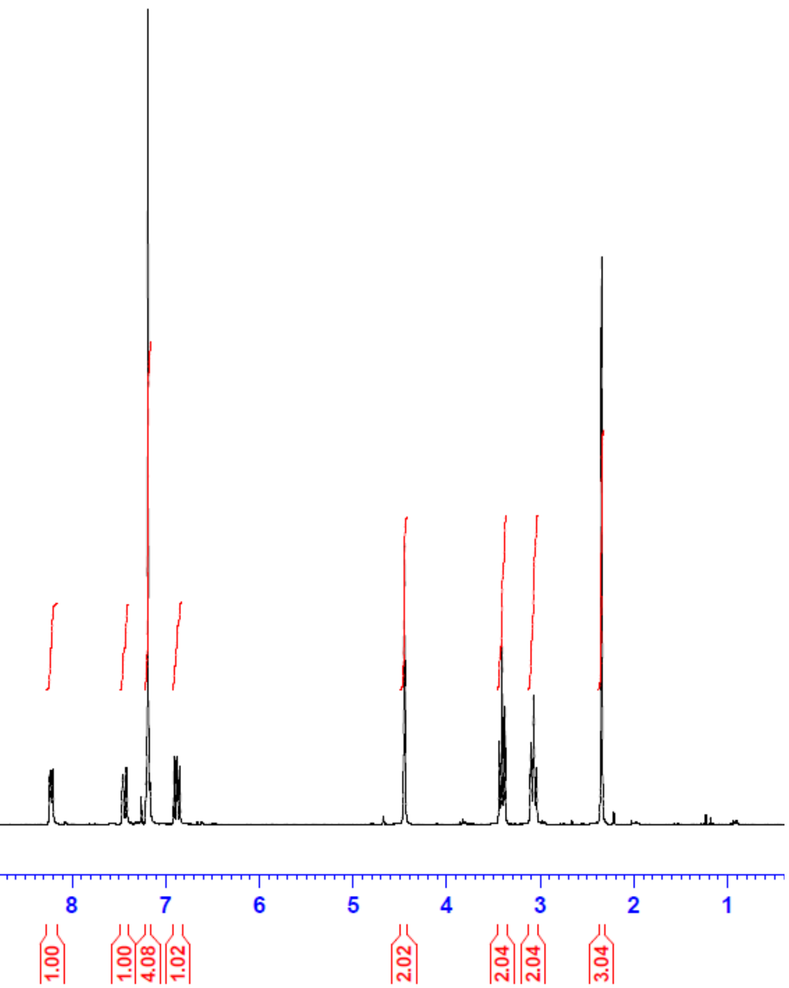

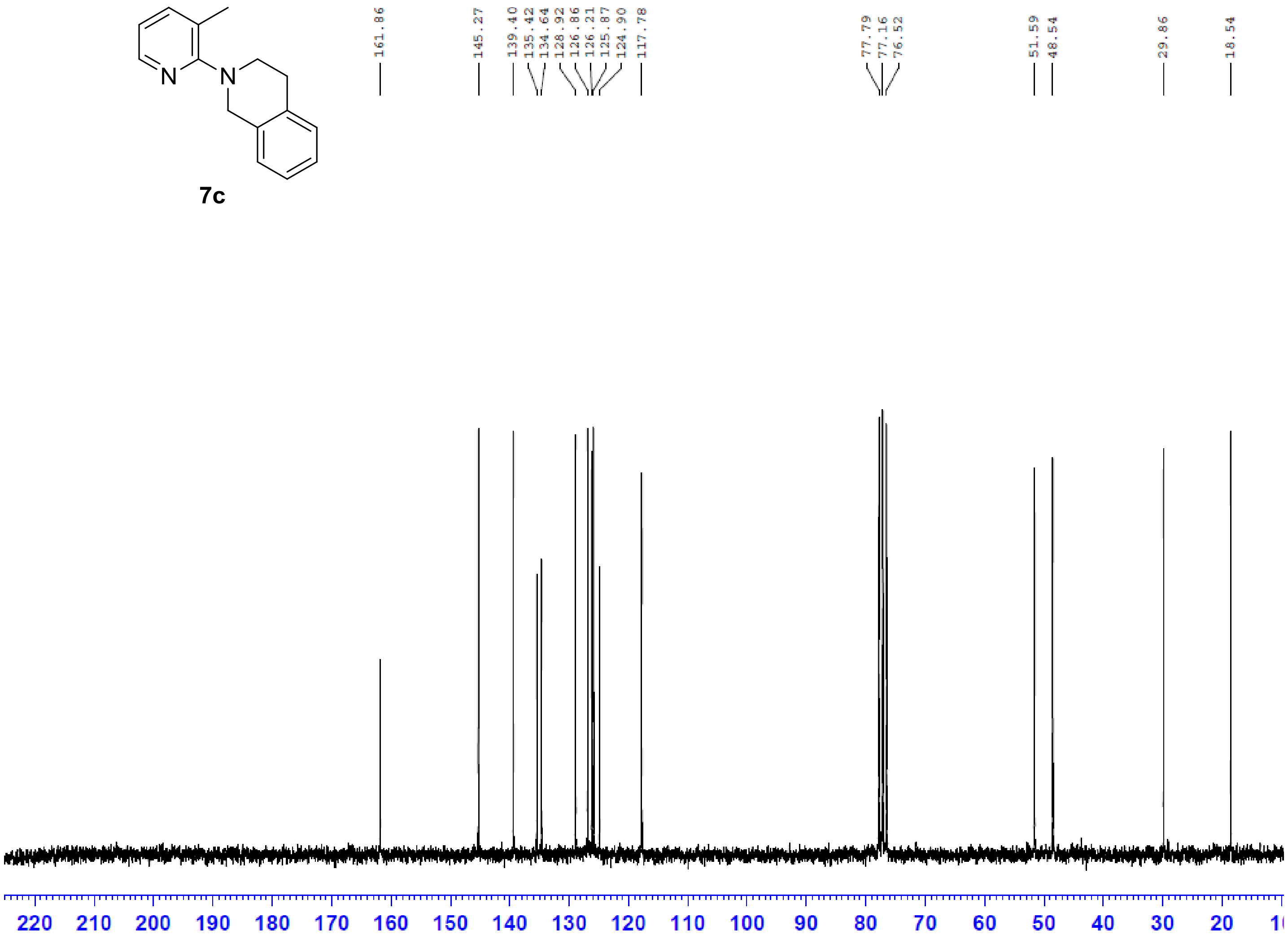
7c
161.86
145.27
139.40
135.42
134.64
128.92
126.86
126.21
125.87
124.90
117.78
77.79
77.16
76.52
51.59
48.54
29.86
18.54
220 210 200 190 180 170 160 150 140 130 120 110 100 90 80 70 60 50 40 30 20
